# Novel Polycondensed Partly Saturated β-Carbolines Including Ferrocene Derivatives: Synthesis, DFT-Supported Structural Analysis, Mechanism of Some Diastereoselective Transformations and a Preliminary Study of their In Vitro Antiproliferative Effects

**DOI:** 10.3390/molecules25071599

**Published:** 2020-03-31

**Authors:** Kinga Judit Fodor, Dániel Hutai, Tamás Jernei, Angéla Takács, Zsófia Szász, Máté Sulyok-Eiler, Veronika Harmat, Rita Oláh Szabó, Gitta Schlosser, Ferenc Hudecz, László Kőhidai, Antal Csámpai

**Affiliations:** 1 Department of Organic Chemistry, Eötvös Loránd University (ELTE) Budapest Pázmány P. sétány 1/A, H-1117 Budapest, Hungary; fodorkinga90@gmail.com (K.J.F.); hutaidani@gmail.com (D.H.); fhudecz@caesar.elte.hu (F.H.); 2 MTA-ELTE Research Group of Peptide Chemistry, Budapest Pázmány P. sétány 1/A, H-1117 Budapest, Hungary; jernei91@gmail.com (T.J.); rita.olah.szabo@gmail.com (R.O.S.); sch@chem.elte.hu (G.S.); 3 Department of Genetics, Cell- and Immunobiology, Semmelweis University, Nagyvárad tér 4, H-1089 Budapest, Hungary; angela.takacs1@gmail.com (A.T.); szaszzsoccii@gmail.com (Z.S.); kohlasz2@gmail.com (L.K.); 4 Laboratory of Structural Chemistry and Biology, Institute of Chemistry, Eötvös Loránd University, Budapest Pázmány P. sétány 1/A, H-1117 Budapest, Hungary; etammate95@gmail.com (M.S.-E.); veronika@chem.elte.hu (V.H.); 5 Department of Analytical Chemistry, Eötvös Loránd University (ELTE), Budapest, Pázmány P. sétány 1/A, H-1117 Budapest, Hungary

**Keywords:** β-carboline, diastereoselectivity, conformation, ferrocene, organic synthesis, NMR spectroscopy, X-ray diffraction, DFT calculations, cytotoxic activity, structure-activity relationships

## Abstract

Use of a Pictet-Spengler reaction of tryptamine and l-tryptophan methyl ester and subsequent reduction of the nitro group followed by further cyclocondensation with aryl aldehydes and formyl–substituted carboxylic acids, including ferrocene-based components, furnished a series of diastereomeric 6-aryl-substituted 5,6,8,9,14,14b-hexahydroindolo[2′,3′:3,4]pyrido[1-*c*]-quinazolines and 5,5b,17,18-tetrahydroindolo[2′,3′:3,4]pyrido[1,2-*c*]isoindolo[2,1-*a*]quinazolin-11-(15b*H*)-ones with the elements of central-, planar and conformational chirality. The relative configuration and the conformations of the novel polycyclic indole derivatives were determined by ^1^H- and ^13^C-NMR methods supplemented by comparative DFT analysis of the possible diastereomers. The structure of one of the pentacyclic methyl esters with defined absolute configuration “*S*” was also confirmed by single crystal X-ray diffraction measurement. Accounting for the characteristic substituent-dependent diastereoselective formation of the products multistep mechanisms were proposed on the basis of the results of DFT modeling. Preliminary in vitro cytotoxic assays of the products revealed moderate-to-significant antiproliferative effects against PANC-1-, COLO-205-, A-2058 and EBC-1 cell lines that proved to be highly dependent on the stereostructure and on the substitution pattern of the pending aryl substituent.

## 1. Introduction

Aromatic or partly saturated β-carbolines can be found as key structural motifs in a variety of biologically active compounds, including both synthetic and natural products that display remarkable pharmacological activities, e.g., antimalarial [1], cardioprotective [2], trypanocidal and neurotoxic [3] effects. A number of compounds belonging to this group of heterocycles have also been identified as potent anticancer agents with marked cytotoxicity against malignant human cell lines [4,5]. Since the last years have witnessed a growing interest in novel chemotherapeutic agents with a multitarget mechanisms of action, it is of pronounced importance that representative tetrahydro-β-carbolynes proved to be inhibitors of microtubule-crosslinking mitotic kinesin Eg5 [6,7], cell division-inducing cyclic dependent kinase (CDKs) [8,9] as well as topoisomerase I and II [8,10]. Pointing to an additional mode of antiproliferative action of β-carbolines, a few representative 9-substituted harmine alkaloids were identified as DNA-intercalators [10]. β-Carboline hybrids with aryl linkers were also shown to exhibit substantial antiproliferative activity on a wide range of malignant human cells acting mainly by CT-DNA intercalation [11]. On the other hand, synergistically strengthening the antiproliferative effects associated with selective binding-based classical targeted mechanisms of action, electron transfer processes via the Fenton pathway induced by redox active fragments, e.g., by suitably positioned ferrocenyl group(s) in the molecular scaffold, may play a key role in mitochondrial generation of reactive oxygen species (ROS), e.g., nitric oxide, superoxide anion and other forms of free radicals [12,13,14] that can be involved in biological regulatory processes finally leading to programmed cell death (apoptosis) [15]. Supporting the relevance of this view, convincing preclinical evidence about the interplay between particular binding-induced- and redox signalling pathways implicated in cancer has been disclosed [16]. Accordingly, due to their marked effects on malignant cell lines, a plethora of ferrocene derivatives with diverse molecular architectures have been emerged as potent antiproliferative agents in the last decades [17,18,19,20,21].

## 2. Results and Discussion

**Figure 1:**
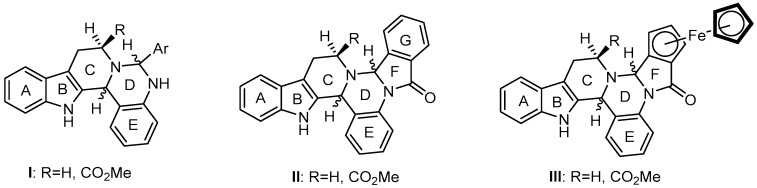
Three types of the targeted polycyclic β-carbolines with diverse streostructures derived from tryptamin (R = H) and l-tryptophan (R = CO_2_Me).

Prompted by the aforementioned highly promising precedents, in the frame of our ongoing research aimed at identification of novel leads including ferrocene hybrids [22,23,24,25,26,27,28,29] we envisaged atryptamine- and tryptophan-based synthesis, detailed structural analysis and preliminary in vitro evaluation of 6-aryl-substituted 5,6,8,9,14,14b-hexahydroindolo[2′,3′:3,4]pyrido[1,2-*c*]quinazolines (**I**), 5,5b,17,18-tetrahydroindolo[2′,3′:3,4]pyrido[1,2-*c*]isoindolo[2,1-*a*]quinazolin-11(15*bH*)-ones (**II**), and (*S*_p_)-2-formyl-1-ferrocenecarboxylate-derived 5,5b,11,14b,16,17-hexahydroindolo[2′,3′:3,4]py- rido[1,2-*c*]ferroceno[*c*]pyrrolo[1,2-*a*]quinazoline-11(14b*H*)-ones (**III**), featuring alkaloid-like frameworks with diverse stereostructures (Figure 1).

### 2.1. Synthesis and Structural Analysis of the 6-Aryl-Substituted 5,6,8,9,14,14b-Hexahydroindolo[2′,3′:3,4]-pyrido[1,2-c]quinazolines

**Scheme 1:**
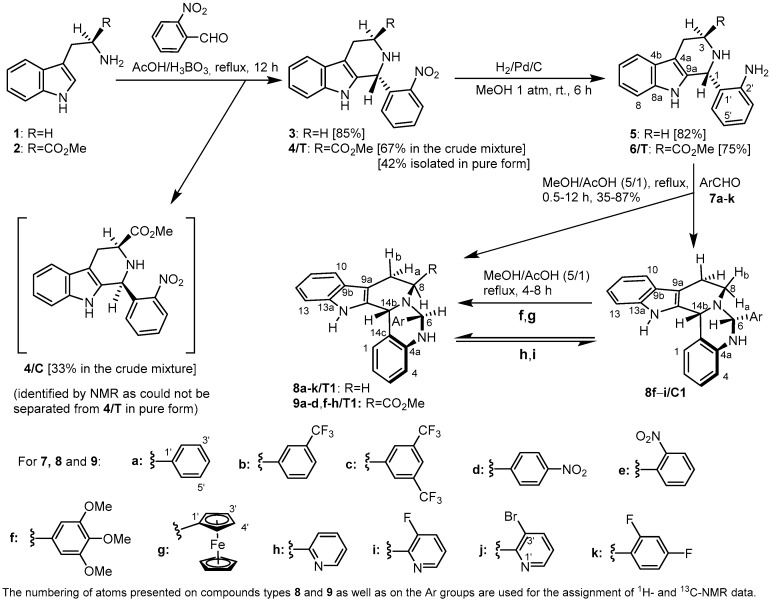
Synthetic routes to novel diastereomeric 6-aryl-5,6,8,9,14,14b-hexahydroindolo-[2′,3′:3,4]pyrido-[1,2-*c*]quinazolines (the relative/absolute configuration is presented for compounds with R = H/R = CO_2_Me.).

A straightforward retrosynthetic analysis of pentacycles type **I** set up an obvious synthetic pathway starting with a Pictet-Spengler (PS) annelation involving tryptophan-based precursors and 2-nitrobenzaldehyde followed by nitro group reduction and subsequent aldehyde-mediated cyclisation of the resulting 2-aminophenyl-substituted β-carboline framework to construct the targeted pentacyclic products (Scheme 1). Accordingly, tryptamine (**1**) was first converted into the nitrophenyl derivative **3** by an efficient PS protocol using an acetic acid/boric acid system at reflux temperature to promote the reaction [30] which practically went to completion within 4 h and allowed the isolation of the product as a racemic mixture in 85% yield. The analogous reaction of the methyl ester of l-tryptophan (**2**), conducted under the same conditions, gave a hardly separable 1/2 mixture of *cis*- and *trans*-diastereomer esters **4/C** and **4/T**. By means of flash column chromatography on silica using CH_2_Cl_2_-MeOH (80:1) as eluent, **4/T** could be isolated in pure form in acceptable yield (42%), but the complete separation of **4/C** from **4/T** could not be achieved.

**Figure 2:**
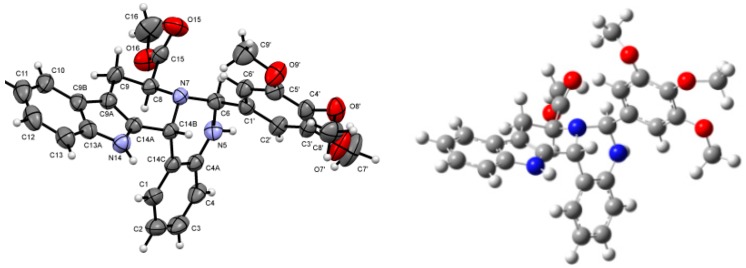
Stereostructure of *trans* pentacyclic esther **9f**/**T1** obtained by single crystal X-ray diffraction (**left**) and DFT modelling (**right**).

The Pd-catalysed hydrogenation of **3** and **4/T** gave the expected 1-(2-aminophenyl)-substituted β-carbolines (racemic **5** and enantiopure **6/T** in 82% and 75%, resp.) which were then reacted with aromatic aldehydes **7a**–**k** in a 5/1 mixture of MeOH and AcOH at reflux temperature affording tetracycles of type **8** and **9**. The facile cyclisations of **6/T** could be completed within 2 h providing most of the targeted esters **9a**–**d**,**f**–**h/T1** as single enantiomers that were isolated in 62–73% yield (Table 1) with a well-defined stereostructure, that was confirmed for **9f/T1** by single-crystal X-ray diffraction (Figure 2).

Employing prolonged reaction times the isolated yields could not be increased as the ring closures were accompanied by decomposition. (**T1** is an arbitrary designation of the relative configuration as will be discussed later along with **T2** and **T3** representing two further types of possible diastereomers.) The unanimous and rigid conformation of **9a**–**d**,**f**–**h/T1**, determined by the carbon- and nitrogen stereogenic centres (C-6, C-8, C-14b and the non-inverting N-7) as well as by the conformational chirality of ring *C*, is clearly reflected from the highly similar (practically almost identical) ^1^H- and ^13^C-NMR parameters obtained for the skeletal atoms of this series of esters (cf. Experimental).

Of note is the fact that diagnostic interactions detected between proton pairs H-6/H-8 and H-14b/H-2′,6′ in the NOESY spectra of **9a**–**d** also confirm the *trans* arrangement of H-6 and H-14b as well as the spatial proximity of H-6 and H-8. In **9a**–**d**,**f**–**h/T1** the axial position of H-8 thus, the equatorial position of the methoxycarbonyl group (R), can also be regarded as evidenced by the characteristic coupling pattern of the signals originated from the skeletal protons H-8, H-9_a_ and H-9_b_ featuring a dd split of H-8 signal with coupling constants at around 8 Hz and 4 Hz.

The reactions of **6**/**T** with aldehyde components **7e**,**i**–**k** carrying substituents adjacent to the formyl group gave highly complex mixtures of undefined components. The failure of these experiments can probably be attributed to an interference of the CO_2_Me group with the negatively polarized carbonyl oxygen and the aromatic ring, the two rotating molecular fragments that avoid to get in proximal position destabilized by steric crowding (in case of bulky **7e**,**j**) or electrostatic repulsion (in case of **7i**,**k** with negative fluorine-centre).

The reactions of precursor **5** with benzaldehydes **7a**–**k** were carried out under the same conditions using MeOH-AcOH (5:1) as solvent at reflux temperature. Indicating the acceleration of ring closures **5**+**7b**–**e**,**j**,**k**→**8b**–**e**,**j**,**k/T1** enabled by the electron-withdrawing substituents on the aldehyde components, no significant change could be detected in the yields of the products when the reaction was conducted for a substantially longer time (1 h: 65–80%, 12 h: 63–87%). In keeping with this observation, in the lack of activation effect induced by electron-withdrawing group on the aryl ring, the diastereoselective transformation **5**+**7a**→**8a/T1** needed a significant extension of reaction time to produce a substantially increased isolated yield (1 h: 37%, 12 h: 77%). Except for the non-precipitated fraction of the products no component with well-defined structure could be unequivocally identified by NMR in the highly complex mixtures recovered from the solutions. The stereostructure of **8a**–**e**,**k**/**T1** determined by the helicity of ring *C* and the relative configuration of the stereogenic centres C-6, N-7 and C-14b was evidenced by the NOESY interactions detected between H-14b and the protons of the 6-Ar group as well as by the highly similar chemical shifts of ^1^H/^13^C signal pairs H-6/C-6 and H-14b/C-14b discernible in narrow ranges of the appropriate spectra of compounds type **8**/**T1** verifying to their unanimous skeletal structure. Pointing to the spectacular substituent-dependence of the diastereoselectivity of the studied cyclocondensations, on short treatment (0.5 h) with aldehydes **7f** and **7g** containing electron-donating aryl group, **5** got converted into pentacycles **8f**/**C1** (63%) and **8g**/**C1** (39%), respectively, in which H-6 and H-14b are in *cis* position on ring *D* as proved by NOESY measurements. Besides small portions of products type **8/C1**, traces of their *trans* counterparts (**8f**,**g**/**T1**) could also be identified by NMR in the complex mixtures recovered from the solution. When aldehyde **7f** was used as reactant, the extension of reaction time from 0.5 h to 4 h gave rise to a spectacular change in the product ratio resulting in the isolation of the epimeric mixture of **8f**/**C1** and **8f**/**T1** in 80% yield markedly enriched in the *trans* component (**C1/T1**~1/3). Further prolongation of the reaction time to 8 h led to the isolation of an approximately 1/4 mixture of these diastereomers with slightly decreased isolated yield of 75%, pointing to a slow development of an equilibrium system. On the other hand, undefined decomposition processes prevented a substantial extension of the reaction time of the formylferrocene-mediated cyclisation of **5**, thus 4 h was found to be an optimal choice allowing the isolation of the mixture of diastereomers **8g**/**C1** and **8g**/**T1** in 52% yield enriched in the *trans* component (**C1/T1**~3/7). A series of attempts for the chromatographic separation of isomer pairs **8f**/**C1**_**8f**/**T1** and **8g**/**C1**_**8g**/**T1** failed.

It is of interest that even with only 0.5 h of treatment of **5** with formylpyridines **7h**,**i** got readily converted into approximately 4/3 and 1/1 mixtures of isomer pairs **8h**/**C1**_**8h**/**T1** and **8i**/**C1**_**8i**/**T1**, respectively, in high yields (72% for **8h**/**C1**_**8h**/**T1** and 80% for **8i**/**C1**_**8i**/**T1**). Employing substantially prolonged reaction time (12 h) the ratio of the diastereomers were not changed within experimental error pointing to the fast formation of the rapidly interconverting isomers with slightly different or nearly identical thermodynamic stability, while both diastereomer pairs could be isolated in dramatically decreased yields (25% for **8h**/**C1**_**8h**/**T1** and 35% for **8i**/**C1**_**8i**/**T1**) from the darkened reaction mixtures indicating uncontrolled decompositions of these pyridine derivatives. On the other hand, using 3-bromopicolinaldehyde **7j**, a sterically more demanding ring-closing component, the reaction of **5** practically was completed within 1 h to afford *trans* diastereomer **8j/T1** isolated as single product in high yield (80%). Since after an extended reaction time (12 h) this *trans* diastereomer could again be isolated in excellent yield (87%), it seems reasonable that the abovementioned decomposition of the equilibrating isomer pars of **8h**,**i** might take place via the appropriate *cis* isomer.

The experimental product distributions corroborate with the relative free energy [ΔG(**8/T1**-**8/C1**)] values (Table 2) obtained by DFT comparative analysis of the isomeric pairs carried out by B3PW91 functional [31] employing DGTZVP basis set [32] together with IEFPCM solvent model [33]. For all calculations, a dielectric constant (ε = 28.2) was used to represent the approximate polarity of the medium composed of the solvent mixture MeOH-AcOH (5:1). It must be noted here that the comparative DFT modeling studies were performed on models also including ferrocene derivatives, so we selected B3PW91 for this purpose as this functional has been demonstrated to provide an improvement over B3LYP regarding the description of bonding parameters in metal-containing fragments with metal-carbon bond(s) [34].

**Scheme 2:**
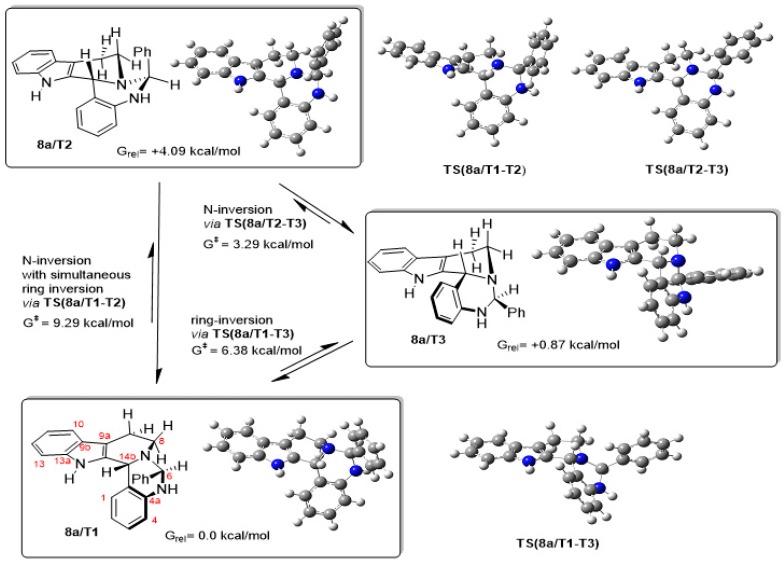
Schematic representations and optimized structures of the possible diastereomers of pentacycle **8a/T1** characterised by relative free energy values and barriers of their interconversion proceeding via the presented TS structures as disclosed by a series of DFT modelling studies.

Although the relative *trans* position of protons H-6 and H-14b in compounds types **8** and **9** was unambiguously evidenced by NOESY experiments disclosing interactions between H-14b and the protons of the proximal Ar groups, the possible N-7-inversion with simultaneous or separated flip of ring *C* might allow the formation of equilibrium mixtures of three diastereomers designated as T1, T2 and T3 (Scheme 2). Their interconversions, in principle enabled by the abovementioned processes, were modelled by the DFT analysis of **8a**. The computations identified these types of conformers as three local minima on the potential energy surface (PES) connected by saddle points representing the corresponding transition states **TS(8a/T1-T2)**, **TS(8a/T2-T3)** and **TS(8a/T1-T3)** (Scheme 2).

Although the calculated activation barriers (Scheme 2) might point to a relative flexibility of the skeletal structure, in agreement with the relative energetics expressed in Gibbs free energy values, the NMR data discussed above clearly indicate that **8a/T1** can be regarded as the most stable and practically exclusively detectable diastereomer in the analysed sample dissolved in DMSO-*d*_6_.

**Scheme 3:**
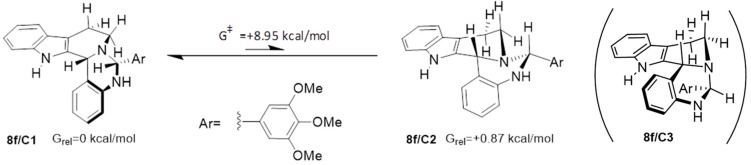
Structures, relative Gibbs free energy values of **8f**/**C1** and **8f**/**C2** and the activation barrier of the isomerisation of **8f**/**C1** into **8f**/**C2** taking place by N7-inversion with simultaneous flip of ring C.

It is of importance that on the basis of the well-established solid-state structure of **9f** supplemented by the results of ^1^H- and ^13^C-NMR studies that disclosed highly similar chemical sifts and H-H coupling patterns for the skeletal CH- and CH_2_ groups, it can be stated that all the isolated pentacyclic products types **8** and **9** with *trans* relative configuration preferably adopt conformation T1. Supporting this view about the conformational space, the same series of DFT analyses carried out for esther **9a** identified the expected diastereomers **9a**/**T1**, **9a**/**T2** and **9a**/**T3** along with their relative energetics and activation barriers of their interconversions proceeding through transition states **TS(9a/T1-T2)**, **TS(9a/T2-T3)** and **TS(9a/T1-T3)**. Albeit in the minor components **9a**/**T2** and **9a**/**T3** the methoxycarbonyl group is in axial position, the calculated relative energetics [ΔG(**9a**/**T1**-**9a**/**T2**) = +4.53 kcal/mol and ΔG(**9a**/**T1**-**9a**/**T3**) = +0.87 kcal/mol] refer to a population distribution similar to that of the conformers of **8a**. We also undertook the conformational analysis of the detectable or purely isolated *cis* pentacyclic products. In **8f**–**i/C1** diagnostic NOE could be detected between protons H-14b and H-6. Conformation C1 was identified by further comparative DFT studies carried out on representative model diastereomers **8f**/**C1** and **8f**/**C2**. Besides the relative energetics [ΔG(**8f**/**C1**-**8f**/**C2**) = +0.89 kcal/mol] diagnostic ^1^H- and ^13^C-chemical shifts were also calculated for the optimized structures of these isomers and compared to a selection of diagnostic experimental data (calcd. for **C1**/**C2**/*measured* [ppm]: δH-14b = **5.70**/5.95/*5.61*; δH-6 = **5.80**/6.20/*5.62*; δH-1 = **7.50**/8.22/*7.21*; δC-14b = **60.5**/63.3/*57.1*; δC-6 = **79.1**/84.6/*73.3*; δC-8 = **39.6**/50.7/*38.0*) suggesting that **8f**/**C1** is present in the DMSO-*d*_6_ solution subjected to NMR measurements. These calculations were performed by GIAO method [35] using B3LYP functional [36] and 6-311++G(2d,p) basis set [37]. Due to the significant steric crowdance in the *endo* region of the bent ring system, **8f**/**C3** was not taken into account as a realistic component of the conformational space (Scheme 3). These considerations about the skeletal conformation can also be extended to the further detected *cis* products **8g**–**i**.

### 2.2. Preparative-, Spectral and Theoretical Studies on the Mechanism and Products of the Substituent-Controlled Annulations of 5 and 6/T Effected by Formyl-Substituted Carboxylic Acids

**Scheme 4:**
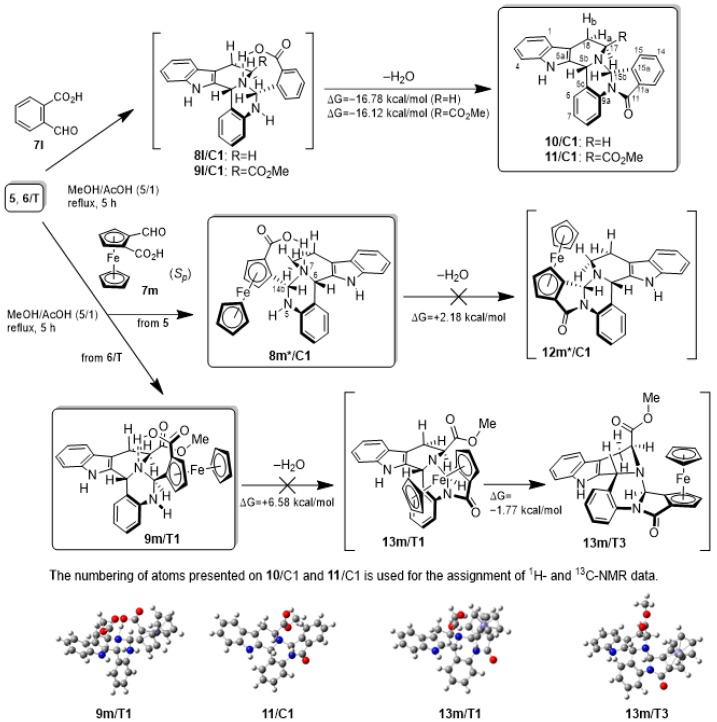
Cyclization reactions of 1-(2-aminophenyl)-2,3,4,9-tetrahydro-1*H*-pyrido[3,4-*b*]indoles **5** and **6/T** effected by 2-formylbenzoicacid and (*S*_p_).2-formylferrocenecarboxylic acid **7l** and **7m**, respectively. A selection of optimized structures with absolute configuration obtained by DFT modelling.

In order to obtain targeted polycyclic lactams types **II** and **III** (Figure 1), using our standard conditions with slightly prolonged reaction time (5 h), precursors **5** and **6/T** were also reacted with 2-formylbenzoicacid (**7l**) and the enantiomerically pure planar chiral (*S*_p_)-2-formyl-1-carboxyferrocene (**7m**), respectively (Scheme 4).

The reactions of **5** and **6**/**T** with **7l** afforded polycondensed lactams **10/C1** and **11/C1** in good yields, (88% and 71%, respectively,) pointing to fast sequential annelations that certainly proceed via the appropriate *cis* pentacyclic diastereomer (**8l/C1** and **9l/C1**). However, on treatment with **7m** the racemic precursor **5** got converted into *cis* pentacyclic product 8m*/**C1** in mediocre yield (47%) with well-defined relative and, consequently, absolute configuration ultimately controlled by the planar chiral ferrocene unit embodied in the heterocyclic skeleton. This relatively rigid molecular architecture is stabilized by an intramolecular H-bond involving the carboxylic group and the *N*-7 atom of which basic character is probably enhanced relative to that of *N*-5 atom. Taking this seven-membered chelate ring and the *S*_p_ planar chirality of the ferrocene moiety as structural constrains into account, the absolute configuration of 8m*/**C1** was deduced from the NOESY interactions of the unsubstituted Cp ring with the proximal (C)H-14b and (N)H-5 protons.

Accordingly, the proximity of the iron centre can also be considered as evidenced by the substantial downfield shift of the H-14b signal (6.23 ppm) relative to that measured for the racemic ferrocenyl-substituted derivative **8g**/**C1** (5.49). The skeletal structure and the relative- and consequently, the absolute configuration of l-tryptophan-derived **11**/**C1** were disclosed by ^1^H- and ^13^C-NMR methods. The presence of the fused isoindolone fragment was evidenced by the HMBC correlation between the signals of H-15b and C-11.

The characteristic coupling pattern of the signals of the skeletal protons H-17, H-18_a_ and H-18_b_ featuring a dd split for H-17 resonance (*J* = 7.9 Hz and 3.8 Hz) and the highly spectacular upfield shift of the OCH_3_ singlet (2.83 ppm) can be regarded as conclusive evidences for the equatorial position of the CO_2_CH_3_ group on ring *C* being in the anisotropic shielding region of ring *D*. This proximal position of the CO_2_CH_3_ group and ring *D* was also confirmed by the diagnostic NOE’s detected between signal pairs H-5b/H-15b and H-15/OCH_3_. Accordingly, the HMBC correlation and the NOE’s involving signal pairs H-15b/C-11 and H-5b/H-15b, respectively, complemented with the highly similar ^1^H- and ^13^C-NMR data, clearly indicate that the racemic lactam **10**/**C1** and its ester analogue **11**/**C1** with well-defined absolute configuration have identical skeletal constitution and conformation.

The reaction of **6**/**T** and **7m**, the two components used as single enantiomers, proved to be sluggish resulting in the formation of *trans* ferrocenecarboxylate **9m**/**T1** in low yield (ca. 10–15% calculated for its approximate 60% ratio in the isolated highly contaminated crude product). Due to its decreased stability and a hardly separable array of undefined contaminations, the attempted purifications of this ester by column chromatography and crystallisation have failed so far. It is also of note that on prolongation of the reaction time this compound underwent a complete decomposition. On the other hand, its skeletal structure could be identified by NMR measurements producing ^1^H and ^13^C chemical shifts and H-H coupling constants characteristic for esters type **9**/**T1** with unambiguous relative- and consequently, absolute configuration.

Contrary to intermediate carboxylates **8l**/**C**1 and **9l**/**C1**, the ferrocene analogues 8m*/C1 and **9m/T1** could not be forced to undergo intramolecular acylation even after using a prolonged reaction time (12 h), instead, uncontrolled processes were promoted resulting in the formation of substantial amounts of tarry materials that allowed the isolation of unchanged pentacycle 8m*/C1 with significantly decreased yield (8%), while the *trans* isomer **9m/T1** completely decomposed. The resistance of these carboxyferrocene derivatives to cyclization can probably be ascribed to the substantial overall strain and steric crowdance in the hypothetic polycyclic ferrocene lactams (12m*/**C1**, **13m**/**T1** or **13m**/**T3**, a more stable conformer) incorporating two diectly fused five-membered rings. This view is strongly supported by the results of DFT modelling studies revealing dramatic reactivity difference between the carboxyphenyl-substituted intermediates (**8l**/**C1** and **9l/C1)** and the carboxyferrocenyl analogues (8m*/**C1** and **9m**/**T1**) in terms of their propensity to undergo cyclisation, as spectacularly shown by the changes in the free energy values calculated for the corresponding transformations (Scheme 4).

### 2.3. Proposed Mechanism for the Rationalization of the Substituent- and Time-Dependent Interconversion of cis- and trans-Pentacycles 8/C1 and 8/T1 Based on the Results of DFT Modelling Studies

**Scheme 5:**
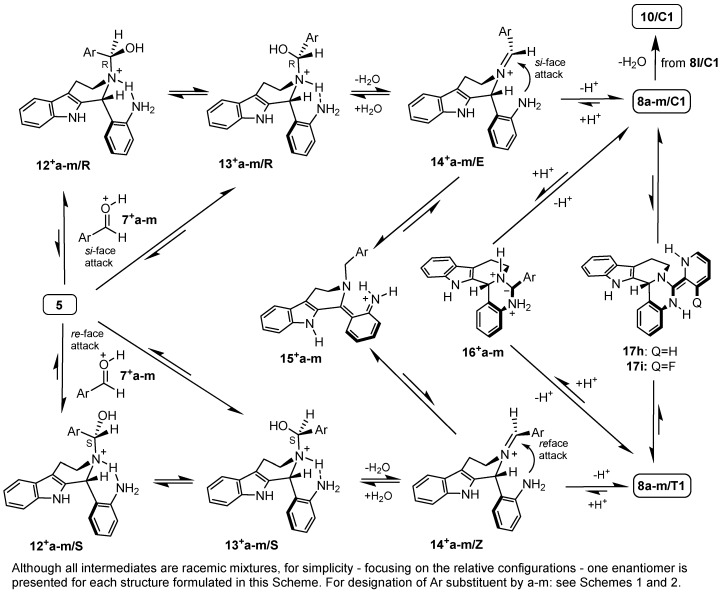
Possible pathways with elementary steps suitable for the interpretation of the experimentally observed substituent-controlled aldehyde-mediated diastereoselective transformations of **5** into pentacyclic products (**8**/**C1** and **8**/**T1**) and hexacyclic lactam **10**/**C1** as suggested by comparative DFT modelling.

Finally, we put forward a rationalization of the characteristic substituent- and time-dependent diastereoselectivity observed in the aldehyde-mediated cyclization reactions of precursor **5** assumed to proceed along the multistep reaction sequences outlined in Scheme 5. It was hypothesized that preferably the nitrogen atom in ring *C* of enhanced nucleophilicity might initiate a *re*- or *si-face* attack on the *O*-protonated aldehyde component to construct interconverting rotamer adduct pairs **12^+^**/**R**_**13^+^/R** and their diastereomer counterparts **12^+^**/**S**_**13^+^/S**. In the subsequent step the rotamers **13^+^/R** and **13^+^/S**, containing N-H and C-OH fragments in approximate *anti* position preformed for *trans* elimination, get converted into iminium cations **14^+^/E** and **14^+^**/**Z**, the key intermediates capable of undergoing either ring closure constructing diastereomeric pentacycles **8/C1** and **8/T1**, or interconversion by reversible 1,3-proton-migration proceeding via the same iminium cation **15^+^**.

In order to check the plausibility of this complex mechanism, comparative DFT studies were carried out on diastereomer pairs of adducts and iminium ions (**12^+^a**,**d**,**f**,**g**/**R**_**12^+^a**,**d**,**g**/**S, 13^+^a**,**d**,**f**,**g**/**R**_**13^+^a**,**d**,**f**,**g**/**S** and **14^+^a**,**d**,**g**/**E**_**14^+^a**,**d**,**f**,**g**/**Z**, respectively) and isomer iminium cations **15^+^a**,**d**,**f**,**g**, a set of reasonably selected models containing substituents of markedly different electronic properties and steric bulk (Table 3).

The calculations were carried out again at B3PW91/DGTZVP level of theory supported by the IEFPCM solvent model using a dielectric constant (ε = 28.2) to represent the polarity of the reaction mixture MeOH-AcOH (5:1). The calculated **Δ**G(**12^+^**/**R**−**12^+^**/**S**) and **Δ**G(**13^+^**/**R**−**13^+^**/**S**) suggest that the transformation with electron-deficient 4-nitrobenzaldehyde **7d** directly results in the formation of **8d**/**T1**, the more stable diastereomer that can thus be regarded as the thermodynamic product, while the reactions effected by benzaldehyde **7a**, and aldehydes **7f**,**g** carrying electron-donating group seem to proceed via intermediates **12^+^**/**R** and **13^+^**/**R** leading to **8a**,**f**,**g**/**C1**. On the other hand, regardless to the nature of the aryl group, the **Δ**G(**13^+^**−**12^+^**) values point to the preference of the primary addition **5**+**7^+^**→**13^+^** over the alternative step **5**+**7^+^**→**12^+^** constructing the appropriate rotamer and, accordingly, to the readiness of rotation **12^+^**→**13^+^**. It is also of pronounced importance that the experimentally observed substantial decrease in the propensity of **8f**,**g**/**C1** to undergo epimerization to **8f**,**g**/**T1** can be adequately interpreted in terms of the reluctance of iminium cations **14^+^f,g/E** to undergo 1,3-proton migration switching off the resonance-stabilization contributed by the electron-donating aryl substituent [cf. **Δ**G(**15^+^**−**14^+^**/**E**) values in Table 3].

Finally, the data calculated for the reaction sequence involving *O*-protonated 4-nitro-benzaldehyde **7^+^d**, suggest that the multistep annulation of **5** with electron-deficient aldehyde components proceeds via the primary formation of adducts type **13^+^/S** referring to the identical outcome of the kinetic- and thermodynamic controls.

**Figure 3:**
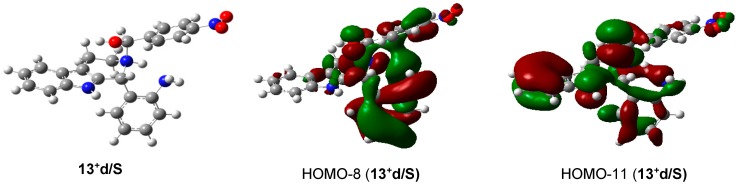
Optimized structure **13^+^d**/S and its selected MO’s involved in intramolecular interactions contributing to the stability of this cationic intermediate.

This assumption gains support from the n→π* type donor-acceptor interaction between the amino group and the C-1′ atom of the 4-nitrophenyl substituent as clearly seen from the HOMO-8 identified in the optimized structure of **13^+^d/S** (Figure 3). It must be noted here that besides this interaction the lone pair of the amino group is also incorporated in a chelate-like intramolecular H-bond with the protonated skeletal secunder amine residue (cf. HOMO-11: Figure 3).

In principle **C1**↔**T1** epimerization might also take place by the revesible deprotonation of C-6 proceeding via cations type **16^+^** (Scheme 5) enabled by the protonation of both adjacent skeletal nitrogen atoms, however this pathway must be regarded as hardly feasible as unequivocally evidenced by the values of relative energetics **Δ**G(**16^+^**−**14^+^/Z**) and **Δ**G(**16^+^**−**14^+^/Z**) even though the enhanced anion-stabilizing effect of the 4-nitrophenyl group is also reflected from the calculated data (Table 3). Finally, it seems reasonable that the pronounced readiness of pyridyl derivatives **8h,i** to undergo **C1**↔**T1** isomerization, mentioned in Section 2.1 can be attributed to a highly feasible pathway proceeding via enamine type tautomers **17h,i** (Scheme 5).

### 2.4. In vitro Cytotoxic Activity of the Novel Polycyclic β-Carbolines 8/C1, 8/T1, 9/T1, 10/C1 and 11/C1

The in vitro cytotoxicity of the polycondensed β-carboline products expressed in IC_50_ values was determined on four selected human tumour cell cultures (PANC-1: pancreatic carcinoma of ductal origin, COLO-205: colon adenocarcinoma, A-2058: malignant melanoma with high invasiveness and EBC-1: lung squamous cell carcinoma). These cell lines are dedicated models for cancerous diseases with high mortality due to the lack of efficient chemotherapy. Thus, to find new antitumor agents against our investigated tumour models is a principal goal in the field of oncology drug development. The cells were treated with the compounds at 0.1–100 µM concentration range and the cell viability was determined by real-time impedimetry analysis (adherent cells—PANC-1) or colorimetric alamarBlue assay (cultures characterizing with weak/negligible adhesion—A2058, EBC-1, and COLO-205) (see Supplementary Materials).

The data listed in Table 4 show negligible or moderate-to-significant antiproliferative effects against PANC-1-, COLO-205-, A-2058 and EBC-1 cell lines that proved to be strongly dependent on the stereostructure and on the substitution pattern of the pending aryl substituent of aldehyde-origin. Thus, although the majority of the products do not have marked effect on the investigated cell lines, several highly active pentacycles were identified as promising models with significant antiproliferative activity characterized by IC_50_ values in the low micromolar range. It is of note that while *trans* diastereomers carrying phenyl- and 3-trifluoromethylphenyl substituents (**8a**/**T1** and **8b**/**T1**) display outstanding effects on each investigated cell line even in racemic form, their ester derivatives isolated as single enantiomers (**9a**/**T1** and **9b**/**T1**) proved to be inactive in the tests. The strong impact of the substitution pattern on the cytotoxicity is spectacularly indicated by the introduction of the second trifluoromethyl group into the phenyl substituent leading to a decrease of ca. one order of magnitude in the cytotoxicity as reflected from the IC_50_ values measured for **8c**/**T1** and by the marked difference in the anticancer potential of the inactive 4-nitrophenyl derivative **8d**/**T1** and its 2-nitrophenyl-substituted counterpart **8e**/**T1**, the latter one exerting detectable effect on PANC-1 cell line. The combined effect of the substituents and the stereostructure is exemplified by comparing these results to those obtained in the tests of the highly potent racemic *cis*-pentacycle **8f**/**C1** (IC_50_ = 2.1–5.2 μM on the investigated cell lines) and its inactive *trans* counterpart **8f**/**T1** both carrying 3,4,5-trimethoxyphenyl group on ring *D*. Interestingly, an opposite order of diastereomer activity was manifested in the tests of the inactive **8g/C1**, the racemic ferrocenyl derivative isolated as single diastereomer, and the 1:3 mixture of **8g/C1** and **8g/T1** having substantial cytotoxicity on COLO-205, A-2058 and EBC-1 cell lines. (Obviously, the measured effects must be displayed by the *trans* isomer.) PANC-1 cells did not present detectable sensitivity towards this mixture. Similar degree and profile of activity were detected for 8*m/C1, the chiral carboxyferrocene-containing *cis*-diastereomer that can be regarded as a further indication of the combined effect of the substituents and the stereostructure. In accord with this view, contrary to the inactive racemic lactam **10/C1**, its ester derivative **11/C1**, obtained as a single enantiomer, displayed marked antiproliferative activity on three cell lines including PANC-1. Interestingly this model proved to be inactive against COLO-205 cells.

## 3. Materials and Methods

All fine chemicals were obtained from commercially available sources (Merck, Budapest, Hungary), Fluorochem (Headfield, U.K.), Molar Chemicals (Budapest, Hungary), (VWR, Budapest, Hungary) and used without further purification. Merck Kieselgel (230–400 mesh, 60 Å) was used for flash column chromatography. Melting points (uncorrected) were determined with a M-560 instrument (Büchi, Essen, Germany). The ^1^H- and ^13^C-NMR spectra were recorded in DMSO-*d_6_* solution in 5 mm tubes at room temperature (RT), on a Bruker DRX-500 spectrometer (Bruker Biospin, Karlsruhe, Baden Württemberg, Germany) at 500 (^1^H) and 125 (^13^C) MHz, with the deuterium signal of the solvent as the lock and TMS as internal standard (^1^H, ^13^C). The 2D-COSY, NOESY, HSQC, and HMBC spectra were obtained by using the standard Bruker pulse programs cosygpppqf (2D COSY with gradient pulses for selection and purge pulses before relaxation delay d1) for COSY, noesygpphpp (2D phase sensitive NOESY with gradient pulses in mixing time and purge pulses before relaxation delay d1 for NOESY), hsqcetgp (2D phase sensitive HSQC using Echo/Antiecho-TPPI gradient selection with decoupling during acquisition and using trim pulses in inept transfer) for HSQC and hmbcgpndqf (2D H-1/X HMBC optimized on long range couplings, no decoupling during acquisition using gradient pulses for selection for HMBC). For each compound characterized in this session the numbering of atoms used for assignment of ^1^H- and ^13^C-NMR signals correspond to IUPAC rules reflected from the given systematic names. The exact mass measurements were performed using a Q Exactive Focus orbitrap instrument (Thermo Scientific Bremen, Bremen, Germany) equipped with heated electrospray ionization source. X-ray diffraction data were collected on an XtaLab Synergy-R diffractometer (Rigaku, Neu-Isenburg, Germany) using Cu -Kα radiation (λ = 1.54184 Å). Data reduction was carried out using the software provided with the diffractometer (CrysAlisPro 1.171.40.14e, Rigaku OD, 2018)). All calculations were carried out by using the Gaussian 09 software (Gaussian Incorporation, Pittsburgh, U.S.) package [38]. The optimized structures are available from the authors.

### 3.1. (±)-1-(2-Nitrophenyl)-2,3,4,9-Tetrahydro-1H-Pyrido[3,4-b]indole (3)

Tryptamine (3.20 g, 20 mmol), 2-nitrobenzaldehyde (3,03 g, 20 mmol) and boric acid (1,24g, 20 mmol) were dissolved in acetic acid (20 mL). The resulting solution was stirred at reflux under argon for 12 h and cooled down to rt. To this cooled reaction mixture stirred intensively, saturated aqueous Na_2_CO_3_ solution (200 mL) was added in small portions. The precipitated solid was separated by filtration and recrystallized from EtOH to obtain the product as deep yellow powder. Orange solid. Yield: 4.98 g (85%). M.p. 95–98 °C. ^1^H-NMR (DMSO-*d*_6_): 10.61 (s, 1H, (N)H-9); 7.94 (dd, *J* = 7.7 Hz and 1.3 Hz 1H, H-3′); 7.54 (td, *J* = 7.8 Hz and 1.3 Hz, 1H, H-5′); 7.50 (td, *J* = 7.7 Hz and 1.3 Hz, 1H, H-4′); 7.43 (br~d, *J*~8 Hz, 1H, H-5); 7.26 (br~d, *J*~8 Hz, 1H, H-8); 7.08 (dd, *J* = 7.7 Hz and 1.3 Hz, 1H, H-6′); 7.04 (t, *J* = 7.8 Hz, 1H, H-7); 6.97 (t, *J* = 7.8 Hz, 1H, H-6); 5.66 (s, 1H, H-1); 2.93 (dt, *J =* 12.7 Hz and 5.0 Hz, 1H, H-3_b_); 2.80 (ddd, *J* = 12.7 H, 7.6 Hz and 5.0 Hz, 1H, H-3_a_); 2.73–2.62 (m, 2H, H-4_a_,4_b_). ^13^C-NMR (DMSO-*d*_6_): 149.9 (C-2′); 137.5 (C-1′); 136.4 (C-8a); 133.8 (C-9a); 133.0 (C-5′); 131.1 (C-4′); 129.0 (C-6′); 127.2 (C-4b); 124.8 (C-3′); 121.4 (C-7); 118.8 (C-6); 118.3 (C-5); 111.7 (C-8), 109.8 (C-4a); 51.4 (C-1); 40.5 (C-3); 22.5 (C-4). HRMS exact mass calcd. for C_17_H_16_N_3_O_2_ [MH]^+^, requires *m*/*z*: 294.12370 found *m*/*z*: 294.12312.

### 3.2. Methyl (1R,3S)-1-(2-Nitrophenyl)-2,3,4,9-Tetrahydro-1H-Pyrido[3,4-b]indole-3-Carboxylate (4/T)

To a cooled and stirred solution of L-tryptophan methyl ester hydrochloride (5.09 g, 20 mmol) in water (50 mL) a solution of KOH (1.12 g, 20 mmol in 10 mL of water) was added in small portions and the resulting mixture was extracted with CH_2_Cl_2_ (4 × 15 mL). The organic phase was dried over Na_2_SO_4_ and evaporated to dryness. The oily residue, 2-nitrobenzaldehyde (3.03 g, 20 mmol) and boric acid (1.24 g, 20 mmol) were dissolved in acetic acid and (20 mL). The solution was stirred at reflux under argon for 12 h and cooled down to rt. To the stirred reaction mixture saturated Na_2_CO_3_ solution (200 mL) was added in small portions. The precipitated solid containing ca. 1:2 mixture of **4/C** and **4/T** was subjected to flash chromatography on silica using CH_2_Cl_2_-MeOH (80:1) as eluent (R_f_ = 0.20 for **4**/**T** and 0.23 for **4**/**C** as detected by TLC). (No detectable separation could be observed when more polar solvents, e.g., CH_2_Cl_2_-MeOH (from 50:1 to 10:1) were used as eluent.) The complete separation of the eluting fractions could not be achieved, so the non-overlapping upper part of the second band was collected and evaporated to dryness. The oily residue was crystallized with cold MeOH to obtain the product as a yellow powder. Orange solid. Yield: 2.95 g (42%). M.p. 105–107 °C. ^1^H-NMR (DMSO-*d*_6_): 10.66 (s, 1H, (N)H-9); 7.93 (dd, *J* = 7.7 Hz and 1.3 Hz 1H, H-3′); 7.52 (br~t, *J*~8 Hz, 1H, H-5′); 7.48 (td, *J* = 7.7 Hz and 1.3 Hz, 1H, H-4′); 7.44 (br~d, *J*~8 Hz, 1H, H-5); 7.22 (br~d, *J*~8 Hz, 1H, H-8); 7.02 (t, *J* = 7.8 Hz, 1H, H-7); 6.97–6.93 (m, 2H, H-6,6′); 5.81 (s, 1H, H-1); 3.63 (ddd, *J* = 8.4 Hz, 6.8 Hz and 5.0 Hz, 1H, H-3_a_); 3.57 (s, 3H, 3-CO_2_CH_3_); 3.03 (dd, *J* = 15.0 Hz and 5.0 Hz, 1H, H-4_a_); 2.82 (ddd, *J* = 15.0 Hz, 8.4 Hz and 1.2 Hz, 1H, H-4_b_). ^13^C-NMR (DMSO-*d*_6_): 172.8 (3-CO_2_CH_3_);149.9 (C-2′); 136.4 (C-8a); 135.2 (C-9a); 133.3 (C-5′); 131.1 (C-4′); 129.3 (C-6′); 128.0 (C-1′); 127.1 (C-4b); 125.1 (C-3′); 121.6 (C-7); 119.1 (C-6); 118.2 (C-5); 111.7 (C-8), 107.8 (C-4a); 52.2 (3-CO_2_CH_3_); 52.1 (C-3); 49.8 (C-1); 25.1 (C-4). HRMS exact mass calcd. for C_19_H_18_N_3_O_4_ [MH]^+^, requires *m*/*z*: 352.12918, found *m*/*z*: 352.12851.

### 3.3. Hydrogenation of 1-(2-Nitrophenyl)-2,3,4,9-Tetrahydro-1H-Pyrido[3,4-b]indoles 3 and 4/T

The corresponding 2-nitrophenyl derivative (10 mmol) was dissolved in MeOH (20 mL) In the presence of 10% Pd/C (1.21 g) the reaction mixture was stirred under H_2_ (1 atm, balloon) for 6 h. The catalyst was removed by filtration over celite and the filtrate was concentrated in vacuo. The reulting solid was triturated with cold MeOH and filtered off to obtain the product as colorless crystals.

#### 3.3.1. (±)-1-(2-Aminophenyl)-2,3,4,9-Tetrahydro-1H-Pyrido[3,4-b]indole (5)

Colourless solid. Yield: 2.16 g (82%). M.p. 123–126 °C.^1^H-NMR (DMSO-*d*_6_): 10.61 (s, 1H, (N)H-9); 7.41 (d, *J* = 7.8 Hz, 1H, H-5); 7.25 (d, *J* = 7.8 Hz, 1H, H-8); 7.04–6.98 (m, 2H, H-3,7); 6.96 (t, *J* = 7.8 Hz, 1H, H-6); 6.68–6.65 (m, 2H, H-1,4); 6.49 (t, *J* = 7.7 Hz, 1H, H-2′); 5.25 (br s, 2H, 2′-NH_2_); 5.21 (s, 1H, H-1); 3.29 (br~s, 1H, (N)H-2); 2.95 (br s, 2H, H-3_a_3_b_); 2.69 (br s, 2H, H-4_a_4_b_); ^13^C-NMR (DMSO-*d*_6_): 147.8 (C-2′); 136.3 (C-8a); 135.3 (C-9a); 129.9 (C-6′); 128.4 (C-4′); 127.4 (C-4b); 125.9 (C-1′); 120.8 (C-7); 118.6 (C-6); 117.9 (C-5); 116.2 (C-5′); 115.8 (C-3′); 111.5 (C-8), 108.7 (C-4a); 51.7 (C-1); 41.5 (C-3); 22.8 (C-4). HRMS exact mass calcd. for C_17_H_18_N_3_ [MH]^+^, requires *m*/*z*: 264.14952 found *m*/*z*: 264.14896.

#### 3.3.2. Methyl (1R,3S)-1-(2-Aminophenyl)-2,3,4,9-Tetrahydro-1H-Pyrido[3,4-b]indole-3-Carboxylate (6/T)

Colourless solid. Yield: 2.41 g (75%). M.p 110–113 °C. ^1^H-NMR (DMSO-*d*_6_): 10.69 (s, 1H, (N)H-9); 7.41 (d, *J* = 7.8 Hz, 1H, H-5); 7.21 (d, *J* = 7.8 Hz, 1H, H-8); 7.00 (t, *J* = 7.8 Hz, 1H, H-7); 6.95 (t, *J* = 7.5 Hz, 1H, H-4′); 6.93 (t, *J* = 7.88 Hz, 1H, H-6); 6.65 (d, *J* = 7.6 Hz, 1H, H-3′); 6.43–6.38 (m, 2H, H-5′,6′); 5.28 (s, 1H, H-1); 5.22 (br s, 2H, 2′-NH_2_); 3.60 (s, 3H, 3-CO_2_CH_3_); 3.57 (br ~s, 1H, H-3); 3.09 (br s, 1H, (N)H-2); 2.98 (dd, *J =* 15.1 Hz and 5.0 Hz, 1H, H-4_a_); 2.79 (dd, *J =* 15.1 Hz and 9.1 Hz, 1H, H-4_b_). ^13^C-NMR (DMSO-*d*_6_):174.2 (3-CO_2_CH_3_); 147.7 (C-2′); 136.4 (C-8a); 134.5 (C-9a); 129.4 (C-6′); 128.5 (C-4′); 127.1 (C-4b); 125.9 (C-1′); 121.3 (C-7), 118.8 (C-6); 118.0 (C-5); 116.2 (C-5′); 115.9 (C-3′); 111.5 (C-8); 107.6 (C-4a); 52.3 (C-3); 52.1 (3-CO_2_CH_3_); 51.7 (C-1); 25.7 (C-4). HRMS exact mass calcd. for C_19_H_20_N_3_O_2_ [MH]^+^, requires *m*/*z*: 322.15500 found *m*/*z*: 322.15441.

### 3.4. Cyclization Reactions of 1-(2-Aminophenyl)-2,3,4,9-Tetrahydro-1H-Pyrido[3,4-b]indoles 5 and 6/T

The corresponding 2-aminophenyl derivative (2 mmol) and the aldehyde component (1.1 mmol) were stirred and heated under argon at reflux temperature in a mixture of MeOH (10 mL) and AcOH (2 mL) for 0.5-16 h and cooled down to rt. (The exact reaction times are given along with the isolated yields of the products as presented in the sections below describing product characterization.) To this reaction mixture, cooled with ice-water, saturated aqueous Na_2_CO_3_ solution (15 mL) was added in small portions. (The reaction mixtures obatined by the cyclizations involving **7m** as aldehyde component were diluted with 10 mL of water and carefully neutralized by the addition of solid NaHCO_3_ in small portions.) The precipitated solid was separated by filtration, triturated with a cold 2:5 mixture of MeOH and water (ca. 15 mL), filtered off again and dried in dessicator over KOH pellets. Analytical samples were recrystallized from MeOH. (In order to remove the traces of tarry contaminations formed in uncontrolled processes, the crude products of the cyclization reactions with ferrocene reagents **7l** and **7m** were dissolved in 20 mL of CH_2_Cl_2_ and passed through a celite pad.) The solution was evaporated to obtain an oily or solid residue which was triturated with a cold 1:5 mixture of MeOH and water (ca. 10 mL). The purified product was filtered off and dried in dessicator over KOH pellets.

#### 3.4.1. (6R*,14bS*)-6-Phenyl-5,6,8,9,14,14b-Hexahydroindolo[2′,3′:3,4]pyrido[1,2-c]quinazoline (8a/T1)

Colourless solid. Yield: 0.260 g (37%) with 0.5 h reaction time; 0.556 g (77%) with 12 h reaction time. M.p. 153–156 °C. ^1^H-NMR (DMSO-*d*_6_): 10.99 (s, 1H, (N)H-14); 7.46 (d, *J* = 7.4 Hz, 2H, H-2′,6′); 7.34 (t, *J* = 7.4 Hz, 2H, H-3′,5′); 7.26 (t, *J* = 7.4 Hz, 1H, H-4′); 7.39 (d, *J* = 7.8 Hz, 1H, H-10); 7.30 (d, *J* = 7.8 Hz, 1H, H-13); 7.03 (t, *J* = 7.8 Hz, 1H, H-12); 7.00 (d, *J* = 7.5 Hz, 1H, H-1); 6.98 (t, *J* = 7.5 Hz, 1H, H-3); 6.95 (t, *J* = 7.8 Hz, 1H, H-11); 6.78 (d, *J* = 3.4 Hz, 1H, H-5); 6.71 (br~d, *J*~8 Hz, 1H, H-4); 6.45 (t, *J* = 7.6 Hz, 1H, H-2); 5.28 (d, *J* = 3.4 Hz, 1H, H-6); 4.66 (br s, 1H, H-14b); 3.19 (dd, *J* = 10.9 Hz and 5.9 Hz, 1H, H-8_b_); 2.97 (td, *J* = 10.9 Hz and 3.6 Hz, 1H, H-8_a_); 2.89 (ddd, *J* = 15.1 Hz, 10.9 Hz and 5.9 Hz, 1H, H-9_b_); 2.68 (dd, *J* = 14.1 Hz and 3.6 Hz, 1H, H-9_a_). ^13^C-NMR (DMSO-*d*_6_): 144.0 (C-1′); 142.0 (C-4a); 136.3 (C-13a); 134.9 (C-14a); 128.7 (C-3′,5′); 128.0 (C-1); 127.9 (C-3); 127.8 (C-4′); 127.5 (C-2′,6′); 127.0 (C-9b); 121.1 (C-12), 119.3 (C-14c); 118.8 (C-11); 118.2 (C-10); 115.9 (C-2); 113.6 (C-4); 111.5 (C-13); 106.5 (C-9a); 72.6 (C-6); 49.9 (C-14b); 46.6 (C-8); 23.2 (C-9). HRMS exact mass calcd. for C_24_H_22_N_3_ [MH]^+^, requires *m*/*z*: 352.18082, found *m*/*z*: 352.18022.

#### 3.4.2. (6R*,14bS*)-6-(3-Trifluoromethylphenyl)-5,6,8,9,14,14b-Hexahydroindolo[2′,3′:3,4]pyrido[1,2-c]quinazoline (8b/T1)

Colourless solid. Yield: 0.630 g (75%) with 1 h reaction time. 0.604 g (72%) with 12 h reaction time. M.p. 253–255 °C. ^1^H-NMR (DMSO-*d*_6_): 10.96 (s, 1H, (N)H-14); 7.75–7.71 (overlapping m’s, 2H, H-2′,6′); 7.61 (d, *J* = 7.6 Hz, 1H, H-4′); 7.56 (t, *J* = 7.6 Hz, 1H, H-5′); 7.36 (d, *J* = 7.8 Hz, 1H, H-10); 7.26 (d, *J* = 7.8 Hz, 1H, H-13); 7.01 (t, *J* = 7.8 Hz, 1H, H-12); 6.98–6.94 (overlapping m’s, 2H, H-1,3); 6.92 (t, *J* = 7.8 Hz, 1H, H-11); 6.86 (d, *J* = 3.7 Hz, 1H, H-5); 6.70 (d, *J* = 7.6 Hz, 1H, H-4); 6.43 (td, *J* = 7.6 Hz and 1.2 Hz, 1H, H-2); 5.35 (d, *J* = 3.7 Hz, 1H, H-6); 4.56 (br s, 1H, H-14b); 3.18 (m, 1H, H-8_b_); 2.96–2.87 (m, 3H H-8_a_,9_a_,9_b_). ^13^C-NMR (DMSO-*d*_6_): 145.6 (C-1′); 141.4 (C-4a); 136.3 (C-13a); 134.6 (C-14a); 131.9 (C-6′); 130.0 (C-5′); 129.5 (qa, *J* = 30.8 Hz, C-3′); 128.1 (C-1); 128.0 (C-3); 127.1 (qa, *J* = 272.5 Hz, CF_3_); 126.9 (C-9b); 124.7 (C-4′); 124.0 (C-2′); 121.2 (C-12), 119.3 (C-14c); 118.9 (C-11); 118.3 (C-10); 116.3 (C-2); 113.8 (C-4); 111.5 (C-13); 106.6 (C-9a); 72.2 (C-6); 49.9 (C-14b); 46.5 (C-8); 22.1 (C-9 HRMS exact mass calcd. for C_25_H_21_F_3_N_3_ [MH]^+^, requires *m*/*z*: 420.16766 found *m*/*z*: 420.16755.

#### 3.4.3. (6R*,14bS*)-6-[3,5-Bis(trifluoromethyl)phenyl]-5,6,8,9,14,14b-Hexahydroindolo[2′,3′:3,4]pyrido-[1,2-c]quinazoline (8c/T1)

Colourless solid. Yield: 0.770 g (79%) with 1 h reaction time. 0.741 g (76%) with 12 h reaction time. M.p. 247–250 °C. ^1^H-NMR (DMSO-*d*_6_): 10.96 (s, 1H, (N)H-14); 8.04 (br s, 2H, H-2′,6′); 8.02 (br s, 1H, H-4′); 7.36 (d, *J* = 7.8 Hz, 1H, H-10); 7.28 (d, *J* = 7.8 Hz, 1H, H-13); 7.02 (br~t, *J*~8 Hz, 1H, H-12); 6.99–6.95 (overlapping m’s, 3H, H-1,3,5); 6.93 (br~t, *J*~8 Hz, 1H, H-11); 6.74 (br~d, *J*~8 Hz, 1H, H-4); 6.46 (td, *J* = 7.8 Hz and 1.2 Hz, 1H, H-2); 5.48 (d, *J* = 3.2 Hz, 1H, H-6); 4.54 (br s, 1H, H-14b); 3.21 (m, 1H, H-8_b_); 2.96–2.87 (m, 3H H-8_a_,9_a_,9_b_). ^13^C-NMR (DMSO-*d*_6_): 147.9 (C-1′); 141.0 (C-4a); 136.3 (C-13a); 134.2 (C-14a); 130.8 (qa, *J* = 32.6 Hz, C-3′,5′); 128.4 (br, C-2′,6′); 128.3 (C-3); 128.1 (C-1); 126.9 (C-9b); 123. 8 (qa, *J* = 273.6 Hz, 3′,5′-CF_3_); 121.9 (br, C-4′); 121.2 (C-12), 119.3 (C-14c); 118.9 (C-11); 118.3 (C-10); 116.7 (C-2); 114.0 (C-4); 111.5 (C-13); 106.6 (C-9a); 71.7 (C-6); 49.8 (C-14b); 46.5 (C-8); 22.0 (C-9). HRMS exact mass calcd. for C_26_H_20_F_6_N_3_ [MH]^+^, requires *m*/*z*: 488.15559, found *m*/*z*: 488.15512.

#### 3.4.4. (6R*,14bS*)-6-(4-Nitromethylphenyl)-5,6,8,9,14,14b-Hexahydroindolo[2′,3′:3,4]pyrido[1,2-c]-quinazoline (8d/T1)

Light orange solid. Yield: 0.587 g (74%) with 1 h reaction time. 0.618 g (78%) with 12 h reaction time. M.p. 292–295 °C. ^1^H-NMR (DMSO-*d*_6_): 10.94 (s, 1H, (N)H-14); 8.19 (d, *J* = 8.8 Hz, 2H, H-3′,5′); 7.69 (d, *J* = 8.8 Hz, 2H, H-2′,6′); 7.36 (d, *J* = 7.8 Hz, 1H, H-10); 7.26 (d, *J* = 7.8 Hz, 1H, H-13); 7.01 (t, *J* = 7.8 Hz, 1H, H-12); 6.98–6.94 (overlapping m’s, 2H, H-1,3); 6.92 (t, *J* = 7.8 Hz, 1H, H-11); 6.88 (d, *J* = 3.9 Hz, 1H, H-5); 6.71 (dd, *J* = 7.6 Hz and 1.2 Hz, 1H, H-4); 6.43 (td, *J* = 7.6 Hz and 1.2 Hz, 1H, H-2); 5.38 (d, *J* = 3.7 Hz, 1H, H-6); 4.54 (br s, 1H, H-14b); 3.20 (dd, *J* = 10.2 Hz and 4.6 Hz, 1H, H-8_b_); 2.93 (td, *J* = 10.2 Hz and 4.0 Hz, 1H, H-8_a_); 2.97–2.84 (m, 2H H-9_a_,9_b_). ^13^C-NMR (DMSO-*d*_6_): 151.7 (C-1′); 147.4 (C-4′); 141.3 (C-4a); 136.5 (C-13a); 134.5 (C-14a); 129.0 (C-2′,6′); 128.1 (C-3); 128.0 (C-1); 127.0 (C-9b); 124.1 (C-3′,5′); 121.2 (C-12), 119.3 (C-14c); 119.0 (C-11); 118.3 (C-10); 116.4 (C-2); 113.9 (C-4); 111.5 (C-13); 106.6 (C-9a); 72.4 (C-6); 60.2 (C-14b); 46.7 (C-8); 22.3 (C-9). HRMS exact mass calcd. for C_24_H_21_N_4_O_2_ [MH]^+^, requires *m*/*z*: 397.16600, found *m*/*z*: 397.16542.

#### 3.4.5. (6R*,14bS*)-6-(2-Nitrophenyl)-5,6,8,9,14,14b-Hexahydroindolo[2′,3′:3,4]pyrido[1,2-c]-quinazoline (8e/T1)

Yellow solid. Yield: 0.634 g (80%) with 1 h reaction time. 0.595 g (75%) with 12 h reaction time. M.p. 275–279 °C. ^1^H-NMR (DMSO-*d*_6_): 10.97 (s, 1H, (N)H-14); 7.75 (br~d, *J*~8 Hz, 1H, H-3′); 7.62 (t, *J* = 7.7 Hz, 1H, H-5′); 7.57 (dd, *J* = 7.7 Hz and 1.7 Hz, 1H, H-6′); 7.48 (dt, *J* = 7.7 Hz and 1.7 Hz, 1H, H-4′); 7.33 (d, *J* = 7.8 Hz, 1H, H-10); 7.25 (d, *J* = 7.8 Hz, 1H, H-13); 7.01 (t, *J* = 7.8 Hz, 1H, H-12); 6.98 (t, *J* = 7.5 Hz, 1H, H-3); 6.94 (d, *J* = 7.5 Hz, 1H, H-1); 6.91 (t, *J* = 7.8 Hz,1H, H-11); 6.85 (d, *J* = 4.1 Hz, 1H, H-5); 6.73 (d, *J* = 7.5 Hz, 1H, H-4); 6.43 (t, *J* = 7.5 Hz, 1H, H-2); 5.84 (d, *J* = 4.1 Hz, 1H, H-6), 4.31 (br s, 1H, H-14b); 3.21 (m, 1H, H-8_b_); 2.96–2.87 (m, 3H H-8_a_,9_a_,9_b_). ^13^C-NMR (DMSO-*d*_6_): 149.4 (C-2′); 141.4 (C-4a); 136.9 (C-1′); 136.3 (C-13a); 134.0 (C-14a); 132.5 (C-5′); 129.4 (C-4′); 129.3 (C-6′); 128.2 (C-3); 127.8 (C-1); 126.9 (C-9b); 125.2 (C-3′); 121.2 (C-12); 119.1 (C-14c); 118.9 (C-11); 118.2 (C-10); 116.6 (C-2); 113.9 (C-4); 111.5 (C-13); 106.6 (C-9a); 69.5 (C-6); 49.7 (C-14b); 46.9 (C-8); 21.9 (C-9). HRMS exact mass calcd. for C_24_H_21_N_4_O_2_ [MH]^+^, requires *m*/*z*: 397.16600, found *m*/*z*: 397.16590.

#### 3.4.6. (6R*,14bR*)-6-(3,4,5-Trimethoxyphenyl)-5,6,8,9,14,14b-Hexahydroindolo[2′,3′:3,4]pyrido[1,2-c]quinazoline (8f/C1)

Colourless solid. Yield: 0.556 g (63%) with 0.5 h reaction time. M.p. 235–238 °C. ^1^H-NMR (DMSO-*d*_6_): 11.27 (s, 1H, (N)H-14); 7.35 (d, *J* = 7.8 Hz, 1H, H-13); 7.31 (d, *J* = 7.8 Hz, 1H, H-10); 7.20 (d, *J* = 7.9 Hz, 1H, H-1); 7.02 (t, *J* = 7.8 Hz, 1H, H-12); 6.95 (t, *J* = 7.9 Hz, 1H, H-3); 6.93 (t, *J* = 7.8 Hz, 1H, H-11); 6.91 (s, 2H, H-2′,6′); 6.73 (d, *J* = 7.9 Hz, 1H, H-4); 6.54 (t, *J* = 7.9 Hz, 1H, H-2); 6.28 (br s, 1H, H-5); 5.60 (two coalesced lines, 2H, H-6,14b); 3.66 (s, 6H, 3′,5′-OCH_3_); 361 (s, 3H, 4′-OCH_3_); 2.73 (m, 1H, H-8_b_); 2.60–2.47 (overlapping m’s, 3H, H-8_a_9_a_,9_b_). ^13^C-NMR (DMSO-*d*_6_): 153.2 (C-3′,5′); 144.0 (C-4a); 139.4 (C-1′); 137.2 (C-4′); 136.6 (C-13a); 134.9 (C-14a); 127.9 (C-1); 127.5 (C-3); 126.8 (C-9b); 121.4 (C-14c); 121.1 (C-12), 118.8 (C-11); 118.2 (C-10); 117.6 (C-2); 115.6 (C-4); 111.5 (C-13); 107.3 (C-9a); 105.0 (C-2′,6′); 73.2 (C-6); 60.5 (4′-OCH_3_); 56.4 (3′,5′-OCH_3_); 57.5 (C-14b); 38.0 (C-8); 21.9 (C-9). HRMS exact mass calcd. for C_27_H_28_N_3_O_3_ [MH]^+^, requires *m*/*z*: 442.212520, found *m*/*z*: 442.21261.

#### 3.4.7. (6R*,14bS*)-6-(3,4,5-Trimethoxyphenyl)-5,6,8,9,14,14b-Hexahydroindolo[2′,3′:3,4]pyrido[1,2-c]quinazoline (8f/T1)

Colourless solid. Yield: present in ca. 80% of the isomer mixture **8f**/**T1** and **8f**/**C1** isolated after 8 h reaction time with weight of 0.662 g (75%). ^1^H-NMR (DMSO-*d*_6_): 10.91 (s, 1H, (N)H-14), 7.35 (d, *J* = 7.8 Hz, 1H, H-10); 7.27 (d, *J* = 7.8 Hz, 1H, H-13); 7.03–6.98 (overlapping m’s, 2H, H-1,12); 6.94 (t, *J* = 7.9 Hz, 1H, H-3); 6.91 (t, *J* = 7.8 Hz, 1H, H-11); 6.74 (s, 2H, H-2′,6′); 6.68 (d, *J* = 3.1 Hz, 1H, H-5); 6.65 (d, *J* = 7.9 Hz, 1H, H-4); 6.42 (t, *J* = 7.9 Hz, 1H, H-2); 5.14 (d, *J* = 3.1 Hz, 1H, H-6), 4.74 (br s, 1H, H-14b); 3.63 (s, 6H, 3′,5′-OCH_3_); 3.59 (s, 3H, 4′-OCH_3_); 3.12 (ddd, *J* = 10.6 Hz, 5.6 Hz and 2.0 Hz, 1H, H-8_b_); 2.92 (td, *J* = 10.6 Hz, 3.9 Hz, 1H, H-8_a_); 2.82 (m, 1H, H-9_b_); 2.64 (br~d, *J*~15 Hz, 1H, H-9_a_). ^13^C-NMR (DMSO-*d*_6_): 153.3 (C-3′,5′); 141.9 (C-4a); 139.7 (C-1′); 137.3 (C-4′); 136.5 (C-13a); 135.0 (C-14a); 128.1 (C-3); 128.0 (C-1); 127.2 (C-9b); 121.6 (C-12), 119.4 (C-14c); 119.0 (C-11); 118.4 (C-10); 116.0 (C-2); 113.8 (C-4); 111.4 (C-13); 106.5 (C-9a); 72.4 (C-6); 60.7 (4′-OCH_3_); 56.5 (3′,5′-OCH_3_); 50.5 (C-14b); 46.7 (C-8); 21.8 (C-9).

#### 3.4.8. (6R*,14bR*)-6-Ferrocenyl-5,6,8,9,14,14b-Hexahydroindolo[2′,3′:3,4]pyrido[1,2-c]quinazoline (8g/C1)

Yellow solid. Yield: 0.358 g (39%) with 0.5 h reaction time. M.p. 211–215 °C (decomp.). ^1^H-NMR (DMSO-*d*_6_): 11.23 (s, 1H, (N)H-14); 7.33 (d, *J* = 7.8 Hz, 1H, H-13); 7.27 (d, *J* = 7.8 Hz, 1H, H-10); 7.15 (d, *J* = 7.6 Hz, 1H, H-1); 7.00 (t, *J* = 7.8 Hz, 1H, H-12); 6.94 (t, *J* = 7.6 Hz, 1H, H-3); 6.89 (t, *J* = 7.8 Hz, 1H, H-1,11); 6.81 (d, *J* = 7.6 Hz, 1H, H-4); 6.49 (t, *J* = 7.6 Hz, 1H, H-2); 5.60 (br s, 1H, H-6); 5.53 (br s, 1H, H-5); 5.49 (br s, 1H, H-14b); 4.55 (br s, 1H, H-2′); 4.34 (br s, 1H, H-5′); 4.22 (s, 5H, η^5^-C_5_H_5_); 4.19 (br s, 2H, H-3′,4′); 2.80 (m, 1H, H-8_b_); 2.48-2.43 (overlapping m’s, 3H, H-8_b_,9_a_,9_b_). ^13^C-NMR (DMSO-*d*_6_): 143.8 (C-4a); 136.5 (C-13a); 134.9 (C-14a); 127.9 (C-1); 127.4 (C-3); 126.7 (C-9b); 120.9 (C-12); 120.8 (C-14c); 118.6 (C-11); 118.3 (C-10); 115.0 (C-2); 113.5 (C-4); 111.4 (C-13); 107.0 (C-9a); 86.7 (C-1′); 71.0 (C-6); 69.2 (η^5^-C_5_H_5_); 68.4 (C-5′); 67.8 (two coalesced lines, C-3′,4′); 67.0 (C-2′); 57.2 (C-14b); 37.4 (C-8); 21.3 (C-9). HRMS exact mass calcd. for C_28_H_26_FeN_3_ [MH]^+^, requires *m*/*z*: 460.14706, found *m*/*z*: 460.14641.

#### 3.4.9. (6R*,14bS*)-6-Ferrocenyl-5,6,8,9,14,14b-Hexahydroindolo[2′,3′:3,4]pyrido[1,2-c]quinazoline (8g/T1)

Yield: present in ca. 70% of the isomer mixture **8g**/**T1** and **8g**/**C1** isolated after 8 h reaction time with weight of 0.477 g (52%). ^1^H-NMR (DMSO-*d*_6_): 11.02 (s, 1H, (N)H-14); 7.31 (d, *J* = 7.8 Hz, 1H, H-10); 7.24 (d, *J* = 7.8 Hz, 1H, H-13); 6.99 (t, *J* = 7.8 Hz, 1H, H-12); 6.95 (d, *J* = 7.6 Hz, 1H, H-1); 6.91 (d, *J* = 7.6 Hz, 1H, H-3); 6.88 (t, *J* = 7.8 Hz, 1H, H-1,11); 6.67 (d, *J* = 7.6 Hz, 1H, H-4); 6.45 (d, *J* = 3.4 Hz, 1H, H-5); 6.36 (t, *J* = 7.6 Hz, 1H, H-2); 5.18 (d, *J* = 3.4 Hz, 1H, H-6); 4.75 (br s, 1H, H-14b); 4.63 (t, *J* = 2.2 Hz, 1H, H-2′); 4.08 (t, *J* = 2.2 Hz, 1H, H-5′); 4.00 (br s, 2H, H-3′,4′); 4.21 (s, 5H, η^5^-C_5_H_5_); 3.01 (m, 1H, H-8_b_); 2.80–2.77 (overlapping m’s, 2H, H-8_b_,9_a_); 2.56 (m, 1H, H-9_b_). ^13^C-NMR (DMSO-*d*_6_): 142.2 (C-4a); 136.3 (C-13a); 135.0 (C-14a); 127.7 (C-1); 127.5 (C-3); 126.9 (C-9b); 120.9 (C-12); 118.9 (C-14c); 118.6 (C-11); 118.3 (C-10); 115.6 (C-2); 113.3 (C-4); 111.2 (C-13); 106.5 (C-9a); 91.5 (C-1′); 73.5 (C-2′); 70.9 (C-6); 70.1 (C-5′); 69.3 (η^5^-C_5_H_5_); 67.7 (two coalesced lines, C-3′,4′);49.5 (C-14b); 46.2 (C-8); 21.6 (C-9).

#### 3.4.10. (6R*,14bR*)-6-(Pyridine-2-yl)-5,6,8,9,14,14b-Hexahydroindolo[2′,3′:3,4]pyrido[1,2-c]quinazoline (8h/C1)

Light yellow solid. Yield: present in ca. 60% of the isomer mixture **8h**/**T1** and **8h**/**C1** isolated after 0.5 reaction time with weight of 0.508 g (72%). ^1^H-NMR (DMSO-*d*_6_): 11.36 (s, 1H, (N)H-14); 8.67 (dd, *J* = 4.5 Hz and 1.2 Hz, 1H, H-6′); 7.92 (td, *J* = 8.2 Hz and 1.2 Hz, 1H, H-4′); 7.73 (d, *J* = 8.2 Hz, 1H, H-3′); 7.43 (dd, *J* = 8.2 Hz and 4.5 Hz, 1H, H-5′); 7.41 (d, *J* = 7.8 Hz, 1H, H-13); 7.36 (d, *J* = 7.8 Hz, 1H, H-10); 7.25 (d, *J* = 7.6 Hz, 1H, H-1); 7.08 (t, *J* = 7.8 Hz, 1H, H-12); 7.03-6.94 (m, 2H, H-3,11); 6.86 (d, *J* = 7.8 Hz, 1H, H-4); 6.57 (t, *J* = 7.5 Hz, 1H, H-2); 6.38 (br s, 1H, H-5); 5.77 (br s, 1H, H-6), 5.71(br s, 1H, H-14b); 2.63 (br ~d, *J*~10 Hz, 1H, H-8_b_); 2.56–2.51 (m, 3H, H-8_a_,9_a_,9_b_). ^13^C-NMR (DMSO-*d*_6_): 157.1 (C-2′); 146.9 (C-6′); 143.3 (C-4a); 137.5 (C-4′); 136.3 (C-13a); 135.2 (C-14a); 127.9 (C-1); 127.7 (C-3); 126.9 (C-9b); 123.6 (C-5′); 122.4 (C-3′); 121.2 (C-12), 120.9 (C-14c); 118.8 (C-11); 118.2 (C-10); 117.3 (C-2); 113.6 (C-4); 111.6 (C-13); 107.0 (C-9a); 73.1 (C-6); 57.7 (C-14b); 38.7 (C-8); 21.9 (C-9). HRMS exact mass calcd. for C_23_H_21_N_4_ [MH]^+^, requires *m*/*z*: 353.17571, found *m*/*z*: 353.17607.

#### 3.4.11. (6R*,14bS*)-6-(Pyridine-2-yl)-5,6,8,9,14,14b-Hexahydroindolo[2′,3′:3,4]pyrido[1,2-c]-Quinazoline (8h/T1)

Yield: present in ca. 40% of the isomer mixture **8h**/**T1** and **8h**/**C1** isolated after 0.5 reaction time with weight of 0.508 g (72%). ^1^H-NMR (DMSO-*d*_6_): 11.00 (s, 1H, (N)H-14); 8.54 (dd, *J* = 4.6 Hz and 1.2 Hz, 1H, H-6′); 7.79 (dd, *J* = 8.3 Hz and 1.2 Hz, 1H, H-4′); 7.49 (d, *J* = 8.2 Hz, 1H, H-4′); 7.36 (d, *J* = 7.8 Hz, 1H, H-10); 7.32 (d, *J* = 7.8 Hz, 1H, H-13); 7.29 (dd, *J* = 8.3 Hz and 4.6 Hz, 1H, H-5′); 7.05 (dd, *J* = 7.6 Hz and 1.2 Hz, 1H, H-1); 7.03 (t, *J* = 7.8 Hz, 1H, H-12); 7.01-6.92 (m, 2H, H-3,11); 6.71 (d, *J* = 3.6 Hz, 1H, H-5); 6.68 (d, *J* = 7.9 Hz, 1H, H-4); 6.47 (t, *J* = 7.5 Hz, 1H, H-2); 5.28 (d, *J* = 3.6 Hz, 1H, H-6), 4.79 (br s, 1H, H-14b); 3.20 (m, 1H, H-8_b_); 2.99 (td, *J* = 12.4 Hz and 4.0 Hz, 1H, H-8_a_); 2.63 (m, 1H, H-9_b_); 2.70 (dd, *J* = 15.5 Hz and 4.0 Hz, 1H, H-9_a_). ^13^C-NMR (DMSO-*d*_6_): 162.0 (C-2′); 147.2 (C-6′); 142.2 (C-4a); 137.1 (C-4′); 135.8 (C-13a); 135.2 (C-14a); 128.0 (C-3); 127.8 (C-1); 126.9 (C-9b); 123.2 (C-5′); 122.0 (C-3′); 121.2 (C-12), 119.3 (C-14c); 118.8 (C-11); 118.3 (C-10); 115.9 (C-2); 113.7 (C-4); 111.6 (C-13); 106.9 (C-9a); 74.3 (C-6); 50.3 (C-14b); 47.2 (C-8); 22.0 (C-9).

#### 3.4.12. (6R*,14bR*)-6-(3-Fluoropyridine-2-yl)-5,6,8,9,14,14b-Hexahydroindolo[2′,3′:3,4]pyrido[1,2-c]quinazoline (8i/C1)

Colourless solid. Yield: present in ca. 50% of the isomer mixture **8i**/**T1** and **8i**/**C1** isolated after 0.5 reaction time with weight of 0.593 g (80%). ^1^H-NMR (DMSO-*d*_6_): 11.37 (s, 1H, (N)H-14); 8.56 (br~d, *J*~5 Hz, 1H, H-6′); 7.83 (dd, *J* = 10.2 Hz and 8.2 Hz, 1H, H-4′); 7.60 (m, 1H, H-5′); 7.40 (d, *J* = 7.8 Hz, 1H, H-13); 7.36 (d, *J* = 7.8 Hz, 1H, H-10); 7.24 (d, *J* = 7.6 Hz, 1H, H-1); 7.04–6.94 (m, 4H, H-1,3,11,12); 6.85 (d, *J* = 7.9 Hz, 1H, H-4); 6.59 (t, *J* = 7.6 Hz, 1H, H-2); 6.21 (d, *J* = 3.4 Hz, 1H, H-5); 6.00 (d, *J* = 3.4 Hz, 1H, H-6), 5.23 (br s, 1H, H-14b); ~2.5 (overlapped by the CD_2_H solvent signal, H-8_b,_9_a,_9_b_); 3.01 (td, *J* = 11.8 Hz and 4.2 Hz, 1H, H-8_a_); 2.84 (ddd, *J* = 15.5 Hz 11.8 Hz and 6.3 Hz, 1H, H-9_b_); 2.66 (dd, *J* = 15.5 Hz and 4.2 Hz, 1H, H-9_a_). ^13^C-NMR (DMSO-*d*_6_): 153.5 (d, *J* = 258.2 Hz, C-3′); 151.2 (d, *J* = 11.6 Hz, C-2′); 144.2 (d, *J* = 5.0 Hz, C-6′); 142.9 (C-4a); 136.5 (C-13a); 134.7 (C-14a); 128.1 and 128.0 (C-1 and C-3); 126.8 (C-9b); 126.1 (d, *J* = 4.5 Hz, C-5′); 124.8 (d, *J* = 20.8 Hz, C-5′); 121.3 (C-12); 120.5 (C-14c); 118.8 (C-11); 118.2 (C-10); 117.8 (C-2); 116.1 (C-4); 111.5 (C-13); 106.8 (C-9a); 69.0 (C-6); 57.5 (C-14b); 38.3 (C-8); 21.7 (C-9). HRMS exact mass calcd. for C_23_H_20_FN_4_ [MH]^+^, requires *m*/*z*: 371.16665, found *m*/*z*: 371.16614.

#### 3.4.13. (6R*,14bS*)-6-(3-Fluoropyridine-2-yl)-5,6,8,9,14,14b-Hexahydroindolo[2′,3′:3,4]pyrido[1,2-c]-quinazoline (8i/T1)

Yield: present in ca. 50% of the isomer mixture **8i**/**T1** and **8i**/**C1** isolated after 0.5 reaction time with weight of 0.593 g (80%). ^1^H-NMR (DMSO-*d*_6_): 11.13 (s, 1H, (N)H-14); 8.36 (dd, *J* = 4.6 Hz and 1.2 Hz, 1H, H-6′); 7.69 (dd, *J* = 10.2 Hz and 8.2 Hz, 1H, H-4′); 7.42 (m, 1H, H-5′); 7.37 (d, *J* = 7.8 Hz, 1H, H-10); 7.30 (d, *J* = 7.8 Hz, 1H, H-13); 7.04 (t, *J* = 7.8 Hz, 1H, H-12); 7.00–6.93 (m, 3H, H-1,3,11); 6.59 (d, *J* = 7.9 Hz, 1H, H-4); 6.51 (d, *J*=3.0 Hz, 1H, H-5); 6.45 (t, *J* = 7.5 Hz, 1H, H-2); 5.61 (d, *J* = 3.0 Hz, 1H, H-6), 4.97 (br s, 1H, H-14b); 3.20 (dd, *J* = 11.8 Hz and 6.3 Hz 1H, H-8_b_); 3.01 (td, *J*=11.8 Hz and 4.2 Hz, 1H, H-8_a_); 2.84 (ddd, *J* = 15.5 Hz 11.8 Hz and 6.3 Hz, 1H, H-9_b_); 2.66 (dd, *J* = 15.5 Hz and 4.2 Hz, 1H, H-9_a_). ^13^C-NMR (DMSO-*d*_6_): 156.7 (d, *J* = 258.6 Hz, C-3′); 149.5 (d, *J* = 11.6 Hz, C-2′); 144.8 (d, *J* = 5.0 Hz, C-6′); 142.6 (C-4a); 136.3 (C-13a); 134.6 (C-14a); 127.70 and 127.67 (C-1 and C-3); 126.9 (C-9b); 125.2 (d, *J* = 4.0 Hz, C-5′); 124.2 (d, *J* = 19.1 Hz, C-5′); 121.0 (C-12); 119.3 (C-14c); 118.8 (C-11); 118.2 (C-10); 115.5 (C-2); 113.4 (C-4); 111.4 (C-13); 106.5 (C-9a); 69.1 (C-6); 50.0 (C-14b); 47.7 (C-8); 22.3 (C-9).

#### 3.4.14. (6R*,14bS*)-6-(3-Bromopyridine-2-yl)-5,6,8,9,14,14b-Hexahydroindolo[2′,3′:3,4]pyrido[1,2-c]quinazoline (8j/T1)

Light yellow solid. Yield: 0.736 g (80%) with 1 h reaction time. 0.800 g (87%) with 12 h reaction time. M.p. 219–223 °C. ^1^H-NMR (DMSO-*d*_6_): 11.07 (s, 1H, (N)H-14); 8.45 (dd, *J* = 4.6 Hz and 1.2 Hz, 1H, H-6′); 8.00 (dd, *J* = 8.3 Hz and 1.2 Hz, 1H, H-4′); 7.34 (d, *J* = 7.8 Hz, 1H, H-10); 7.26 (d, *J* = 7.8 Hz, 1H, H-13); 7.22 (dd, *J* = 8.3 Hz and 4.6 Hz, 1H, H-5′); 7.00 (t, *J* = 7.8 Hz, 1H, H-12); 6.95–6.89 (m, 3H, H-1,3,11); 6.54 (d, *J* = 7.9 Hz, 1H, H-4); 6.48 (d, *J* = 3.2 Hz, 1H, H-5); 6.39 (t, *J* = 7.5 Hz, 1H, H-2); 5.54 (d, *J* = 3.2 Hz, 1H, H-6), 4.76 (br s, 1H, H-14b); 3.13 (m, 1H, H-8_b_); 2.92 (td, *J* = 12.2 Hz and 4.0 Hz, 1H, H-8_a_); 2.84 (ddd, *J* = 15.5 Hz 12.2 Hz and 6.4 Hz, 1H, H-9_b_); 2.62 (dd, *J* = 15.5 Hz and 4.0 Hz, 1H, H-9_a_). ^13^C-NMR (DMSO-*d*_6_): 158.5 (C-2′); 147.6 (C-6′); 142.7 (C-4a); 141.7 (C-4′); 136.3 (C-13a); 134.5 (C-14a); 127.8 (C-3); 127.6 (C-1); 126.9 (C-9b); 124.9 (C-5′); 121.1 (C-12), 120.5 (C-3′); 119.2 (C-14c); 118.9 (C-11); 118.2 (C-10); 115.2 (C-2); 113.3 (C-4); 111.4 (C-13); 106.7 (C-9a); 73.0 (C-6); 49.6 (C-14b); 47.8 (C-8); 22.4 (C-9). HRMS exact mass calcd. for C_23_H_20_BrN_4_ [MH]^+^, requires *m*/*z*: 431.08658, found *m*/*z*: 431.08596.

#### 3.4.15. (6R*,14bS*)-6-[3,5-Difluorophenyl]-5,6,8,9,14,14b-Hexahydroindolo[2′,3′:3,4]pyrido[1,2-c]quinazoline (8k/T1)

Colourless solid. Yield: 0.535 g (69%) with 1 h reaction time. 0.550 g (71%) with 12 h reaction time. M.p. 208–211 °C. ^1^H-NMR (DMSO-*d*_6_): 11.03 (s, 1H, (N)H-14); 7.34–7.30 (overlapping m’s, 2H, H-6′,10); 7.27 (d, *J* = 7.8 Hz, 1H, H-13); 7.17 (ddd, *J* = 11.1 Hz, 10.6 Hz and 2.3 Hz, 1H, H-3′); 7.04 (td, *J* = 8.2 Hz, 2.3 Hz, 1H, H-5′); 7.01-6.96 (overlapping m’s, 2H, H-1,12); 6.91 (t, *J* = 7.8 Hz, 1H, H-11); 6.65 (br~d, *J*~4 Hz, 1H, H-5); 6.63 (d, *J* = 7.7 Hz, 1H, H-4); 6.48 (t, *J* = 7.7 Hz, 1H, H-2); 5.45 (br~d, *J*~4 Hz, 1H, H-6); 4.68 (br s, 1H, H-14b); 3.12 (dd, *J* = 11.4 Hz,5.6 Hz, 1H, H-8_b_); 2.89 (dd, *J* = 11.4 Hz, 3.9 Hz, 1H, H-8_a_); 2.77 (m, 1H, H-9_b_); 2.60 (dd, *J* = 15.4 Hz and 3.9 Hz, 1H, H-9_a_). ^13^C-NMR (DMSO-*d*_6_): 162.3 (dd, *J* = 243.3 Hz, 14.3 Hz, C-4′); 159.9 (dd, *J* = 250.4 Hz, 12.2 Hz, C-2′); 141.9 (C-4a); 136.3 (C-13a); 134.5 (C-14a); 130.0 (dd, *J* = 9.8 Hz, 5.6 Hz, C-6′); 128.2 (C-3); 128.0 (C-1); 127.3 (dd, *J* = 12.4 Hz, 3.3 Hz, C-1′); 126.9 (C-9b); 121.2 (C-12); 118.9 (two coalesced lines, C-11,14c); 118.2 (C-10); 116.3 (C-2); 113.5 (C-4); 111.5 (C-13); 111.1 (dd, *J* = 20.8 Hz, 3.0 Hz, C-5′); 106.5 (C-9a); 104.7 (t, *J* = 26.6 Hz, C-3′); 67.8 (C-6); 50.0 (C-14b); 47.1 (C-8); 22.2 (C-9). HRMS exact mass calcd. for C_24_H_20_F_2_N_3_ [MH]^+^, requires *m*/*z*: 388.16198, found *m*/*z*: 388.16146.

#### 3.4.16. 2-(6S,14bS,S_p_)-5,6,8,9,14,14b-Hexahydroindolo[2′,3′:3,4]pyrido[1,2-c]quinazolin-6-yl)-ferrocene-1-carboxylic acid (8m*/C1)

Light orange solid. Yield: 0.473 g (47%) with 5 h reaction time. 0.080 g (8%) with 12 h reaction time. M.p. 215–218 °C. ^1^H-NMR (DMSO-*d*_6_): 11.44 (s, 1H, (N)H-14); 7.38 (d, *J* = 7.8 Hz, 1H, H-13); 7.31 (d, *J* = 7.8 Hz, 1H, H-10); 7.24 (d, *J* = 7.5 Hz, 1H, H-1); 7.06 (t, *J* = 7.8 Hz, 1H, H-12); 7.03 (t, *J* = 7.5 Hz, 1H, H-3); 6.94–6.92 (overlapping m’s, 2H, H-4,11); 6.62 (t, *J* = 7.5 Hz, 1H, H-2); 6.25 (br s, 1H, H-5); 6.23 (br s, 1H, H-6); 5.78 (br s, 1H, H-14b); 4.89 (br s, 1H, H-5′); 4.83 (br s, 1H, H-3′); 4.48 (~t, *J*~2 Hz, 1H, H-4′ 4.27 (s, 5H, η^5^-C_5_H_5_); 2.72 (m, 1H, H-8_b_); 2.60–2.51 (overlapping m’s 3H, H-8_a_,9_a_,9_b_); ^13^C-NMR (DMSO-*d*_6_): 171.8 (2′-CO_2_H); 142.6 (C-4a); 136.8 (C-13a); 132.8 (C-14a); 128.0 (two coalesced lines, C-1,3); 126.5 (C-9b); 121.6 (C-12), 119.8 (C-14c); 119.1 (C-11); 118.4 (C-10); 118.2 (C-2); 115.9 (C-4); 111.7 (C-13); 106.6 (C-9a); 83.4 (C-1′); 74.2 (C-3′); 73.6 (C-2′); 71.4 (η^5^-C_5_H_5_); 70.5 (C-5′); 70.3 (C-6); 69.3 (C-4′); 55.9 (C-14b); 20.6 (C-9).

#### 3.4.17. Methyl-(6S,8S,14bR)-6-Phenyl-5,6,8,9,14,14b-Hexahydroindolo[2′,3′:3,4]pyrido[1,2-c]-Quinazoline-8-Carboxylate (9a/T1)

Colourless solid. Yield: 0.525 g (64%) with 2 h reaction time. M.p. 180–183 °C. ^1^H-NMR (DMSO-*d*_6_): 10.98 (s, 1H, (N)H-14); 7.43 (d, *J* = 7.4 Hz, 2H, H-2′,6′); 7.32 (t, *J* = 7.4 Hz, 2H, H-3′,5′); 7.24 (t, *J* = 7.4 Hz, 1H, H-4′); 7.36 (d, *J* = 7.8 Hz, 1H, H-10); 7.27 (d, *J* = 7.8 Hz, 1H, H-13); 7.03 (t, *J* = 7.8 Hz, 1H, H-12); 7.01 (d, *J* = 7.5 Hz, 1H, H-1); 6.97 (t, *J* = 7.5 Hz, 1H, H-3); 6.93 (t, *J* = 7.8 Hz, 1H, H-11); 6.72 (d, *J* = 3.6 Hz, 1H, H-5); 6.68 (br~d, *J*~8 Hz, 1H, H-4); 6.43 (t, *J* = 7.6 Hz, 1H, H-2); 5.27 (d, *J* = 3.6 Hz, 1H, H-6); 4.81 (br s, 1H, H-14b); 3.73 (s, 3H, 8-CO_2_CH_3_); 2.98 (br~d, *J*~8 Hz 2H, H-9_a_,9_b_). ^13^C-NMR (DMSO-*d*_6_): 173.8 (8-CO_2_CH_3_); 143.4 (C-1′); 141.9 (C-4a); 136.3 (C-13a); 134.6 (C-14a); 128.9 (C-3′,5′); 128.3 (C-3); 128.1 (C-4′); 127.9 (C-1); 127.4 (C-2′,6′); 126.6 (C-9b); 121.4 (C-12), 119.1 (C-11); 118.9 (C-14c); 118.3 (C-10); 116.3 (C-2); 113.6 (C-4); 111.5 (C-13); 104.1 (C-9a); 69.0 (C-6); 57.4 (C-8); 52.6 (8-CO_2_CH_3_); 49.9 (C-14b); 26.0 (C-9). HRMS exact mass calcd. for C_26_H_24_N_3_O_2_ [MH]^+^, requires *m*/*z*: 410.18630, found *m*/*z*: 410.18595.

#### 3.4.18. Methyl-(6S,8S,14bR)-6-(3-Trifluoromethylphenyl)-5,6,8,9,14,14b-Hexahydroindolo[2′,3′:3,4]-Pyrido[1,2-c]quinazoline-8-Carboxylate (9b/T1)

Colourless solid. Yield: 0.669 g (70%) with 2 h reaction time. M. p. 231–234 °C. ^1^H-NMR (DMSO-*d*_6_): 10.97 (s, 1H, (N)H-14); 7.75 (d, *J* = 7.6 Hz, 1H, H-6′); 7.71 (br s, 1H, H-2′); 7.63 (d, *J* = 7.6 Hz, 1H, H-4′); 7.63 (d, *J* = 7.6 Hz, 1H, H-4′); 7.59 (t, *J* = 7.6 Hz, 1H, H-5′); 7.37 (d, *J* = 7.8 Hz, 1H, H-10); 7.28 (d, *J* = 7.8 Hz, 1H, H-13); 7.03 (t, *J* = 7.8 Hz, 1H, H-12); 7.00 (dd, *J* = 8.0 Hz and 7.6 Hz, 1H, H-3); 6.94 (t, *J* = 7.8 Hz, 1H, H-11); 6.90 (d, *J* = 7.6 Hz, 1H, H-1); 6.79 (d, *J* = 3.7 Hz, 1H, H-5); 6.73 (dd, *J* = 8.0 Hz and 1.2 Hz, 1H, H-4); 6.46 (td, *J* = 7.6 Hz and 1.2 Hz, 1H, H-2); 5.38 (d, *J* = 3.7 Hz, 1H, H-6); 4.75 (br s, 1H, H-14b); 3.76 (s, 3H, 8-CO_2_CH_3_); 3.73 (dd, *J* = 11.2 Hz and 7.6 Hz, 1H, H-8); 2.97 (~d, *J*~8 Hz, 2H H-9_a_,9_b_). ^13^C-NMR (DMSO-*d*_6_): 173.9 (8-CO_2_CH_3_); 145.1 (C-1′); 141.3 (C-4a); 136.3 (C-13a); 133.5 (C-14a); 131.7 (C-6′); 130.2 (C-5′); 129.7 (qa, *J* = 31.0 Hz, C-3′); 128.4 (C-3); 127.9 (C-1); 126.7 (C-9b); 124.9 (qa, *J* = 3.5 Hz, C-4′); 124.7 (qa, *J* = 273.0 Hz, CF_3_); 123.8 (qa, *J* = 3.8 Hz, C-2′); 121.6 (C-12), 119.2 (C-11); 119.0 (C-14c); 118.3 (C-10); 116.8 (C-2); 113.8 (C-4); 111.6 (C-13); 104.2 (C-9a); 68.6 (C-6); 57.3 (C-8); 52.6 (8-CO_2_CH_3_); 49.8 (C-14b); 26.3 (C-9). HRMS exact mass calcd. for C_27_H_23_F_3_N_3_O_2_ [MH]^+^, requires *m*/*z*: 478.17369, found *m*/*z*: 478.17327.

#### 3.4.19. Methyl-(6S,8S,14bR)-6-[3,5-Bis(trifluoromethyl)phenyl]-5,6,8,9,14,14b-Hexahydroindolo-[2′,3′:3,4]pyrido[1,2-c] quinazoline-8-Carboxylate (9c/T1)

Colourless solid. Yield: 0.677 g (62%) with 2 h reaction time. M.p. 281–284 °C. ^1^H-NMR (DMSO-*d*_6_): 10.98 (s, 1H, (N)H-14); 8.05 (br s, 2H, H-2′,6′); 8.02 (br s, 1H, H-4′); 7.37 (d, *J* = 7.8 Hz, 1H, H-10); 7.29 (d, *J* = 7.8 Hz, 1H, H-13); 7.02 (br~t, *J*~8 Hz, 1H, H-12); 7.01–6.97 (overlapping m’s, 3H, H-1,3,5); 6.93 (t, *J* = 7.8 Hz, 1H, H-11); 6.77 (br~d, *J*~8 Hz, 1H, H-4); 6.46 (td, *J* = 7.8 Hz and 1.2 Hz, 1H, H-2); 5.50 (d, *J* = 3.3 Hz, 1H, H-6); 4.75 (br s, 1H, H-14b); 3.78 (s, 3H, 8-CO_2_CH_3_); 3.76 (dd, *J* = 10.9 Hz and 7.5 Hz, 1H, H-8); 2.97 (~d, *J*~8 Hz, 2H H-9_a_,9_b_). ^13^C-NMR (DMSO-*d*_6_): 173.9 (8-CO_2_CH_3_); 147.5 (C-1′); 141.0 (C-4a); 136.3 (C-13a); 134.1 (C-14a); 130.7 (qa, *J* = 32.6 Hz, C-3′,5′); 128.2 (br, C-2′,6′); 128.3 (C-3); 128.1 (C-1); 126.8 (C-9b); 123. 7 (qa, *J* = 273.8 Hz, 3′,5′-CF_3_); 121.9 (br, C-4′); 121.4 (C-12), 119.2 (C-14c); 119.0 (C-11); 118.3 (C-10); 116.8 (C-2); 113.8 (C-4); 111.6 (C-13); 104.3 (C-9a); 68.5 (C-6); 57.1 (C-8); 52.6 (8-CO_2_CH_3_); 49.7 (C-14b); 26.3 (C-9). HRMS exact mass calcd. for C_28_H_22_F_6_N_3_O_2_ [MH]^+^, requires *m*/*z*: 546.16107, found *m*/*z*: 546.16123.

#### 3.4.20. Methyl-(6S,8S,14bR)-6-(4-Nitrophenyl)-5,6,8,9,14,14b-Hexahydroindolo[2′,3′:3,4]pyrido[1,2-c]-Quinazoline-8-Carboxylate (9d/T1)

Light oange solid. Yield: 0.709 g (73%) with 2 h reaction time. M.p. 235–237 °C. ^1^H-NMR (DMSO-*d*_6_): 11.00 (s, 1H, (N)H-14); 8.22 (d, *J* = 8.7 Hz, 2H, H-3′,5′); 7.69 (d, *J* = 8.7 Hz, 2H, H-2′,6′); 7.36 (d, *J* = 7.8 Hz, 1H, H-10); 7.27 (d, *J* = 7.8 Hz, 1H, H-13); 7.02 (t, *J* = 7.8 Hz, 1H, H-12); 6.99 (t, *J* = 7.5 Hz, 1H, H-3); 6.93 (t, *J* = 7.8 Hz,1H, H-11); 6.88 (d, *J* = 7.5 Hz, 1H, H-1); 6.87 (d, *J*= 3.9 Hz, 1H, H-5); 6.73 (d, *J* = 7.5 Hz, 1H, H-4); 6.45 (t, *J* = 7.5 Hz, 1H, H-2); 5.42 (d, *J* = 3.9 Hz, 1H, H-6), 4.71 (br s, 1H, H-14b); 3.76 (s, 3H, 8-CO_2_CH_3_); 3.71 (dd, *J* = 9.3 Hz, 6.1 Hz, 1H, H-8_a_); 2.94 (dd, *J* = 14.9 Hz 9.3 Hz, 1H, H-9_b_); 2.88 (dd, *J* = 14.9 Hz 6.1 Hz, 1H, H-9_a_). ^13^C-NMR (DMSO-*d*_6_): 173.8 (8-CO_2_CH_3_); 151.3 (C-1′); 147.5 (C-4′); 141.1 (C-4a); 136.3 (C-13a); 133.3 (C-14a); 128.8 (C-2′,6′); 128.4 (C-3); 127.8 (C-1); 126.5 (C-9b); 124.3 (C-3′,5′); 121.5 (C-12), 119.2 (C-11); 118.9 (C-14c); 118.3 (C-10); 116.8 (C-2); 113.9 (C-4); 111.6 (C-13); 104.2 (C-9a); 68.6 (C-6); 57.3 (C-8); 52.7 (8-CO_2_CH_3_); 50.0 (C-14b); 26.5 (C-9). HRMS exact mass calcd. for C_26_H_23_N_4_O_4_ [MH]^+^, requires *m*/*z*: 455.17138, found *m*/*z*: 455.17071.

#### 3.4.21. Methyl-(6S,8S,14bR)-6-(3,4,5-Trimethoxyphenyl)-5,6,8,9,14,14b-Hexahydroindolo[2′,3′:3,4]-pyrido[1,2-c]quinazoline-8-Carboxylate (9f/T1)

Colourless solid. Yield: 0.709 g (71%) with 2 h reaction time. M.p. 240–243 °C. ^1^H-NMR (DMSO-*d*_6_): 10.86 (s, 1H, (N)H-14); 7.36 (d, *J* = 7.8 Hz, 1H, H-10); 7.29 (d, *J* = 7.8 Hz, 1H, H-13); 7.02 (t, *J* = 7.8 Hz, 1H, H-12); 6.00-6.95 (overlapping m’s, H-1,3); 6.93 (t, *J* = 7.8 Hz, 1H, H-11); 6.76 (s, 2H, H-2′,6′); 6.67 (d, *J* = 7.6 Hz, 1H, H-4); 6.63 (d, *J* = 3.0 Hz, H-5); 6.48 (t, *J*=7.7 Hz, 1H, H-2); 5.14 (d, *J* = 3.0 Hz, 1H, H-6), 4.95 (br s, 1H, H-14b); 3.71 (dd, *J* = 8.4 Hz, 5.3 Hz, 1H, H-8_a_); 3.70 (s, 3H, 8-CO_2_CH_3_); 3.65 (s, 6H, 3′,5′-OCH_3_); 3.61 (s, 3H, 4′-OCH_3_); 2.95 (dd, *J* = 15.4 Hz 8.4 Hz, 1H, H-9_b_); 2.92 (dd, *J* = 15.4 Hz 5.3 Hz, 1H, H-9_a_). ^13^C-NMR (DMSO-*d*_6_): 173.8 (8-CO_2_CH_3_); 153.5 (C-3′,5′); 137.7 (C-4′); 142.0 (C-4a); 137.7 (C-4′); 136.3 (C-13a); 133.9 (C-14a); 128.2 (C-3); 128.0 (C-1); 126.7 (C-9b); 121.4 (C-12), 119.0 (C-11); 118.8 (C-14c); 118.3 (C-10); 116.5 (C-2); 113.7 (C-4); 111.7 (C-13); 105.1 (C-2′,6′); 104.8 (C-1′); 104.1 (C-9a); 69.1 (C-6); 60.5 (4′-OCH_3_); 56.9 (C-8); 56.5 (3′,5′-OCH_3_); 52.7 (8-CO_2_CH_3_); 50.3 (C-14b); 24.8 (C-9). HRMS exact mass calcd. for C_29_H_30_N_3_O_5_ [MH]^+^, requires *m*/*z*: 500.21800 found *m*/*z*: 500.21791.

#### 3.4.22. Methyl-(6S,8S,14bR)-6-Ferrocenyl-5,6,8,9,14,14b-Hexahydroindolo[2′,3′:3,4]pyrido[1,2-c]-Quinazoline-8-Carboxylate (9g/T1)

Light orange solid. Yield: 0.538 g (52%) with 2 h reaction time. M.p. 201–205 °C. ^1^H-NMR (DMSO-*d*_6_): 11.12 (s, 1H, (N)H-14); 7.33 (d, *J* = 7.8 Hz, 1H, H-10); 7.26 (d, *J* = 7.8 Hz, 1H, H-13); 7.00 (t, *J* = 7.8 Hz, 1H, H-12); 6.95 (t, *J* = 7.5 Hz, 1H, H-3); 6.90 (br~t, *J*~8 Hz, 1H, H-11); 6.87 (d, *J* = 7.5 Hz, 1H, H-1); 6.78 (d, *J* = 7.9 Hz, 1H, H-4); 6.58 (d, *J* = 3.7 Hz, 1H, H-5); 6.39 (t, *J* = 7.5 Hz, 1H, H-2); 5.30 (d, *J* = 3.7 Hz, 1H, H-6), 4.98 (br s, 1H, H-14b); 4.22 (dt, *J* = 2.3 Hz, 1.5 Hz, 1H, H-2′); 4.18 (s, 5H, η^5^-C_5_H_5_); 4.11 (qa, *J* = 2.3 Hz, 1H, H-5′); 4.03 (~t, *J*~2 Hz, 2H, H-3′,4′); 3.77 (s, 3H, 8-CO_2_CH_3_); 3.59 (dd, *J* = 10.3 Hz, 5.3 Hz, 1H, H-8_a_); 2.88 (dd, *J* = 14.9 Hz 5.3 Hz, 1H, H-9_a_); 2.83 (dd, *J* = 14.9 Hz 10.3 Hz, 1H, H-9_b_). ^13^C-NMR (DMSO-*d*_6_): 174.7 (8-CO_2_CH_3_); 142.2 (C-4a); 136.3 (C-13a); 134.3 (C-14a); 128.1 (C-3); 127.7 (C-1); 126.5 (C-9b); 121.4 (C-12), 118.9 (C-11); 118.5 (C-14c); 118.3 (C-10); 116.0 (C-2); 113.4 (C-4); 111.5 (C-13); 104.3 (C-9a); 91.2 (C-1′); 69.3 (η^5^-C_5_H_5_); 68.9 (C-5′); 68.1 and 68.2 (C-3′,4′); 67.1 (C-6); 66.9 (C-2′); 57.2 (C-8); 52.5 (8-CO_2_CH_3_); 49.6 (C-14b); 26.8 (C-9). HRMS exact mass calcd. for C_30_H_28_FeN_3_O_2_ [MH]^+^, requires *m*/*z*: 518.15254, found *m*/*z*: 518.15078.

#### 3.4.23. Methyl-(6S,8S,14bR)-6-(pyridine-2-yl)-5,6,8,9,14,14b-Hexahydroindolo[2′,3′:3,4]pyrido[1,2-c]-Quinazoline-8-Carboxylate (9h/T1)

Yellow solid. Yield: 0.460 g (56%) with 2 h reaction time. M.p. 190–193 °C. ^1^H-NMR (DMSO-*d*_6_): 10.91 (s, 1H, (N)H-14); 8.50 (br ~d, *J*~4 Hz, 1H, H-6′); 7.79 (td, *J* = 7.7 Hz, 1.3 Hz, 1H, H-4′); 7.50 (d, *J* = 7.8 Hz, 1H, H-3′) 7.36 (d, *J* = 7.8 Hz, 1H, H-10); 7.30-7.27 (overlapping m’s, H-5′,13); 7.02 (t, *J* = 7.8 Hz, 1H, H-12); 6.99-6.95 (overlapping m’s, H-1,3); 6.93 (t, *J* = 7.8 Hz, 1H, H-11); 6.63 and 6.62 (partly overlapping d’s, *J* = 7.6 Hz and 3.0 Hz, resp., 2 × 1H, H-4 and H-5); 6.47 (t, *J* = 7.6 Hz, 1H, H-2); 5.24 (d, *J* = 3.0 Hz, 1H, H-6), 4.94 (br s, 1H, H-14b); 3.69 (s, 3H, 8-CO_2_CH_3_); 3.68 (dd, *J* = 8.8 Hz, 5.6 Hz, 1H, H-8_a_); 2.98 (dd, *J* = 15.8 Hz 5.6 Hz, 1H, H-9_b_); 2.91 (dd, *J* = 15.8 Hz 8.8 Hz, 1H, H-9_a_). ^13^C-NMR (DMSO-*d*_6_): 173.6 (8-CO_2_CH_3_); 161.3 (C-2′); 149.2 (C-6′); 142.4 (C-4a); 137.5 (C-4′); 136.3 (C-13a); 133.6 (C-14a); 128.2 (C-3); 127.8 (C-1); 126.6 (C-9b); 123.5 (C-5′); 122.5 (C-3′); 121.4 (C-12), 119.1 (C-11); 119.0 (C-14c); 118.3 (C-10); 116.3 (C-2); 113.9 (C-4); 111.6 (C-13); 104.1 (C-9a); 70.8 (C-6); 57.6 (C-8); 52.5 (8-CO_2_CH_3_); 50.6 (C-14b); 25.3 (C-9). HRMS exact mass calcd. for C_25_H_23_N_4_O_2_ [MH]^+^, requires *m*/*z*: 411.18155, found *m*/*z*: 411.18091.

#### 3.4.24. 2-((6S,8S,14bR,S_p_)-8-(methoxycarbonyl)-5,6,8,9,14,14b-Hexahydroindolo[2′,3′:3,4]pyrido[1,2-c]quinazolin-6-yl)-Ferrocene-1-Carboxylic acid (9m/T1).

Since upon attempted chromatographic purifications this compound underwent uncontrolled decomposition, this product was identified as the major component (ca. 60% by ^1^H-NMR) in the isolated sample (0.250 g) contaminated with undefined components. Due to the presence of these unidentified components with uncertain composition, 10–15% can be given as an approximate yield of this unstable product. ^1^H-NMR (DMSO-*d*_6_): 11.19 (s, 1H, (N)H-14); 7.36 (d, *J* = 7.8 Hz, 1H, H-10); 7.28 (d, *J* = 7.8 Hz, 1H, H-13); 7.04 (t, *J* = 7.8 Hz, 1H, H-12); 7.01 (d, *J* = 7.5 Hz, 1H, H-3); 6.96-6.94 (overlapping m’s, 2H, H-1,11); 6.75 (d, *J* = 7.5 Hz, 1H, H-4); 6.48 (t, *J* = 7.5 Hz, 1H, H-2); 6.35 (br s, 1H, H-5); 5.93 (d, *J* = 3.2 Hz, 1H, H-6); 5.08 (br s, 1H, H-14b); 4.73 (br s, 1H, H-3′); 4.34 (~t, *J*~2 Hz, 1H, H-4′); 4.31 (br s, 1H, H-5′); 4.24 (s, 5H, η^5^-C_5_H_5_); 3.80 (s, 3H, 8-CO_2_CH_3_); 3.70 (dd, *J* = 8.2 Hz, 5.1 Hz, 1H, H-8_a_); 2.94-2.89 (overlapping m’s 2H, H-9_a_,9_b_); ^13^C-NMR (DMSO-*d*_6_): 172.5 (8-CO_2_CH_3_); 172.1 (2′-CO_2_H); 141.2 (C-4a); 136.5 (C-13a); 132.5 (C-14a); 128.6 (C-3); 127.9 (C-1); 126.3 (C-9b); 121.6 (C-12); 119.3 (C-11); 118.4 (C-10); 117.7 (C-14c); 116.7 (C-2); 113.9 (C-4); 111.5 (C-13); 104.3 (C-9a); 73.7 (C-3′); 73.2 (C-1′); 72.5 (C-2′); 71.3 (η^5^-C_5_H_5_); 70.7 (C-5′); 69.5 (C-4′); 69.2 (C-6); 57.3 (C-8); 52.9 (8-CO_2_CH_3_); 50.9 (C-14b); 26.3 (C-9). HRMS was not measured for this sample.

#### 3.4.25. (5bR*,15bS*)-5,5b,17,18-Tetrahydroindolo[2′,3′:3,4]pyrido[1,2-c]isoindolo[2,1-a]quinazolin-11(15bH)-One (10/C1)

Colourless solid. Yield: 0.667 g (88%) with 5 h reaction time. M.p. 272–274 °C. ^1^H-NMR (DMSO-*d*_6_): 11.47 (s, 1H, (N)H-5); 8.54 (dd, *J* = 8.2 Hz and 1.1 Hz, 1H, H-9); 7.85 (d, *J* = 7.6 Hz, 1H, H-12); 7.74 (t, *J* = 7.6 Hz, 1H, H-14); 7.69 (d, *J* = 7.6 Hz, 1H, H-15); 7.64 (t, *J* = 7.6 Hz, 1H, H-13); 7.56 (d, *J* = 7.6 Hz, 1H, H-6); 7.40 (d, *J* = 7.8 Hz, 1H, H-4); 7.33 (d, *J* = 7.8 Hz, 1H, H-1); 7.30 (dd, *J* = 8.2 Hz and 7.6 Hz, 1H, H-8); 7.09 (t, *J* = 7.8 Hz, 1H, H-7); 7.07 (t, *J* = 7.9 Hz, 1H, H-3); 6.93 (ddd, *J* = 7.9 Hz, 7.2 Hz and 1.0 Hz, 1H, H-2); 6.32 (s, 1H, H-15b); 5.75 (s, 1H, H-5b); 2.89 (dt, *J* = 14.9 Hz and 2.8 Hz, 1H, H-17_a_); 2.62 (m, 1H, H-18_b_); 2.49 (dt, *J* = 14.9 Hz and 2.8 Hz, 1H, H-17_b_); 2.46 (m, 1H, H-18_a_); ^13^C-NMR (DMSO-*d*_6_): 165.5 (C-11); 140.1 (C-15a); 136.6 (C-4a); 135.1 (C-9a); 133.6 (C-14); 133.5 (two coalesced lines, C-5a, C-11a); 130.5 (C-13); 128.5 (C-6); 128.1 (C-8); 126.6 (C-18b); 124.4 (C-5c); 124.3 (C-5); 124.2 (C-7); 123.9 (C-12); 121.6 (C-3); 119.1 (C-2); 118.6 (C-1); 118.2 (C-9); 111.7 (C-4); 107.5 (C-18a); 77.0 (C-15b); 57.1 (C-5b); 38.4 (C-17); 21.4 (C-18). HRMS exact mass calcd. for C_25_H_20_N_3_O [MH]^+^, requires *m*/*z*: 378.16009, found *m*/*z*: 378.15966.

#### 3.4.26. Methyl (5bR,15bS,17S)-11-Oxo-5,5b,11,15b,17,18-Hexahydroindolo[2′,3′:3,4]pyrido[1,2-c]-isoindolo[2,1-a]quinazoline-17-Carboxylate (11/C1)

Colourless solid. Yield: 0.619 g (71%) with 5 h reaction time. M.p. 239–242 °C. ^1^H-NMR (DMSO-*d*_6_): 11.54 (s, 1H, (N)H-5); 8.65 (dd, *J* = 8.2 Hz and 1.1 Hz, 1H, H-9); 7.79 (dd, *J* = 7.6 Hz and 1.1 Hz, 1H, H-12); 7.65 (td, *J* = 7.6 Hz and 1.1 Hz 1H, H-14); 7.60 (d, *J* = 7.6 Hz, 1H, H-15); 7.58 (td, *J* = 7.6 Hz and 1.1 Hz, 1H, H-13); 7.42 (d, *J* = 8.2 Hz, 1H, H-4); 7.35 (two coalesced d’s, *J*~8 Hz, 2H, H-1,6); 7.31 (ddd, *J* = 8.2 Hz, 7.6 Hz and 1.1 Hz, 1H, H-8); 7.09 (ddd, *J* = 8.2 Hz, 7.9 Hz and 1.0 Hz, 1H, H-3); 7.05 (td, *J* = 7.6 Hz and 1.1 Hz, 1H, H-7); 6.95 (t, *J*=7.9 Hz, 1H, H-2); 6.48 (s, 1H, H-15b); 5.92 (s, 1H, H-5b); 3.48 (dd, *J* = 8.9 Hz and 5.2 Hz, 1H, H-17_a_); 2.98 (dd, *J* = 15.4 Hz and 8.9 Hz, 1H, H-18_b_); 2.83 (s, 3H, 17-CO_2_CH_3_); 2.70 (dd, *J* = 15.4 Hz and 5.2 Hz, 1H, H-18_a_). ^13^C-NMR (DMSO-*d*_6_): 172.5 (17-CO_2_CH_3_); 165.2 (C-11); 139.4 (C-15a); 136.5 (C-4a); 135.5 (C-11a); 133.7 (C-9a); 133.2 (C-5a); 132.4 (C-14); 130.4 (C-13); 128.4 (C-8); 128.2 (C-6); 126.25 (C-18b); 126.20 (C-15); 124.6 (C-5c); 124.1 (C-7); 123.3 (C-12); 121.8 (C-3); 119.3 (C-2); 118.5 (C-1); 117.5 (C-9); 105.1 (C-18a);; 77.1 (C-15b); 58.7 (C-5b); 54.5 (C-17); 51.9 (17-CO_2_CH_3_); 26.5 (C-18). HRMS exact mass calcd. for C_27_H_22_N_3_O_3_ [MH]^+^, requires *m*/*z*: 436.16557, found *m*/*z*: 436.16519.

## 4. Conclusions

Using a straightforward three-step reaction sequence comprising Pictet-Spengler annelation followed by nitro-reduction and aldehyde-mediated cyclization, tryptamine and l-tryptophan were converted into a series of partly saturated novel polycondensed β-carbolines obtained as racemic mixtures and single enantiomers, respectively, with well-defined conformations identified by single crystal X-ray analysis and NMR measurements. On the basis of the results of comparative DFT studies, a plausible mechanism was proposed for the final cyclization that might account for the observed stereochemical outcome controlled by the electronic properties and steric bulk of the reactants. The prepared compounds were evaluated for their antiproliferative/cytotoxic activity on human malignant cell lines PANC-1, COLO-205, A2058 and EBC-1 disclosing characteristic structure-activity relationships and cell-selectivity that might be utilized in rational structure-refinement in the development of more potent antiproliferative agents with enhanced activity and selectivity. Accordingly, besides the comparative tests of the particular tryptamine-derived enantiomers envisaged to be available by stereoselective cyclization procedures (using e.g., organocatalysis) or chromatographic separation, the synthesis and evaluation of the d-tryptophan-derived enantiomers and further models, with an array of substituents in the different regions of the heterocyclic skeletons, will be the subjects of subsequent papers.

## Figures and Tables

**Table 1:** Isolated yields of pentacyclic compounds types **8** and **9** presented in Scheme 1.

Product	Yield (Reaction Time)	Product	Yield (Reaction Time)
**8a**/**T1**	37% (0.5 h)/77% (12 h)	**8i**/**C1**/**T1**	80% (0.5 h) ^e^/35% (12 h) ^e^
**8b**/**T1**	75% (1 h)/72% (12 h)	**8j**/**T1**	80% (1 h)/87% (12 h)
**8c**/**T1**	79% (1 h)/76% (12 h)	**8k**/**T1**	69% (1 h)/71% (12 h)
**8d**/**T1**	74% (1 h)/78% (12 h)	**9a**/**T1**	64% (2 h)
**8e**/**T1**	80% (1 h)/75% (12 h)	**9b**/**T1**	70% (2 h)
**8f**/**C1**	63% (0.5 h)	**9c**/**T1**	62% (2 h)
**8f**/**C1**/**T1**	80% (4 h) ^a^/75% (8 h) ^b^	**9d**/**T1**	73% (2 h)
**8g**/**C1**	39% (0.5 h)	**9f**/**T1**	71% (2 h)
**8g**/**C1**/**T1**	52% (4 h) ^c^	**9g**/**T1**	52% (2 h)
**8h**/**C1**/**T1**	72% (0.5 h) ^d^/25% (12 h) ^d^	**9h**/**T1**	56% (2 h)

^a^ For a ca. 1:3 mixture of *cis* (**C1**) and *trans* (**T1**) diastereomers. ^b^ For a ca. 1:4 mixture of *cis* (**C1**) and *trans* (**T1**) diastereomers. ^c^ For a ca. 3:7 mixture of *cis* (**C1**) and *trans* (**T1**) diastereomers. ^d^ For a ca. 4:3 mixture of *cis* (**C1**) and *trans* (**T1**) diastereomers. ^e^ For a ca. 1:1 mixture of *cis* (**C1**) and *trans* (**T1**) diastereomers.

**Table 2:** Relative stability of diastereomer pairs **8**/**T1**-**8**/**C1** calculated by DFT modelling.

	ΔG(T1-C1) [kcal/mol]		ΔG(T1-C1) [kcal/mol]		ΔG(T1-C1) [kcal/mol]
**8a**	−1.13	**8e**	−3.06	**8i**	+0.19
**8b**	−1.65	**8f**	−1.52	**8j**	−0.75
**8c**	−1.58	**8g**	−0.47	**8k**	−1.73
**8d**	−1.48	**8h**	+0.34		

**Table 3:** Relative stability of selected isomer pairs to rationalize of the substituent- and time-dependent interconversion of *cis*- and *trans*-pentacycles **8**/**C1** and **8**/**T1**, as resulted from DFT modeling studies.

	Relative Stability of Intermediate Pairs Expressed in ΔG [kcal/mol]			
Diastereoselectivity of primary coupling to **12^+^**	**12^+^a**/**R**−**12^+^a**/**S** −0.68	**12^+^d**/**R**−**12^+^d**/**S** +0.17	**12^+^f**/**R**−**12^+^f**/**S** −0.70	**12^+^g**/**R**−**12^+^g**/**S** −0.97
Diastereoselectivity of primary coupling to **13^+^**	**13^+^a**/**R**−**13^+^a**/**S** −0.21	**13^+^d**/**R**−**13^+^d**/**S** ***+2.83***	**13^+^f**/**R**−**13^+^f**/**S** −3.41	**13^+^g**/**R**−**13^+^g**/**S** −0.92
Readiness of rotation **12^+^**/**R**→**13^+^**/**R** ^a^	***13^+^a***/***R**−**12^+^a**/**R*** *−1.48*	**13^+^d**/**R**−**12^+^d**/**R** +0.79	***13^+^f***/***R**−**12^+^f**/**R*** *−2.21*	***13^+^g***/***R**−**12^+^g**/**R*** *−1.77*
Readiness of rotation **12^+^**/**S**→**13^+^**/**S** ^a^	**13^+^a**/**S**−**12^+^a**/**S** −1.95	***13^+^d***/***S**−**12^+^d**/**S*** *−1.87*	**13^+^f**/**S**−**12^+^f**/**S** +0.50	**13^+^g**/**S**−**12^+^g**/**S** −1.82
Readiness of isomerisation **14^+^**/**E**→**15^+^**	**15^+^a**−**14^+^a**/**E** +2.42	**15^+^d**−**14^+^d**/**E** +1.04	**15^+^f**−**14^+^f**/**E** +10.24	**15^+^g**−**14^+^g**/**E** +13.19
Readiness of isomerisation **15^+^**→**14^+^/Z**	**14^+^a/Z**−**15^+^a** −1.61	**14^+^d/Z**−**15^+^d** +0.86	**14^+^f/Z**−**15^+^f** −8.06	**14^+^g/Z**−**15^+^g** −12.04
Preference of isomerization pathway via **15^+^** over that via **16^+^**	**16^+^a**−**14^+^a** +46.10 ^b^/+48.02 ^c^	**16^+^d**−**14^+^d** +35.54 ^b^/+36.76 ^c^	**16^+^f**−**14^+^f** +50.63 ^b^/+52.24 ^c^	**16^+^g**−**14^+^g** +51.93 ^b^/+53.35 ^c^

^a^ The value can also be considered as a selectivity descriptor for the alternative primary coupling modes **5**+**7^+^**→**12^+^** and **5**+**7^+^** →**13^+^** disclosing the latter as the preferred one (highlighted by italics). ^b^ Refers to **16^+^**−1**4^+^/Z**. ^c^ Refers to **16^+^**−**14^+^/E**.

**Table 4:** In vitro cytotoxic effect of novel polycondensed β-carbolines types **8–11** ^a^, on four human malignant cell lines characterised by half-maximal inhibitory concentration (IC_50_) values.

	IC_50_ [M]			
	PANC-1	COLO-205	A2058	EBC-1
**8a/T1**	7.0 × 10^−6^ ± 1.9 × 10^−6^	5.6 × 10^−6^ ± 5.6 × 10^−7^	1.4 × 10^−5^ ± 3.2 × 10^−6^	6.4 × 10^−6^ ± 1.1 × 10^−6^
**9a/T1**	> 10^−4^	> 10^−4^	> 10^−4^	> 10^−4^
**8b/T1**	2.8 × 10^−6^ ± 1.0 × 10^−6^	1.5 × 10^−6^ ± 5.9 × 10^−7^	2.5 × 10^−6^ ± 4.0 × 10^−7^	4.7 × 10^−6^ ± 4.1 × 10^−7^
**9b/T1**	> 10^−4^	> 10^−4^	> 10^−4^	> 10^−4^
**8c/T1**	2.3 × 10^−5^ ± 1.1 × 10^−6^	5.0 × 10^−5^ ± 1.9 × 10^−6^	2.5 × 10^−5^ ± 9.5 × 10^−7^	2.5 × 10^−5^ ± 5.8 × 10^−7^
**9c/T1**	2.5 × 10^−5^ ± 1.2 × 10^−6^	> 5.0 × 10^−5^	> 5.0 × 10^−5^	> 5.0 × 10^−5^
**8d/T1**	> 5.0 × 10^−5^	> 5.0 × 10^−5^	> 5.0 × 10^−5^	> 5.0 × 10^−5^
**9d/T1**	> 5.0 × 10^−5^	> 5.0 × 10^−5^	> 5.0 × 10^−5^	> 5.0 × 10^−5^
**8e/T1**	2.6 × 10^−5^ ± 1.2 × 10^−6^	> 5.0 × 10^−5^	> 5.0 × 10^−5^	> 5.0 × 10^−5^
**8f/C1**	5.2 × 10^−6^ ± 2.3 × 10^−6^	2.5 × 10^−6^ ± 9.4 × 10^−8^	2.1 × 10^−6^ ± 1.9 × 10^−7^	4.1 × 10^−6^ ± 7.8 × 10^−7^
**8f/C1/T1 ^b^**	> 5.0 × 10^−5^	> 5.0 × 10^−5^	3.6 × 10^−5^ ± 1.2510^−6^	1.6 × 10^−5^ ± 3.0 × 10^−6^
**9f/T1**	1.3 × 10^−5^ ± 1.2 × 10^−5^	2.0 × 10^−5^ ± 7.6 × 10^−7^	2.6 × 10^−5^ ± 1.6 × 10^−6^	1.1 × 10^−5^ ± 5.2 × 10^−6^
**8g/C1**	> 10^−4^	> 10^−4^	> 10^−4^	> 10^−4^
**8g/C1/T1 ^c^**	> 5.0 × 10^−5^	3.9 × 10^−5^ ± 1.1 × 10^−6^	5.0 × 10^−5^ ± 5.4 × 10^−6^	2.3 × 10^−5^ ± 1.1 × 10^−6^
**9g/T1**	> 5.0 × 10^−5^	> 5.0 × 10^−5^	> 5.0 × 10^−5^	> 5.0 × 10^−5^
**8h/C1/T1 ^d^**	> 5.0 × 10^−5^	> 5.0 × 10^−5^	> 5.0 × 10^−5^	4.7 × 10^−5^ ± 2.1 × 10^−6^
**9h/T1**	> 5.0 × 10^−5^	> 5.0 × 10^−5^	> 5.0 × 10^−5^	> 5.0 × 10^−5^
**8i/C1/T1 ^e^**	> 5.0 × 10^−5^	> 5.0 × 10^−5^	> 5.0 × 10^−5^	> 5.0 × 10^−5^
**8j/T1**	> 5.0 × 10^−5^	> 5.0 × 10^−5^	1.4 × 10^−5^ ± 3.2 × 10^−6^	5.5 × 10^−6^ ± 1.9 × 10^−7^
**8k/T1**	> 5.0 × 10^−5^	> 5.0 × 10^−5^	> 5.0 × 10^−5^	> 5.0 × 10^−5^
8m*/C1	> 5.0 × 10^−5^	> 5.0 × 10^−5^	3.8 × 10^−5^ ± 8.8 × 10^−7^	3.2 × 10^−5^ ± 2.5 × 10^−5^
**10/C1**	> 10^−4^	> 10^−4^	> 10^−4^	> 10^−5^
**11/C1**	2.9 × 10^−5^ ± 2.4 × 10^−6^	> 5.0 × 10^−5^	5.6 × 10^−6^ ± 1.3 × 10^−7^	2.1 × 10^−5^ ± 1.5 × 10^−6^

^a^ Compounds **8a**–**g**,**j**,**k**/**T1**, **8f**,**g**/**C1** and **10**/**C1** were measured in racemic form, while compounds 8m*/**C1**, **9a**–**d**,**f**–**h**/**T1** and **11**/**C1** were tested as single enantiomers. ^b^ Approximately 1:4 mixture of *cis* (**C1**) and *trans* (**T1**) diastereomers. ^c^ Approximately 3:7 mixture of *cis* (**C1**) and *trans* (**T1**) diastereomers. ^d^ Approximately 4:3 mixture of *cis* (**C1**) and *trans* (**T1**) diastereomers. ^e^ Approximately 1:1 mixture of *cis* (**C1**) and *trans* (**T1**) diastereomers.

## Supplementary Materials

Description of cell culture and viability assays; copies of NMR spectra; details of x-ray analysis of **9f/T1**.
