# Supplementary material for: Novel Polycondensed Partly Saturated β-Carbolines Including Ferrocene Derivatives: Synthesis, DFT-Supported Structural Analysis, Mechanism of Some Diastereoselective Transformations and a Preliminary Study of their In Vitro Antiproliferative Effects
Source: Molecules. 2020 Mar 31;25(7):1599. doi: 10.3390/molecules25071599 (PMC7181298; doi:10.3390/molecules25071599)
Supplement: Supplementary file 1 [file molecules-25-01599-s001.pdf]

Novel polycondensed partly saturated β-carbolines including ferrocene derivatives: synthesis; DFT-supported structural analysis and mechanism of diastereoselective transformations; a preliminary study on *in vitro* antiproliferative effects

Kinga Judit Fodor [1], Dániel Hutai [1], Tamás Jernei [2], Angéla Takács [3], Zsófia Szász [3], Máté Sulyok-Eiler[4], Veronika Harmat [4], Rita Oláh Szabó [2], Gitta Schlosser [2,5], Ferenc Hudecz [1,2], László Kőhidai [3] and Antal Csámpai [1*]

1 Department of Organic Chemistry, Eötvös Loránd University (ELTE) Budapest Pázmány P. sétány 1/A, H-1117, Hungary; fodorkinga90@gmail.com (K.J.F.); hutaidani@gmail.com (D.H.); fhudecz@caesar.elte.hu (F.H.);

2 MTA-ELTE Research Group of Peptide Chemistry, Budapest Pázmány P. sétány 1/A, H-1117, Hungary; jernei91@gmail.com (T.J.); rita.olah.szabo@gmail.com (R.O.S.); sch@chem.elte.hu (G.S.); fhudecz@caesar.elte.hu (F.H.)

3 Department of Genetics, Cell- and Immunobiology, Semmelweis University, H-1089 Budapest, Nagyvárad tér 4, Hungary; angela.takacs1@gmail.com (A.T.); szaszzsoccii@gmail.com (Z.S.); kohlasz2@gmail.com (L.K.)

4 Laboratory of Structural Chemistry and Biology, Institute of Chemistry, Eötvös Loránd University, Budapest Pázmány P. sétány 1/A, H-1117, Hungary; etammate95@gmail.com (M.S.-E.); veronika@chem.elte.hu; (V.H.)

5 Department of Analytical Chemistry, Eötvös Loránd University (ELTE), Budapest, Pázmány P. sétány 1/A, H-1117, Hungary; sch@chem.elte.hu (G.S.)

* Correspondence: csampai@caesar.elte.hu; Tel.: +36-01-372-2500/6591

Supplementary Material

Content:

S.1. Cell cultures pp. 1 – 1

S.2. Viability assays pp. 2 - 3

S.3. Copies of the NMR spectra of the products pp. 4 – 44

S.4. Crystallographic study on compound 9f/T1 pp. 45 – 50

S.1. Cell Cultures

The PANC-1 (human pancreatic carcinoma of ductal origin), COLO 205 (human colorectal adenocarcinoma), A2058 (human metastatic melanoma) obtained from European Collection of Authenticated Cell Cultures (ECACC, Salisbury, UK) and EBC-1 (human lung squamous cell carcinoma) purchased from Japanese Research Resources Bank (Tokyo, Japan) were used to determine the tumor growth inhibitory effects of -carbolines. PANC-1 cells were maintained in Dulbecco's Modified Eagle Medium (DMEM, Lonza, Basel, Switzerland); for the culturing of COLO-205 cell line DMEM medium formulated with 4500 mg/L d-glucose was used; EBC-1 cells were cultured in DMEM medium containing 1% non-essential amino acids (NEAA, Gibco®/Invitrogen Corporation, New York, NY, USA), 1 mM sodium pyruvate (Sigma-Aldrich, St. Louis, MO, USA), while A2058 cell line was grown in RPMI 1640 (Lonza, Basel, Switzerland). In case of all cell lines, the aforementioned basal media were supplemented with 10% fetal bovine serum (FBS, Gibco®/Invitrogen Corporation, New York, NY, USA), l-glutamine (2 mmol/L) (Lonza, Basel, Switzerland) and 100 μg/mL penicillin/streptomycin (Gibco®/Invitrogen Corporation, New York, NY, USA). (Gibco®/Invitrogen Corporation, New York, NY, USA). All cell lines were cultivated under standard conditions (37 °C, humidified 5% CO_2 atmosphere) in plastic culture dishes (Sigma-Aldrich, St. Louis, MO, USA or Eppendorf AG, Hamburg, Germany).

S.2. Viability Assays

S.2.1. Impedance-Based Assay

The cytotoxicity of the compounds on PANC-1 cells was measured using the impedimetry-based xCELLigence SP System (ACEA Biosciences, San Diego, CA, USA). Monitoring the changes of the impedance is a real-time technique to detect the number of adhered cells on an electrode surface and can provide a sensitive way for cytotoxicity studies. The change in the impedance is expressed as in the form of Cell Index (CI). The calculation was evaluated by the software (RTCA 2.0, ACEA Biosciences, San Diego, CA, USA) integrated to xCELLigence System.

For determination of IC_{50} (a concentration that decreases the cell viability by 50%) values, the tested novel polycyclic indole derivatives were solved in DMSO at a concentration of 1×10^{-2} M and further diluted in DMEM medium supplemented with 1% penicillin/streptomycin, 1% glutamine, 10% fetal bovine serum.

At first, the only medium-containing wells were measured which resulted in a stable background signal. Afterwards, the PANC-1 cells (1.0×10^4 cells/well) were seeded into the E-plate 96 PET. In the first 24 h, the PANC-1 cells adhere to the surface, then a plateau phase is settled. In the next step, the cells were treated with a set of dilutions of the test compounds (range of the final concentrations: 1×10^{-5} to 1×10^{-8} M) and the changes in CI were monitored for at least 72 hours at 10 kHz. The control wells were treated with an adequate volume ratio of DMSO (<1v/v%). Each measurement was done in triplicates. The CI values of each concentration obtained at 24, 48 and 72 h after the treatment were normalized to that of the DMSO control. The IC50 value was calculated for these normalized CI values by fitting a sigmoidal dose-response curve with the nonlinear regression function of OriginPro 8 (OriginLab Corporation, Northampton, MA, USA).

***S.2.2.*.** Colorimetric Assay

In case of the COLO-205 and EBC-1 cells, due to their semi-adherent characteristics, a colorimetric probe, the alamarBlue-assay was performed in order to measure the antiproliferative/cytotoxic effects of the polycyclic indole derivatives. The same assay was done on the A2058 cell line, as it fails to establish a stable plateau phase during the impedimetric analysis.

The cells were seeded in a transparent 96-well plate (Sarstedt AG, Nümbrecht, Germany) at 1 x 10^4 cells/well density. Then the test compounds were added (range of the final concentrations: 1×10^{-5} to 1×10^{-8} M), and at 24, 48 and 72 h following the treatment alamarBlue reagent (0.15 mg/mL, Sigma-Aldrich, St. Louis, MO, USA) solved in PBS (phosphate-buffered saline, pH = 7.2) was added to all wells. After a 4-hour-long incubation with the alamarBlue reagent, the fluorescence intensity of the samples was recorded by the Fluoroskan™ FL Microplate Fluorometer and Luminometer (Thermo Scientific, Waltham, MA USA) with the following settings: excitation wavelength = 578 nm and emission wavelength = 597 nm. Three parallels were measured for each measurement. Wells containing adequate volume ratio of DMSO (<1v/v%) served as control. The fluorescence intensity of each sample was expressed as a ratio of the fluorescence of DMSO control. The nonlinear regression function of OriginPro 8 (OriginLab Corporation, Northampton, MA, USA) was used for fitting sigmoidal dose-response curves to the normalized fluorescence intensities in order to calculate the IC_{50} values.

S.3. Copies of NMR spectra of the products

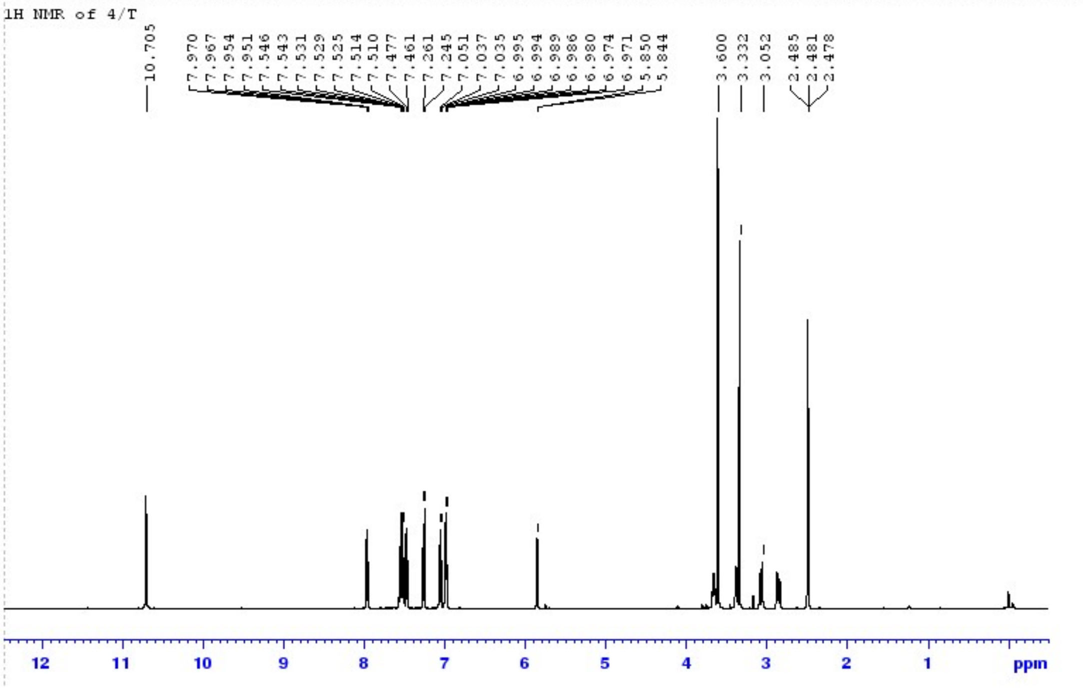

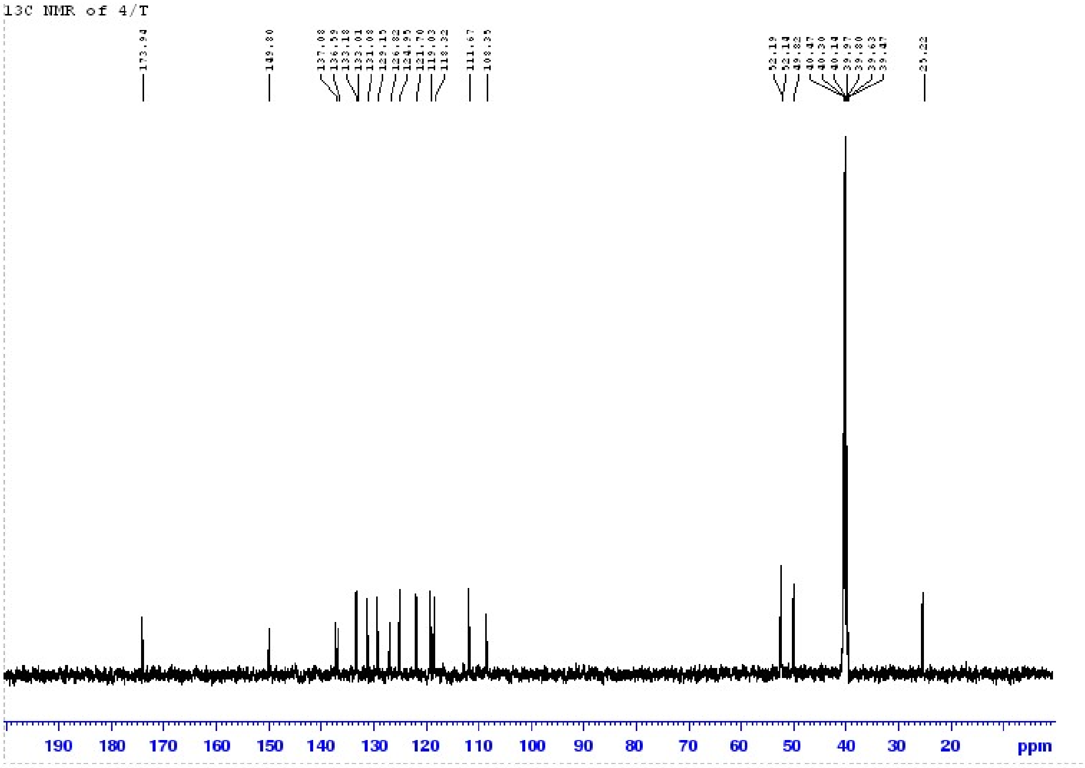

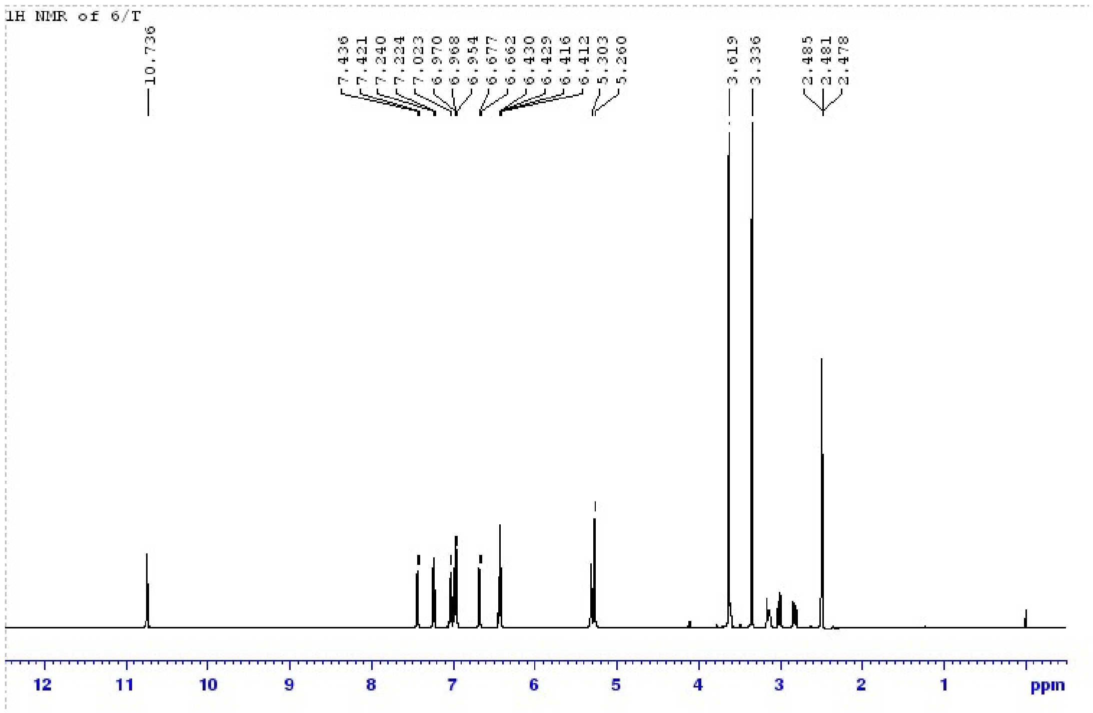
1H NMR of 6/T
10.736
7.436
7.421
7.240
7.224
7.023
6.970
6.968
6.954
6.677
6.662
6.430
6.429
6.416
6.412
5.303
5.260
3.619
3.336
2.485
2.481
2.478
12 11 10 9 8 7 6 5 4 3 2 1 ppm

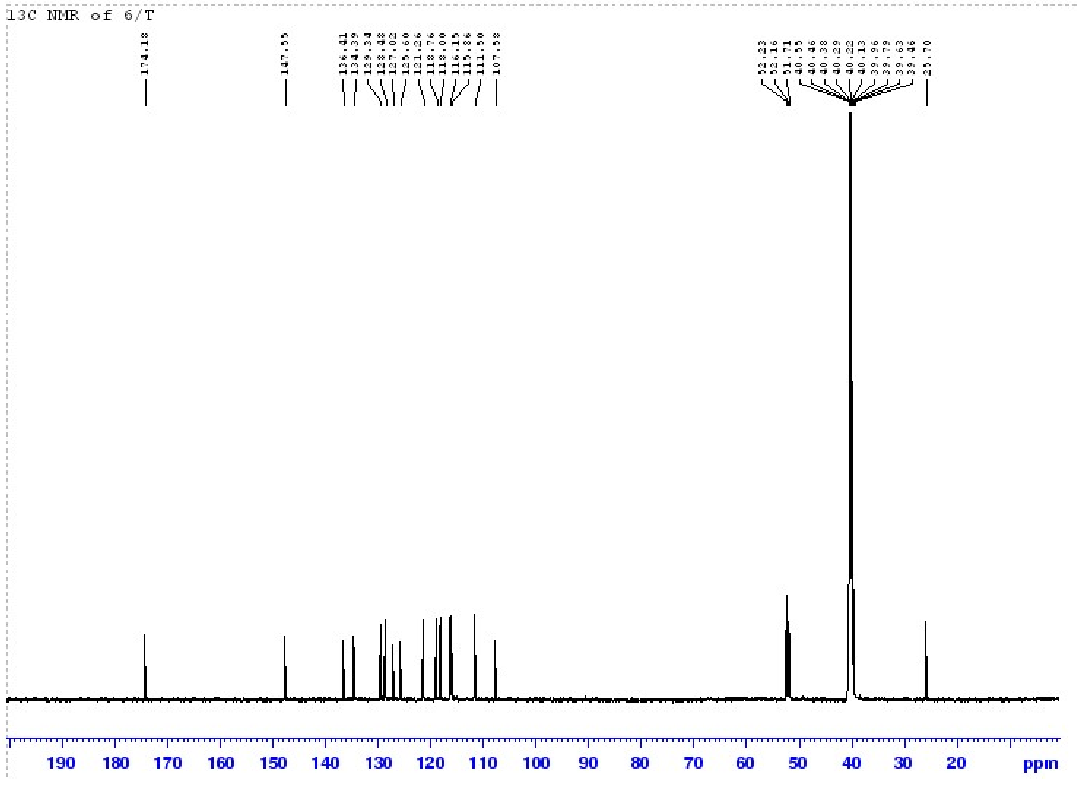
13C NMR of 6/T
174.18
147.55
136.41
134.39
129.34
128.48
127.02
125.60
121.26
118.76
118.00
116.15
115.86
111.50
107.58
52.23
52.16
51.71
40.55
40.46
40.38
40.29
40.22
40.13
39.96
39.79
39.63
39.46
25.70
190 180 170 160 150 140 130 120 110 100 90 80 70 60 50 40 30 20 ppm

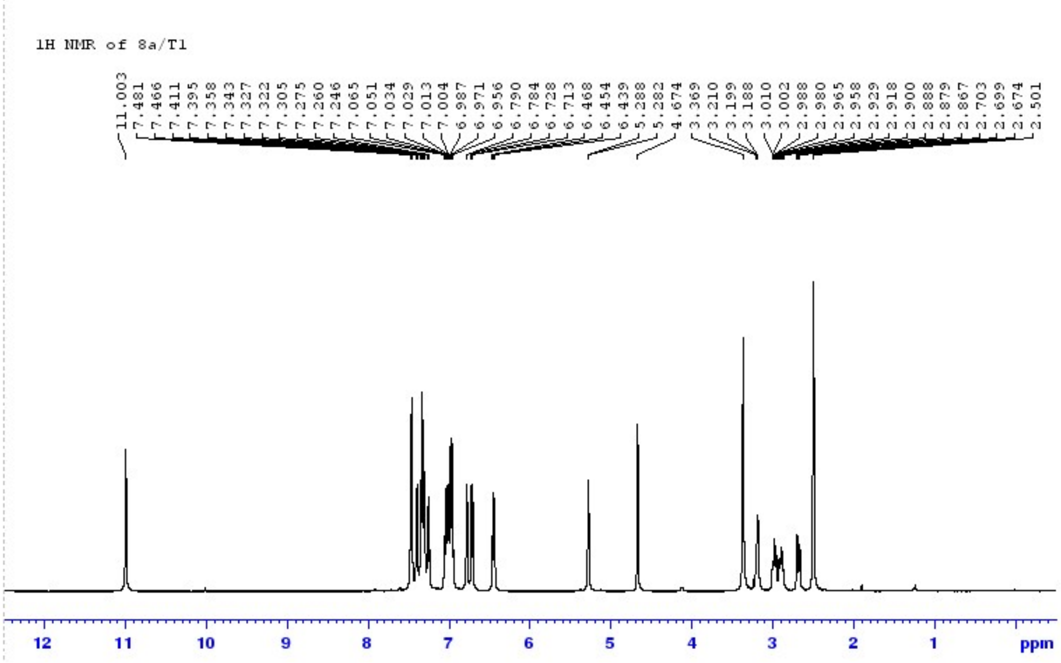
1H NMR of 8a/T1
ppm

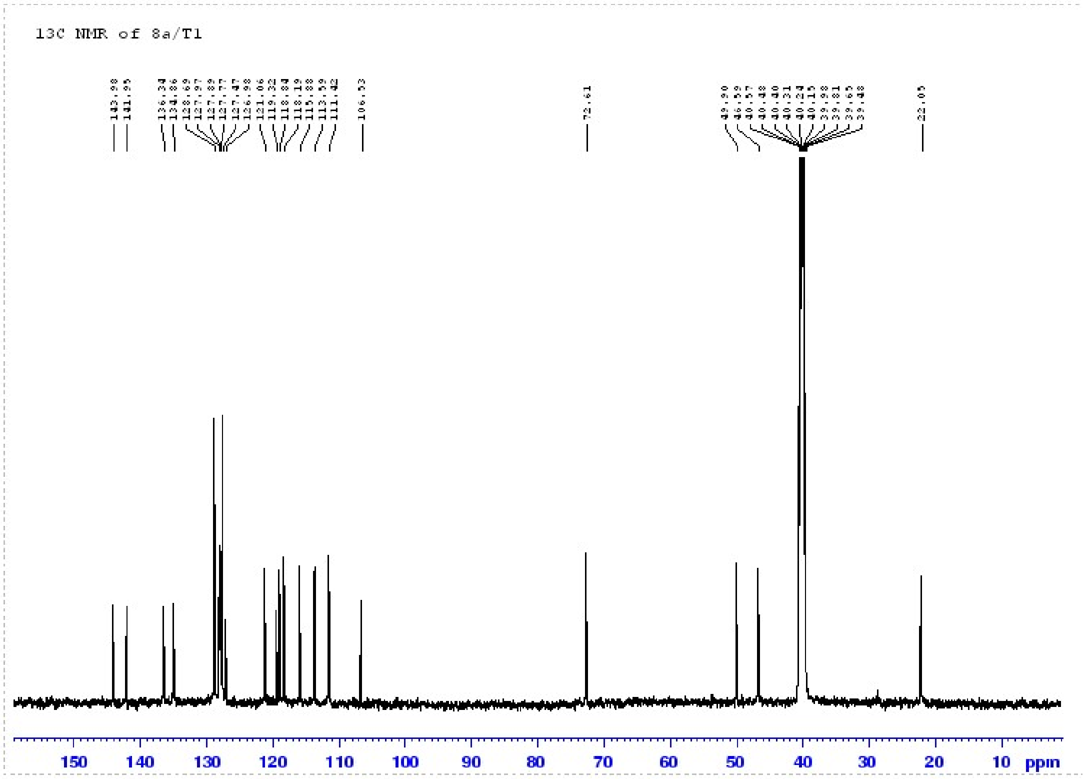
13C NMR of 8a/T1
ppm

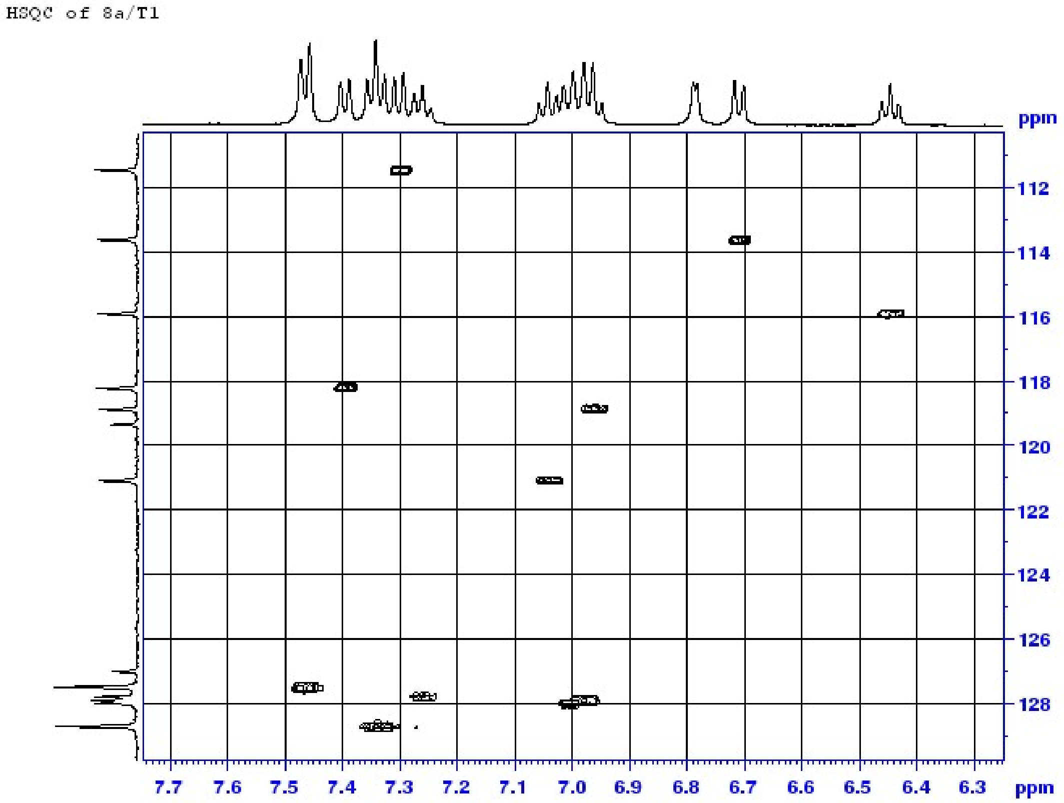

(aromatic region in high resolution)

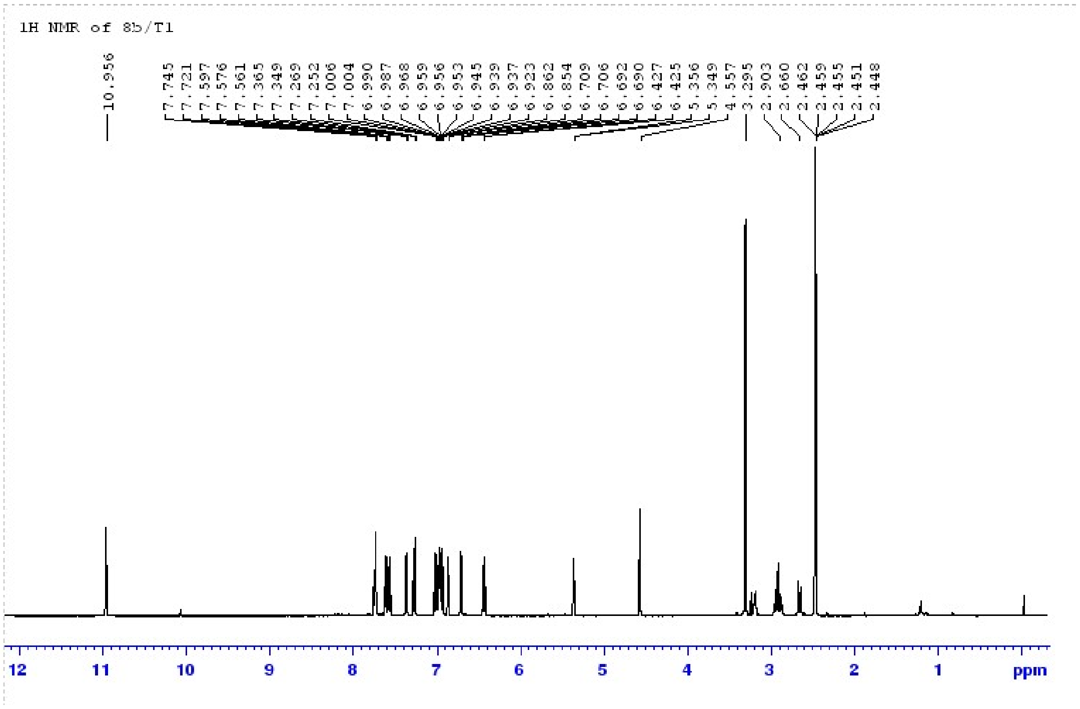

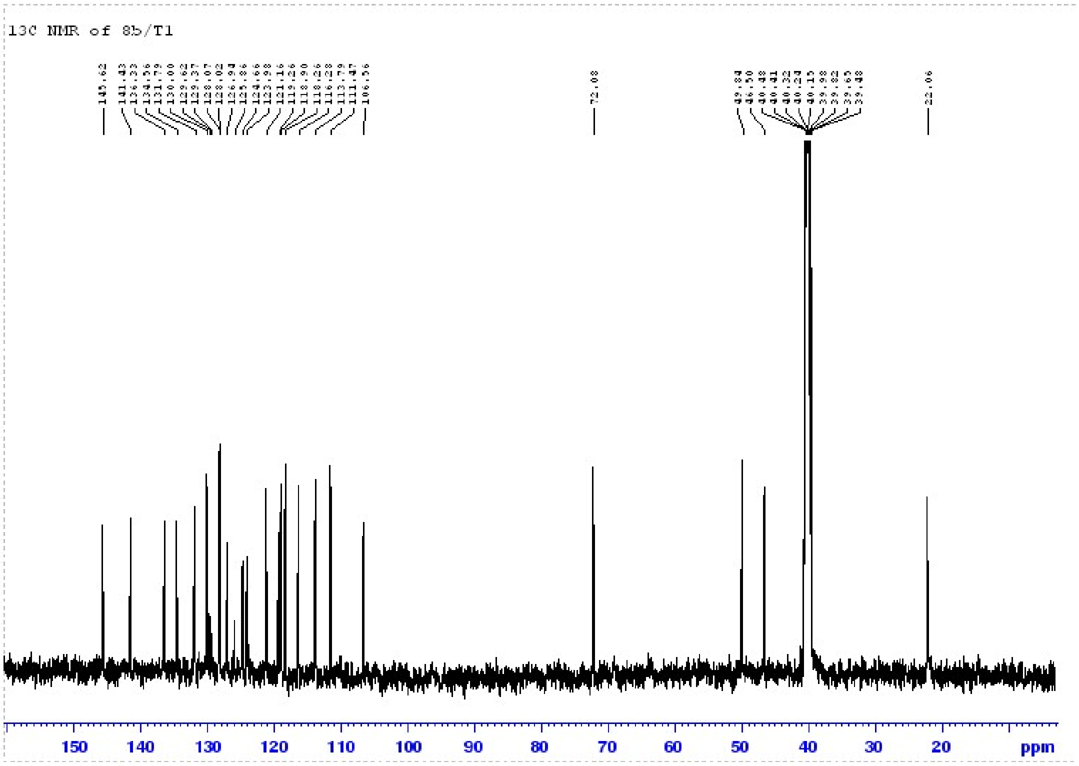

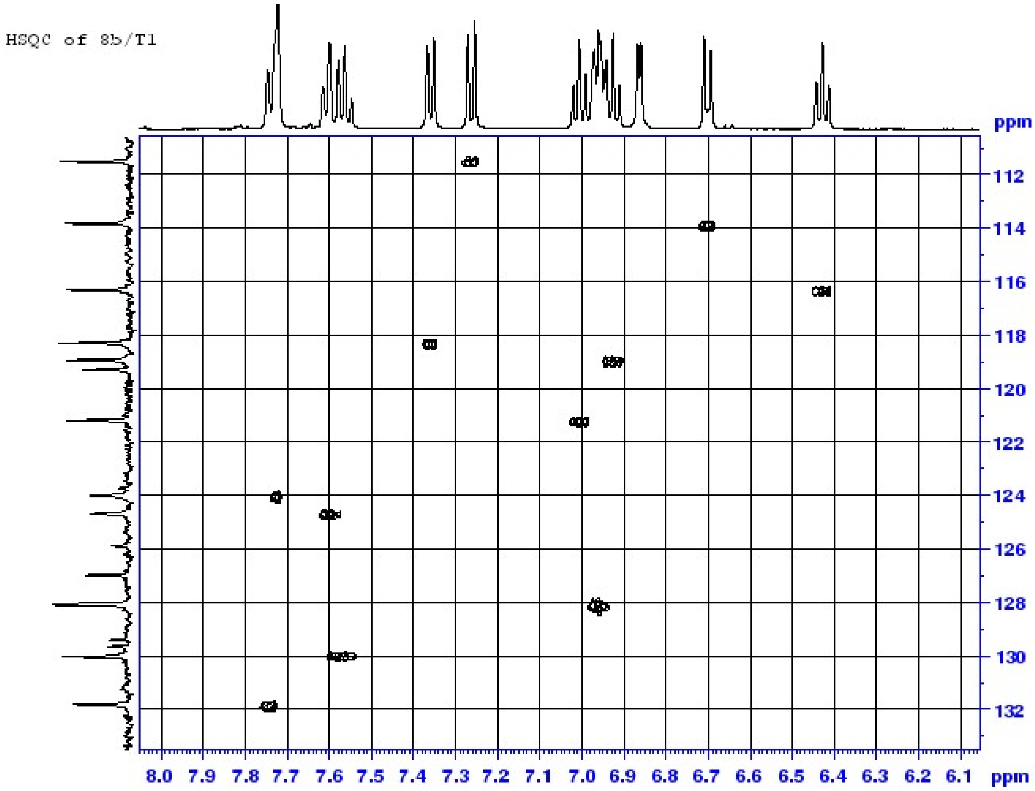

(aromatic region in high resolution)

HMBC of 8b/T1

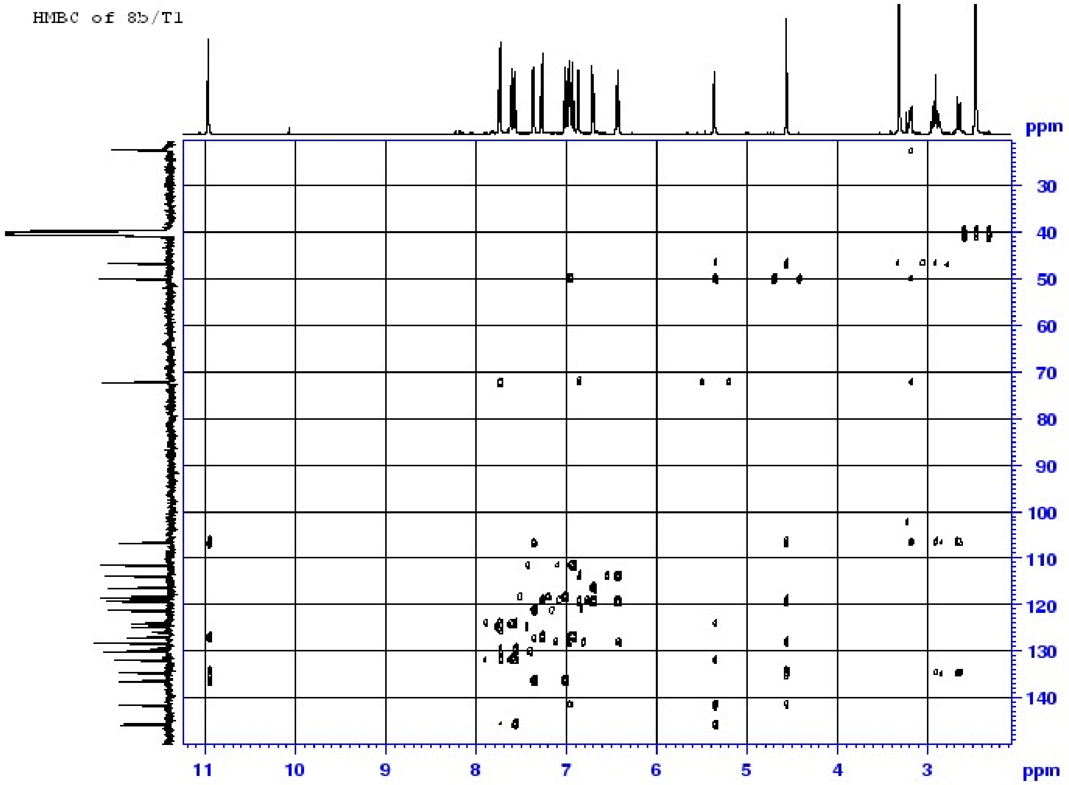

NOESY of 8b/T1

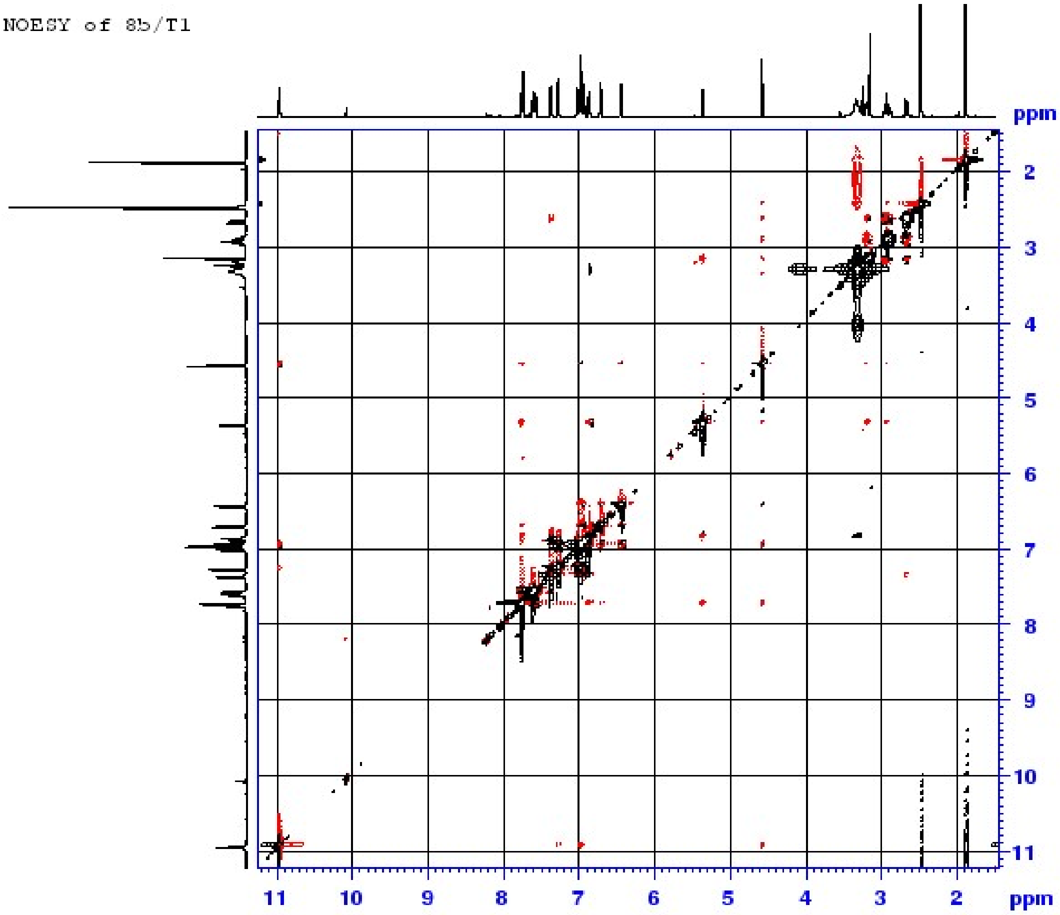

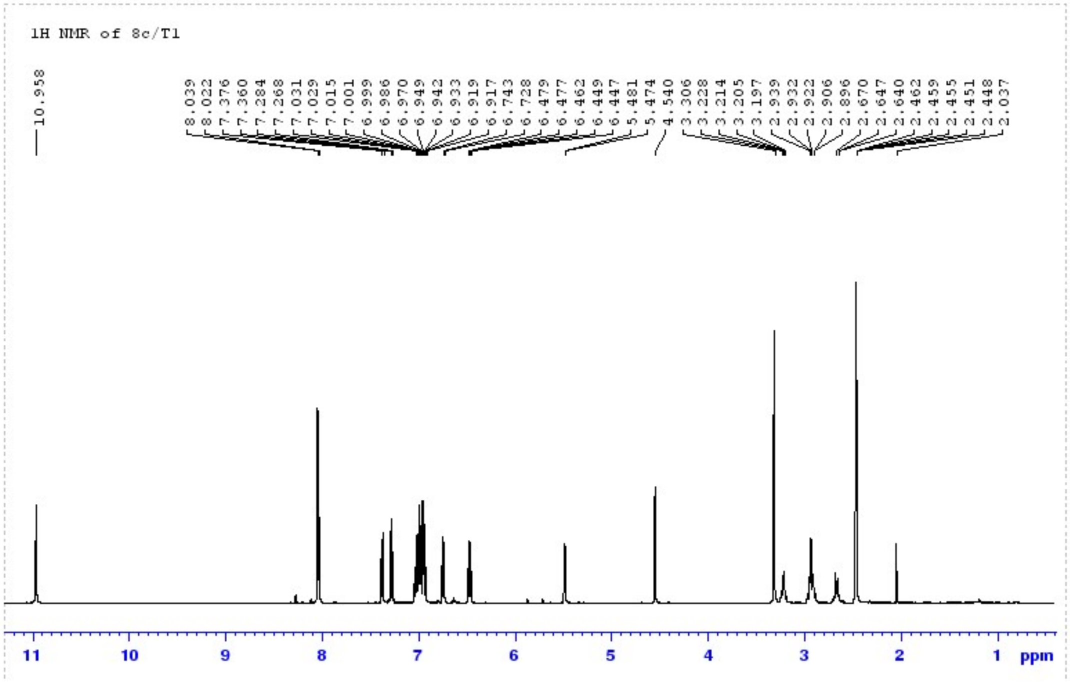
1H NMR of 8c/T1
ppm

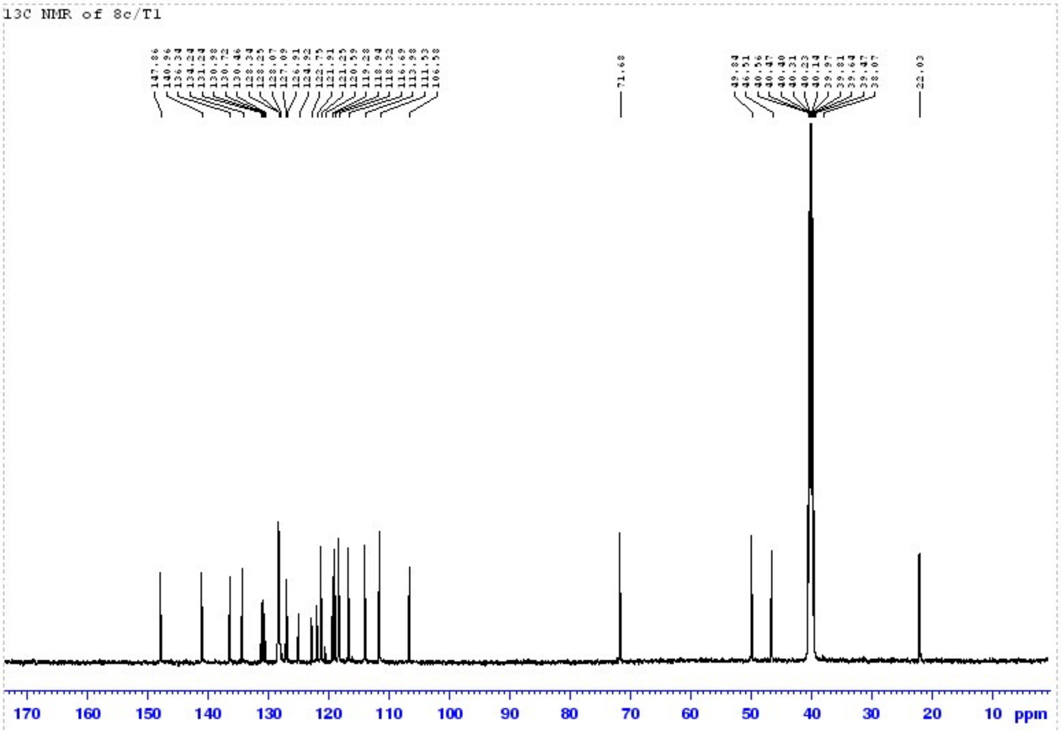
13C NMR of 8c/T1
ppm

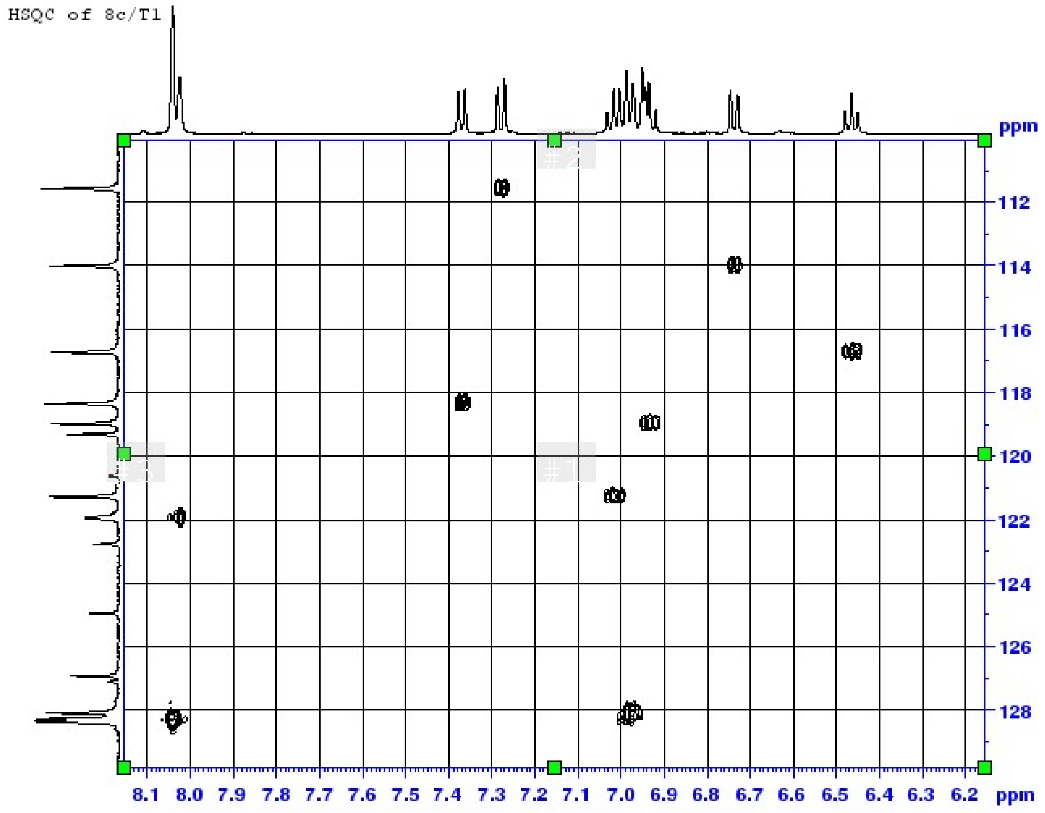

(aromatic region in high resolution)

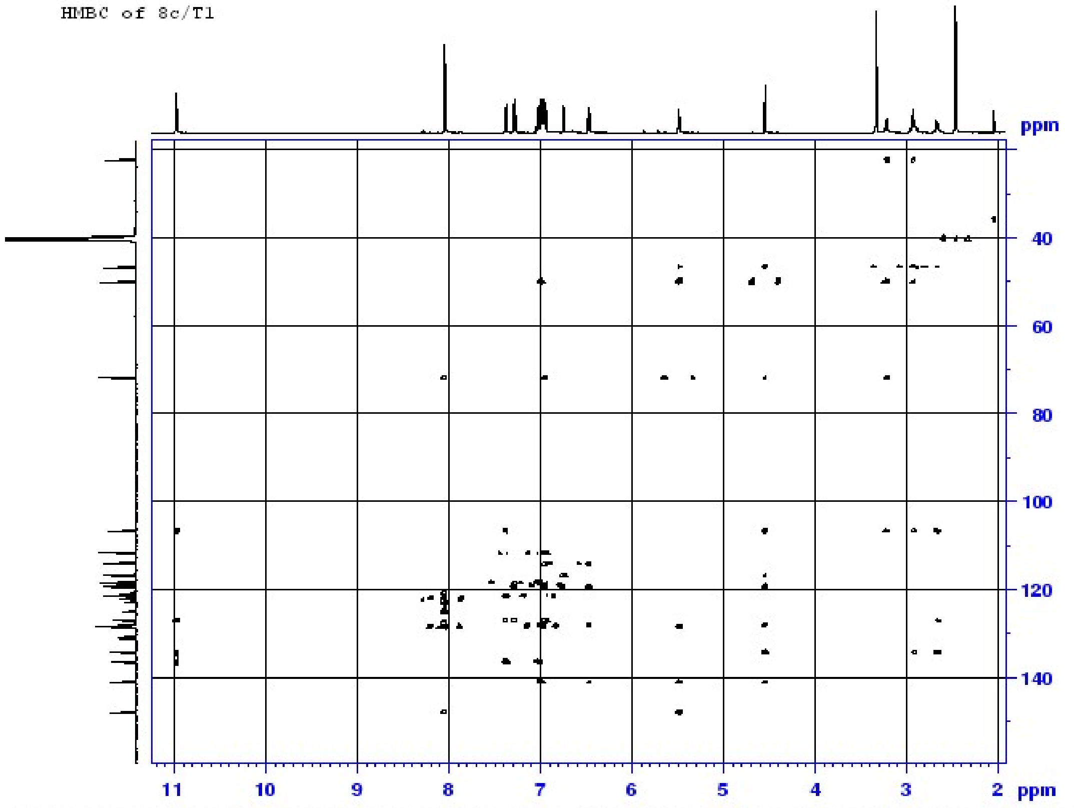

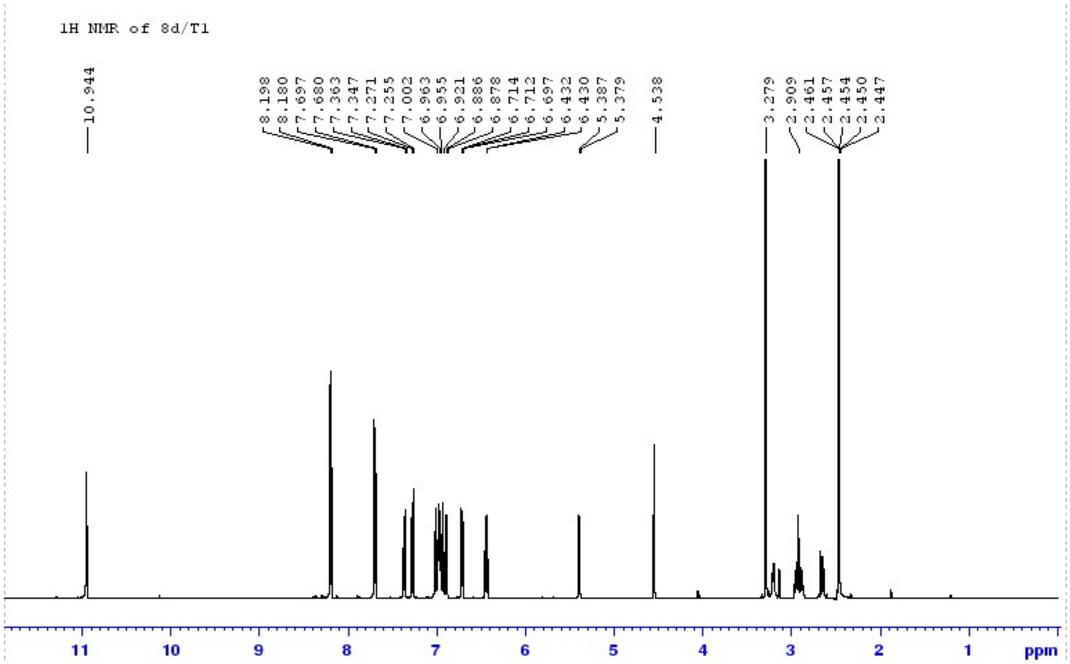
1H NMR of 8d/T1
10.944
8.198
8.180
7.697
7.680
7.363
7.347
7.271
7.255
7.002
6.963
6.955
6.921
6.886
6.878
6.714
6.712
6.697
6.432
6.430
5.387
5.379
4.538
3.279
2.909
2.461
2.457
2.454
2.450
2.447
11 10 9 8 7 6 5 4 3 2 1 ppm

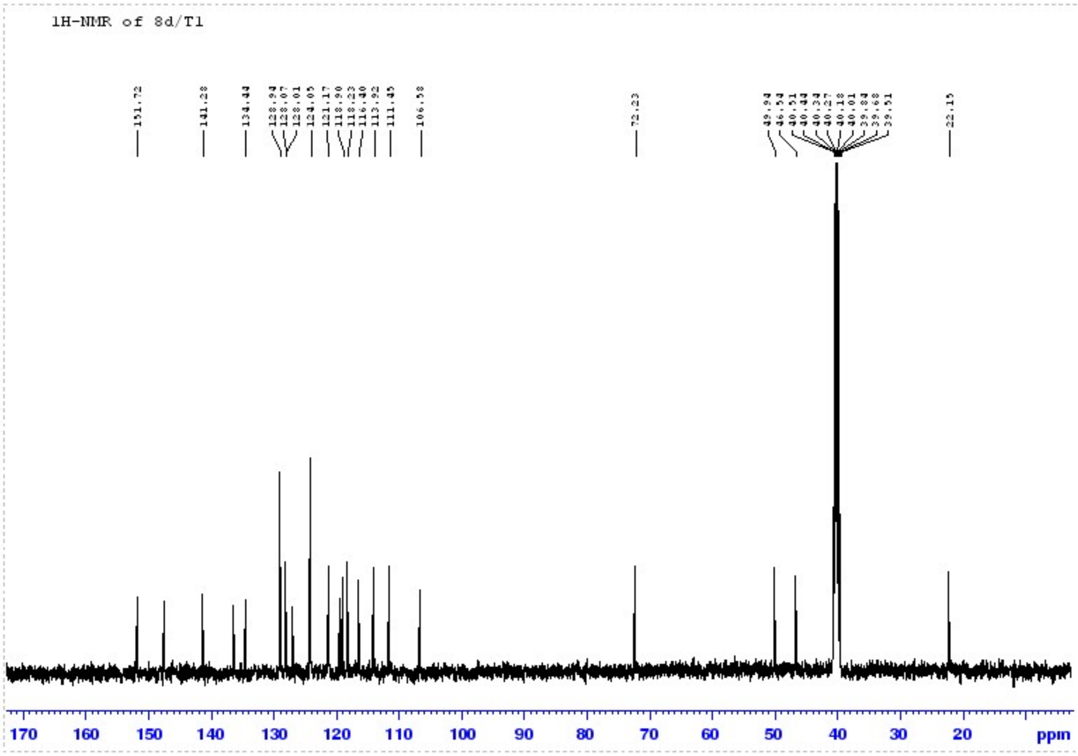
1H-NMR of 8d/T1
151.72
141.28
134.44
128.94
128.07
128.01
124.05
121.17
118.90
118.23
116.40
113.92
111.45
106.58
72.23
49.94
46.54
40.51
40.44
40.34
40.27
40.18
40.01
39.84
39.68
39.51
22.15
170 160 150 140 130 120 110 100 90 80 70 60 50 40 30 20 ppm

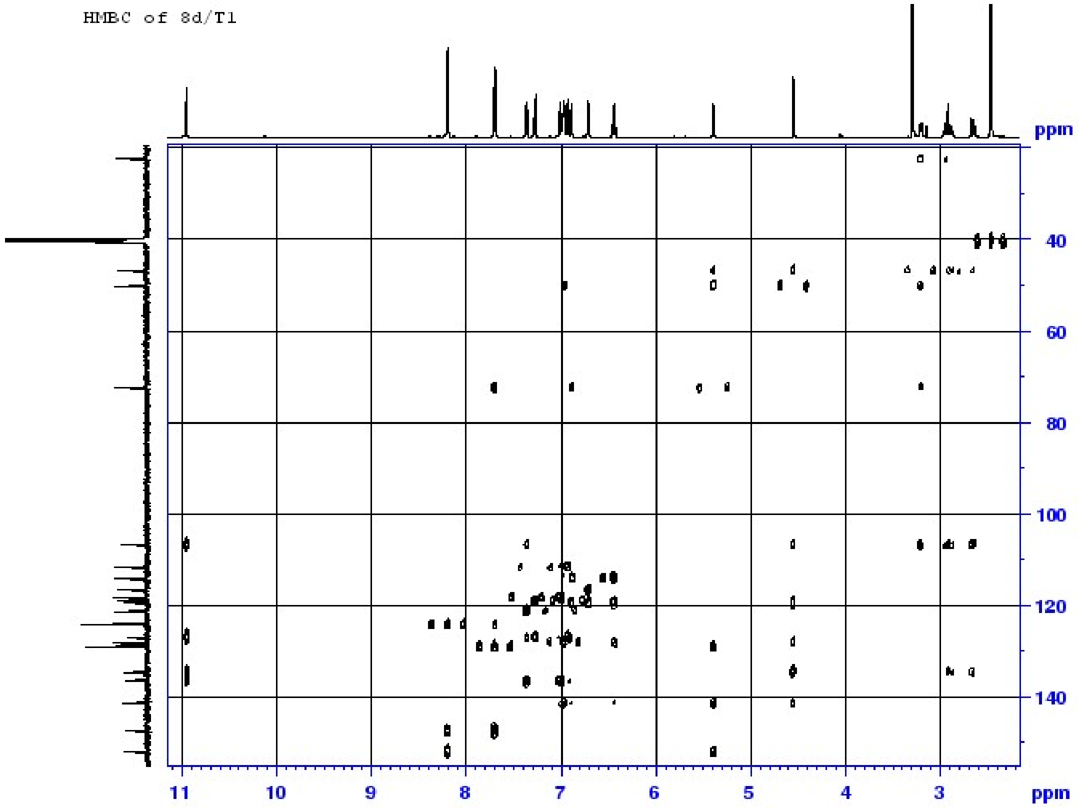
HMBC of 8d/T1
ppm
40
60
80
100
120
140
11
10
9
8
7
6
5
4
3
ppm

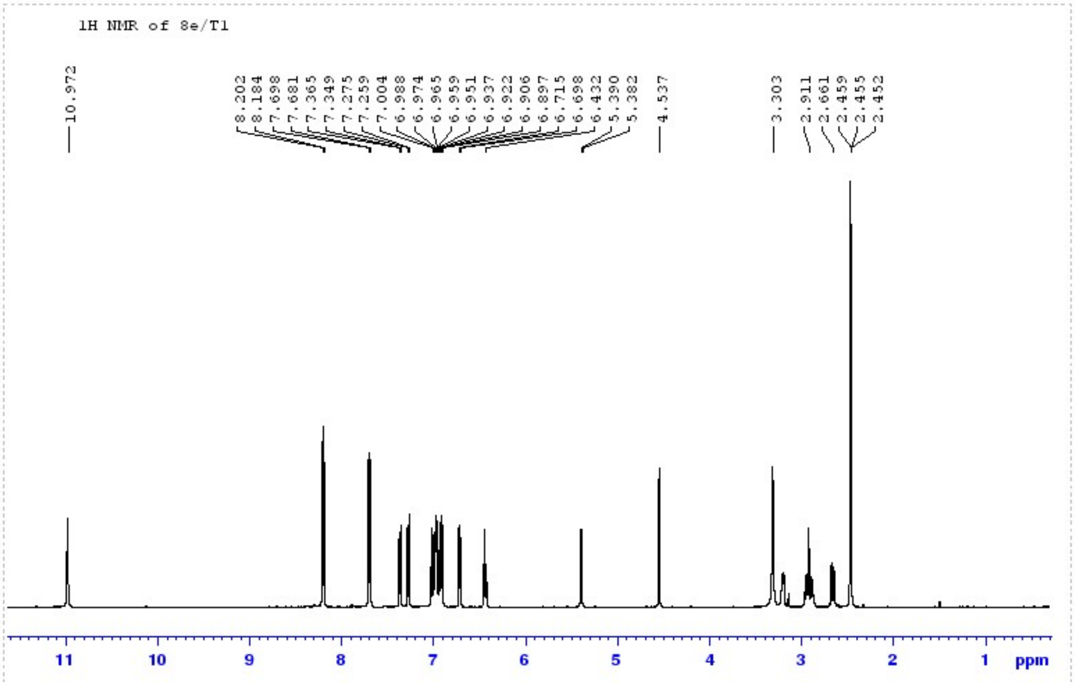
1H NMR of 8e/T1
10.972
8.202
8.184
7.698
7.681
7.365
7.349
7.275
7.259
7.004
6.988
6.974
6.965
6.959
6.951
6.937
6.922
6.906
6.897
6.715
6.698
6.432
5.390
5.382
4.537
3.303
2.911
2.661
2.459
2.455
2.452
11
10
9
8
7
6
5
4
3
2
1
ppm

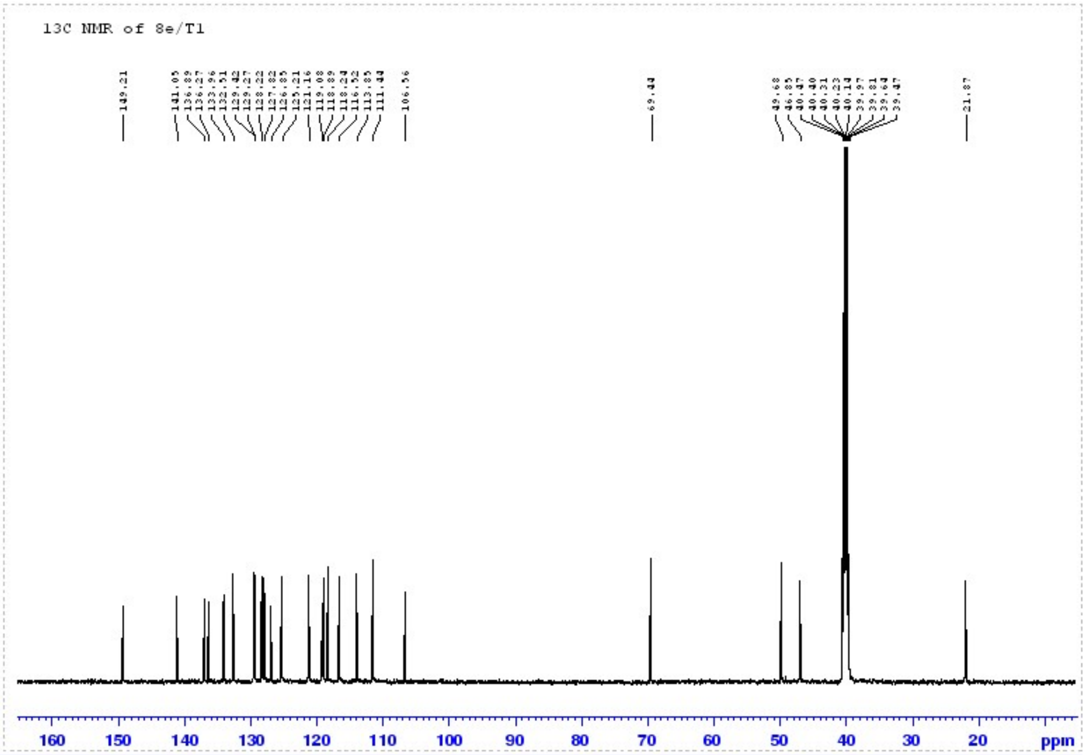

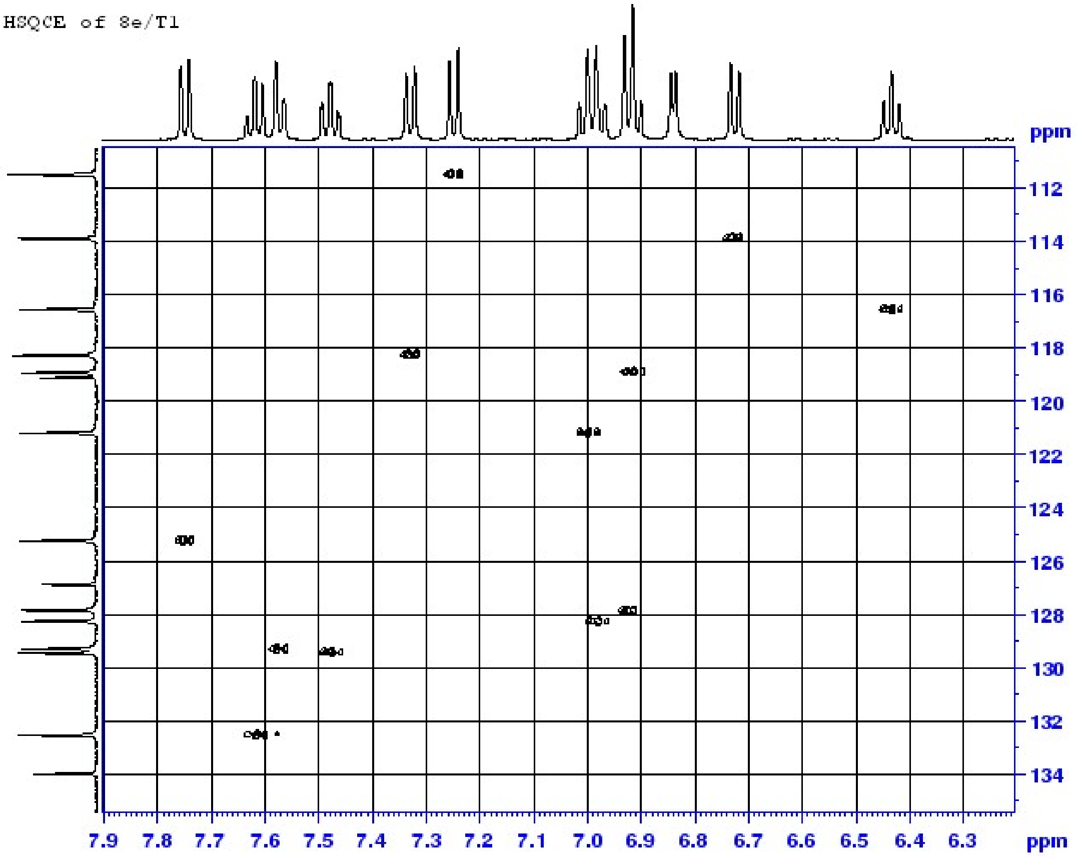

(aromatic region in high resolution)

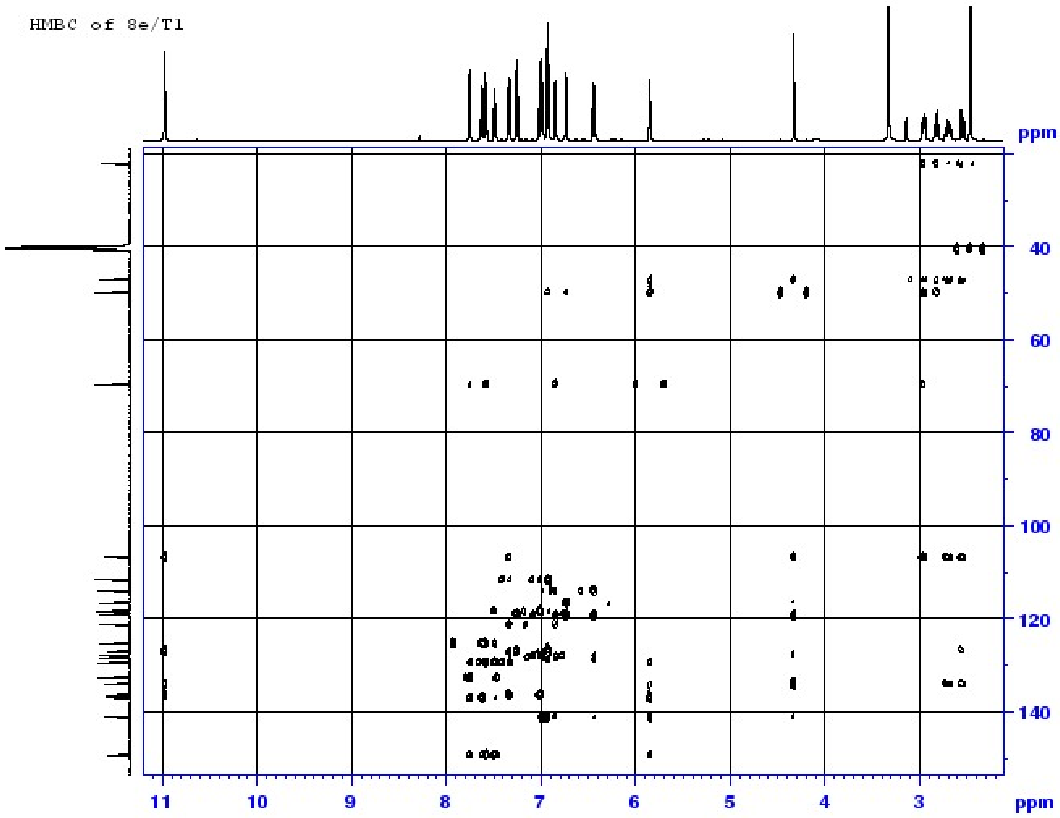
HMBC of 8e/T1
ppm
40
60
80
100
120
140
11
10
9
8
7
6
5
4
3
ppm

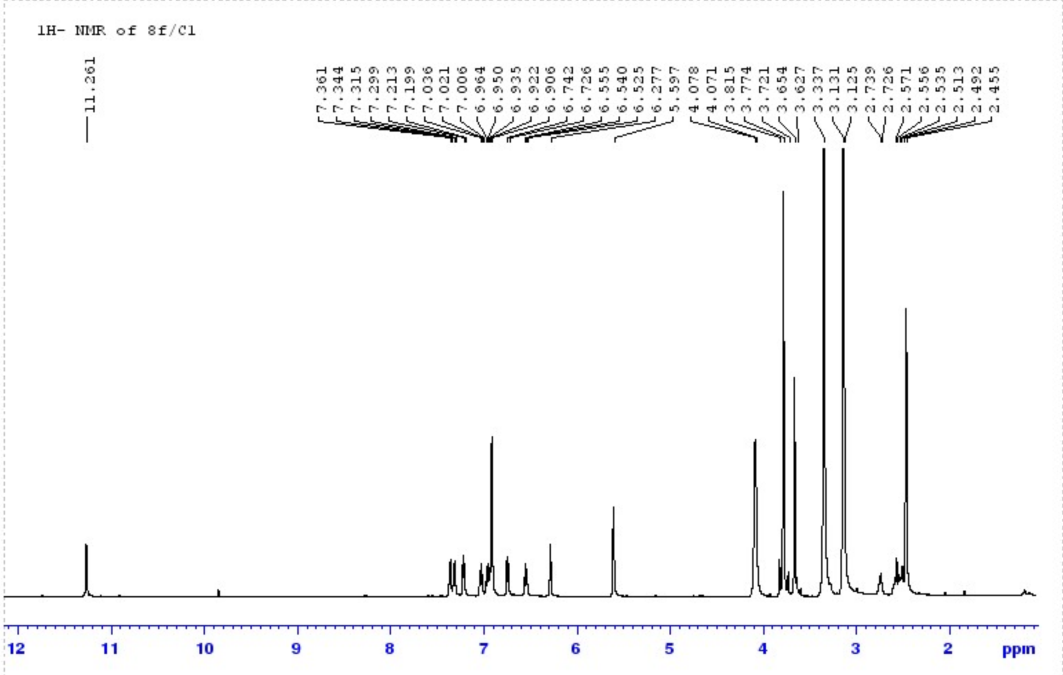
1H- NMR of 8f/C1
11.261
7.361
7.344
7.315
7.299
7.213
7.199
7.036
7.021
7.006
6.964
6.950
6.935
6.922
6.906
6.742
6.726
6.555
6.540
6.525
6.277
5.597
4.078
4.071
3.815
3.774
3.721
3.654
3.627
3.337
3.331
3.131
3.125
2.739
2.726
2.571
2.556
2.535
2.513
2.492
2.455
12
11
10
9
8
7
6
5
4
3
2
ppm

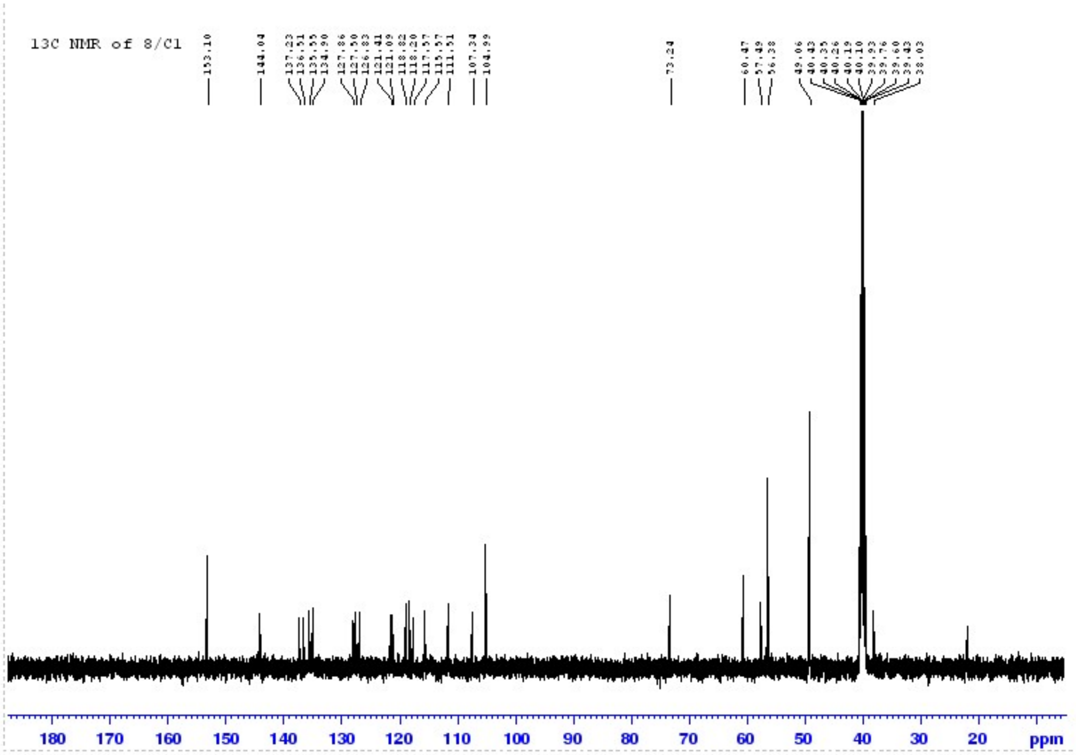
13C NMR of 8/C1
153.10
144.04
137.23
136.51
135.55
134.90
127.86
127.50
126.83
121.41
121.09
118.82
118.20
117.57
115.57
111.51
107.34
104.99
73.24
60.47
57.49
56.38
49.06
40.43
40.35
40.26
40.19
40.10
39.93
39.76
39.60
39.43
38.03
180 170 160 150 140 130 120 110 100 90 80 70 60 50 40 30 20 ppm

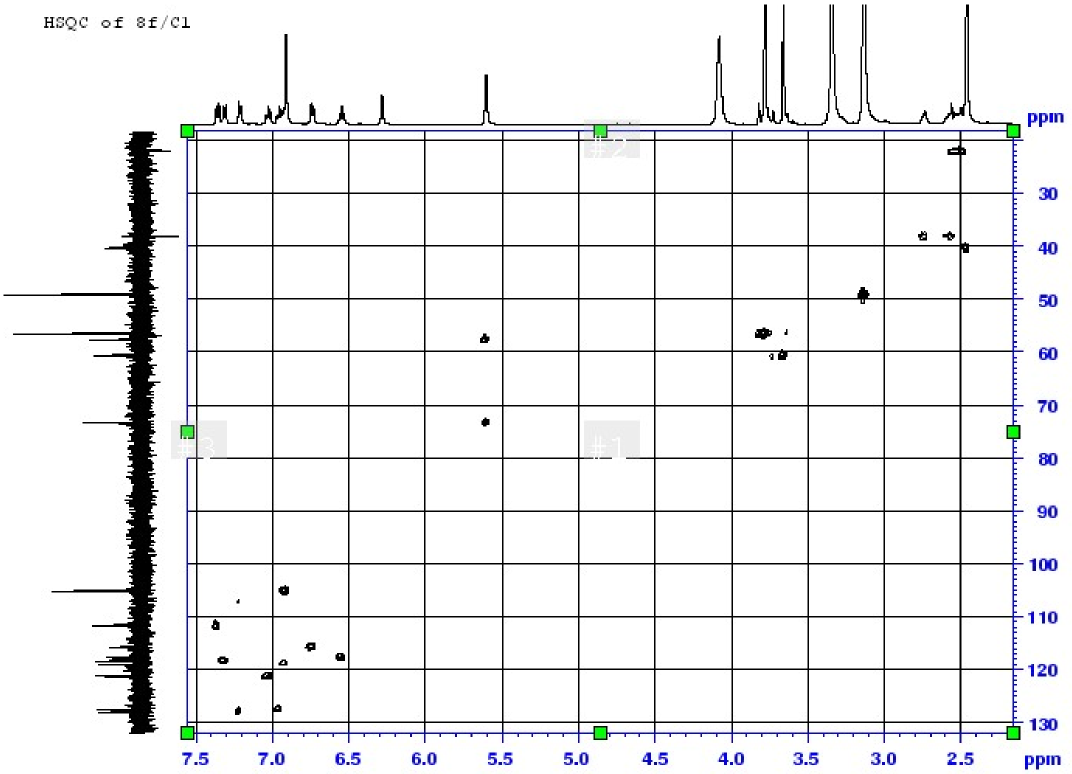
HSQC of 8f/C1
ppm
30 40 50 60 70 80 90 100 110 120 130
7.5 7.0 6.5 6.0 5.5 5.0 4.5 4.0 3.5 3.0 2.5 ppm

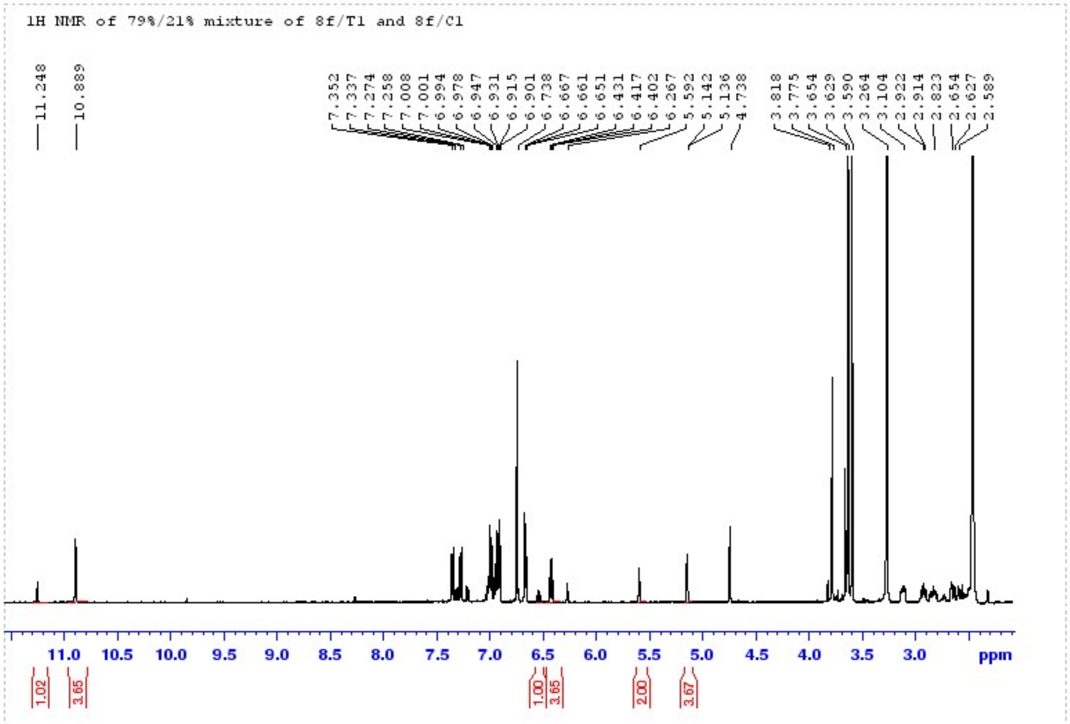
1H NMR of 79%/21% mixture of 8f/T1 and 8f/C1
11.248
10.889
7.352 7.337 7.274 7.258 7.008 7.001 6.994 6.978 6.947 6.931 6.915 6.901 6.738 6.667 6.661 6.651 6.431 6.417 6.402 6.267 5.592 5.142 5.136 4.738
3.818 3.775 3.654 3.629 3.590 3.264 3.104 2.922 2.914 2.823 2.654 2.627 2.589
11.0 10.5 10.0 9.5 9.0 8.5 8.0 7.5 7.0 6.5 6.0 5.5 5.0 4.5 4.0 3.5 3.0 ppm
1.02
3.65
1.00
3.65
2.00
3.67

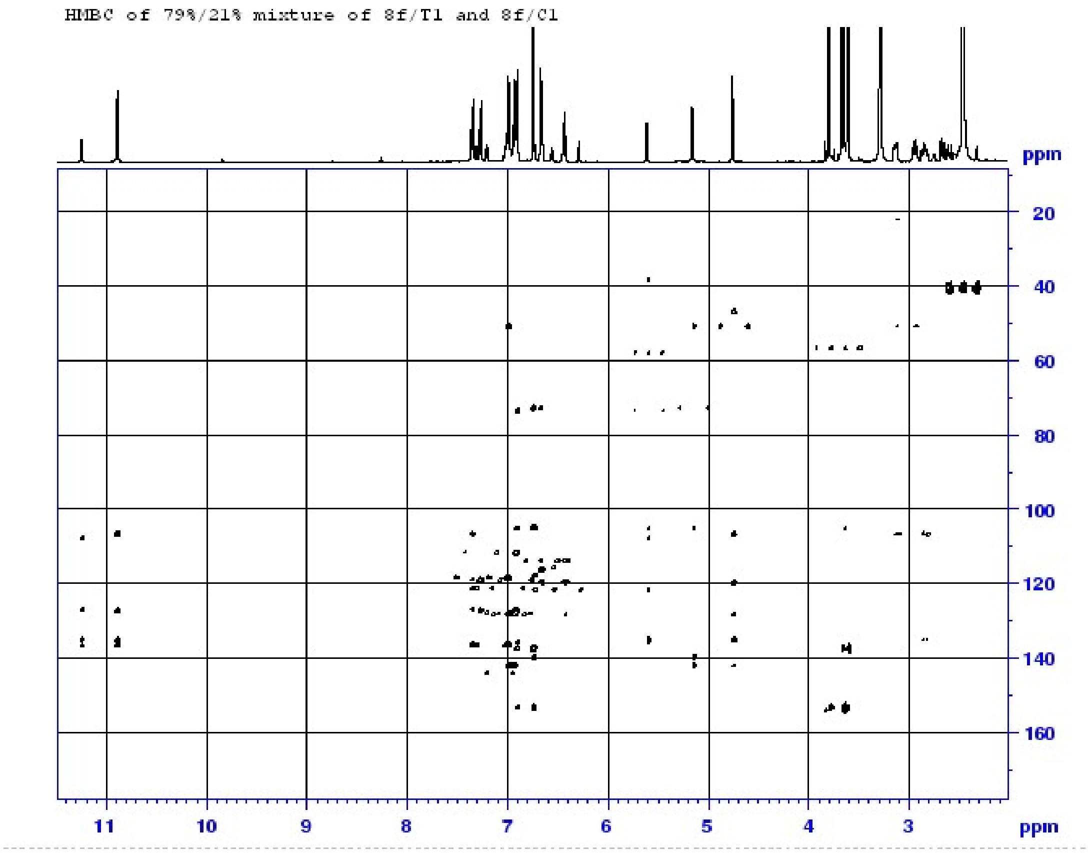
HMBC of 79%/21% mixture of 8f/T1 and 8f/C1
ppm
20
40
60
80
100
120
140
160
11 10 9 8 7 6 5 4 3
ppm

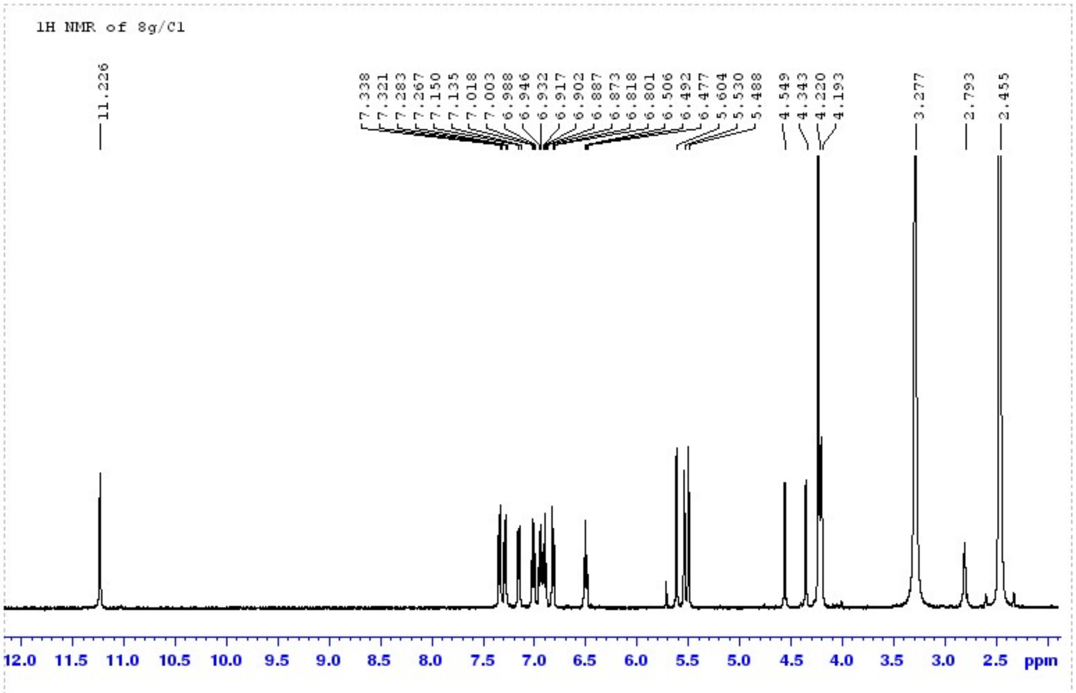
1H NMR of 8g/Cl

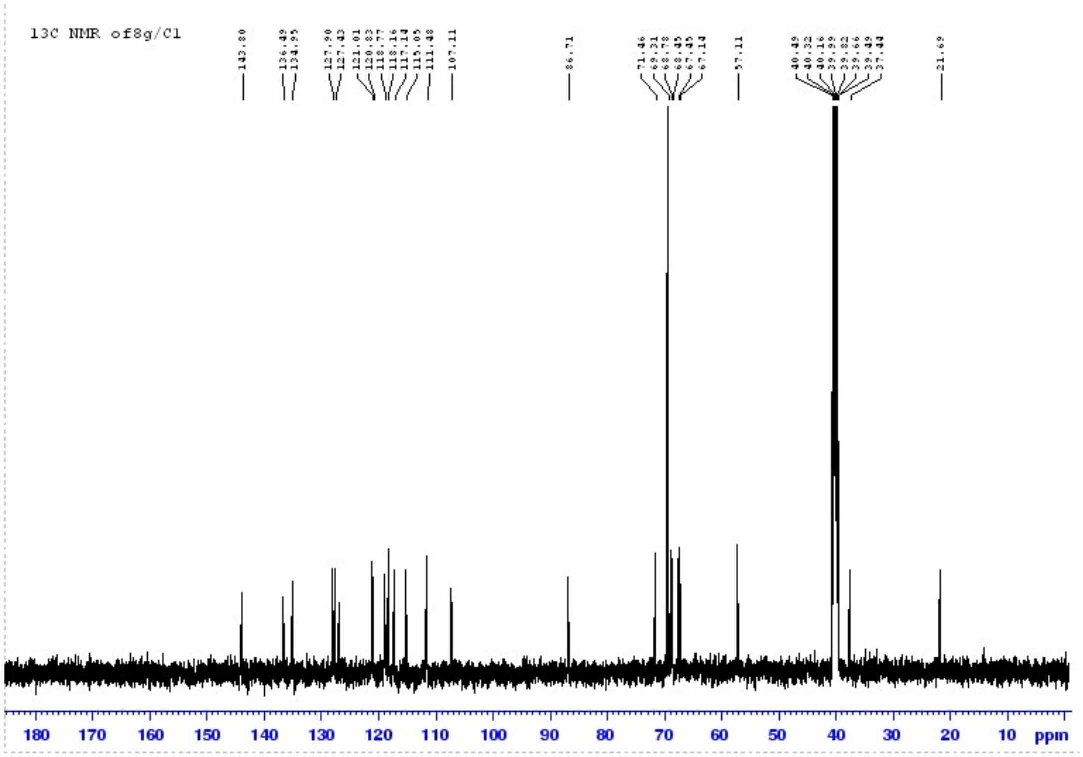
13C NMR of8g/Cl

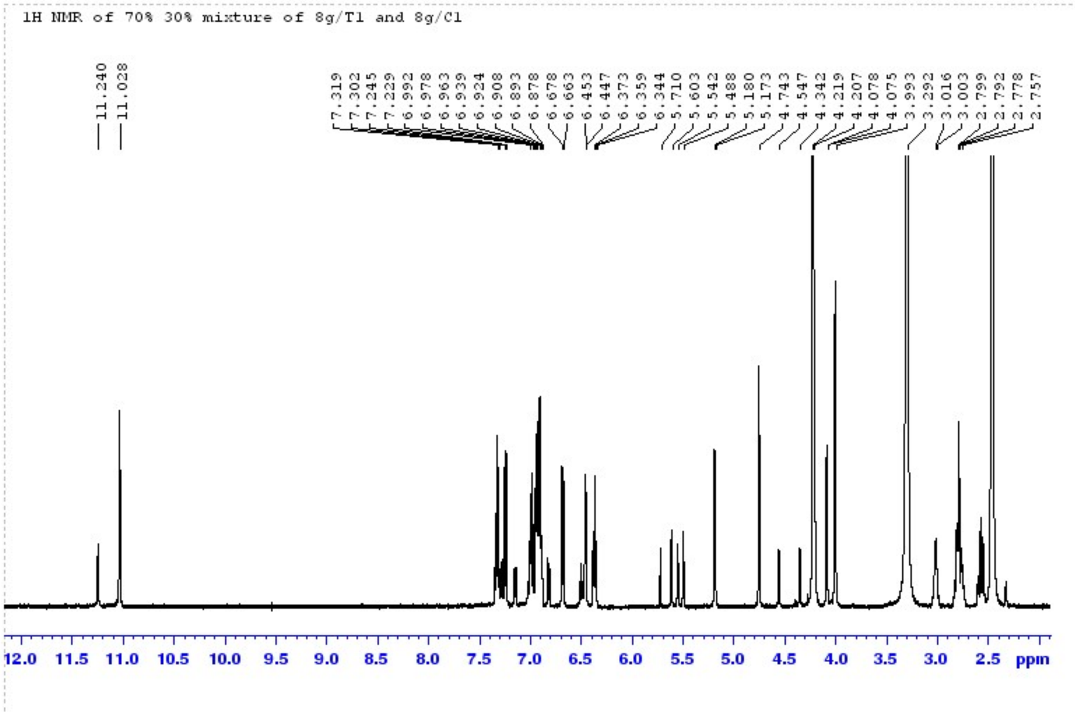
1H NMR of 70% 30% mixture of 8g/T1 and 8g/C1
ppm

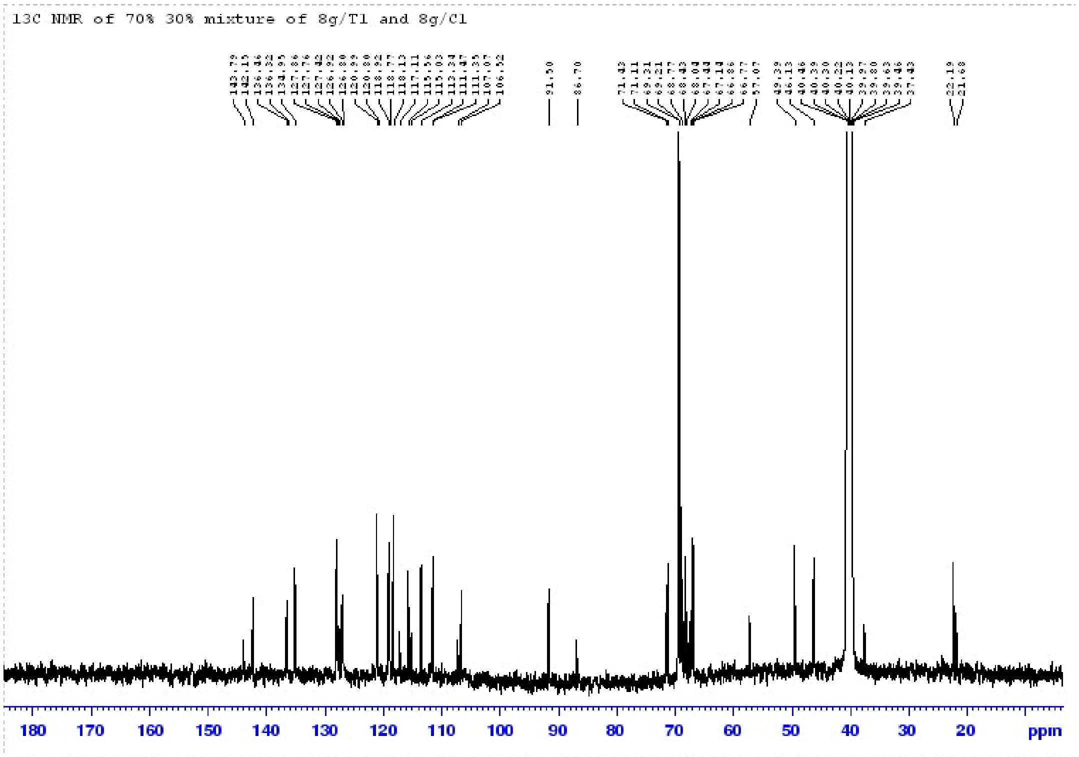
13C NMR of 70% 30% mixture of 8g/T1 and 8g/C1
ppm

HSQC of 70% 30% mixture of 8g/T1 and 8g/C1

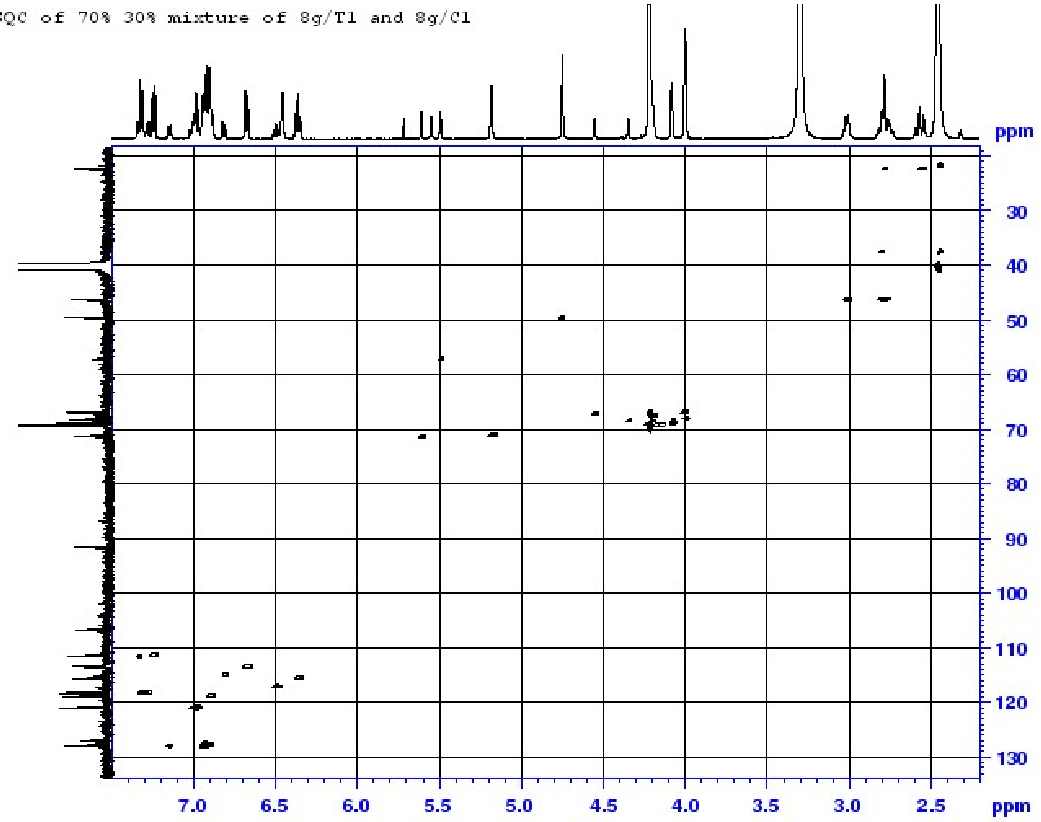

COSY of 705 30% mixture of 8g/T1 and 8g/C1

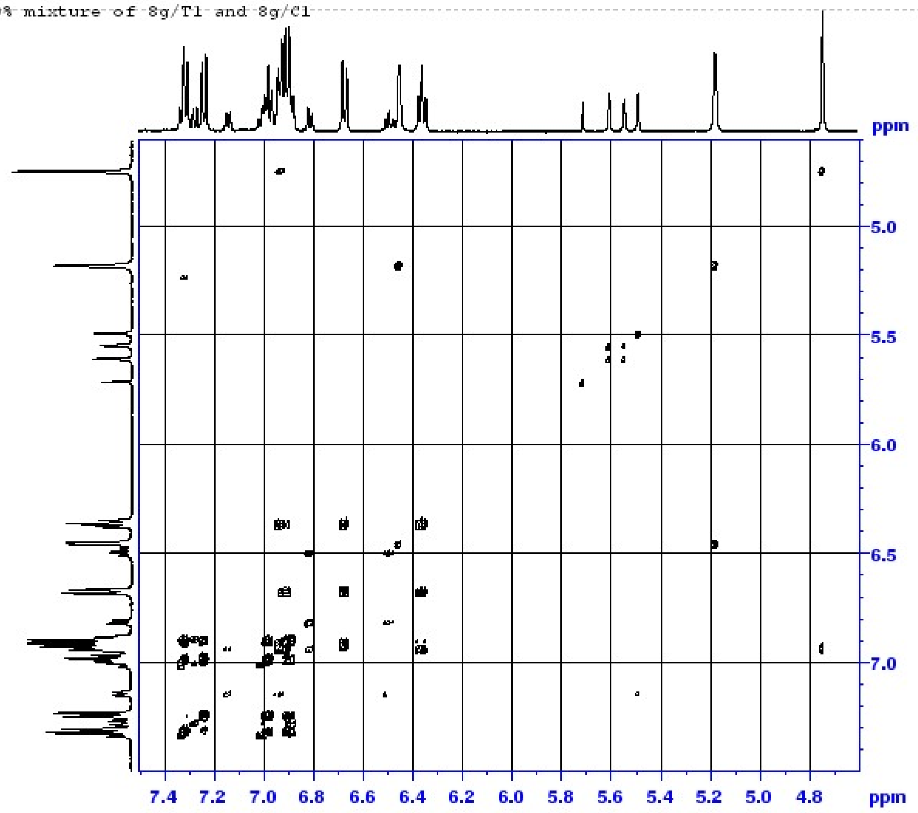

NOESY of 705 30% mixture of 8g/T1 and 8g/C1

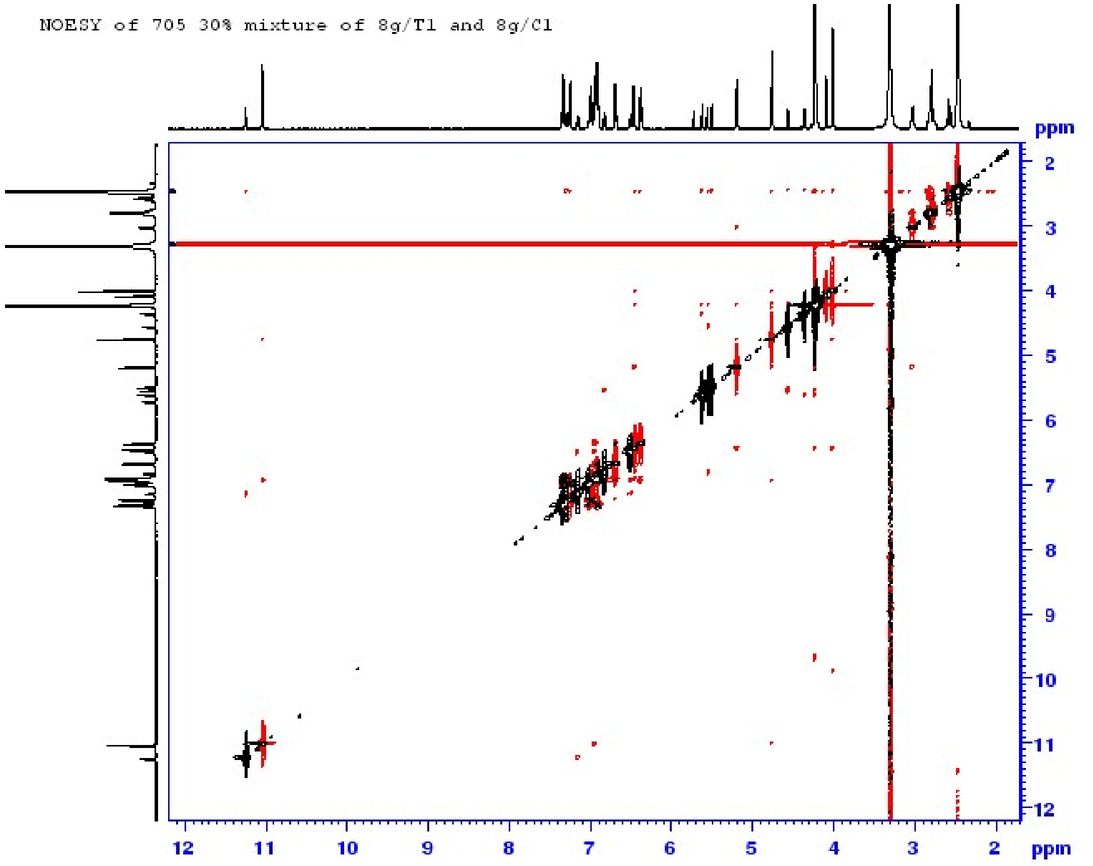

1H NMR of 43%-57% mixture of 8h/T1 and 8h/C1

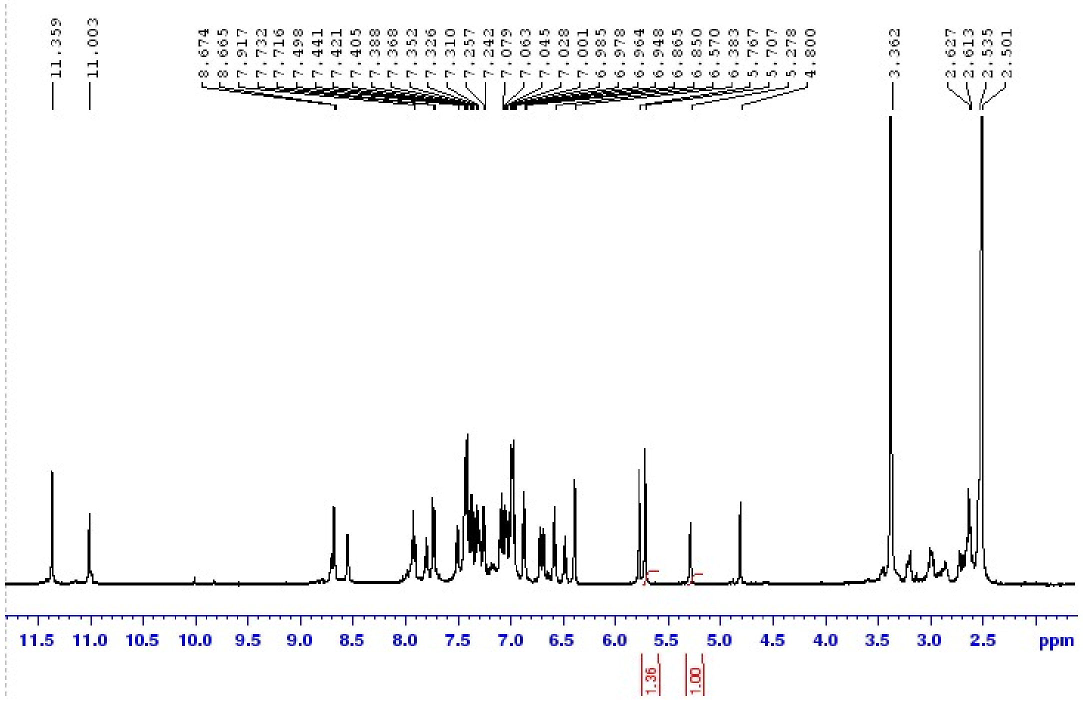

13C NMR of of 43%-57% mixture of 8h/T1 and 8h/C1

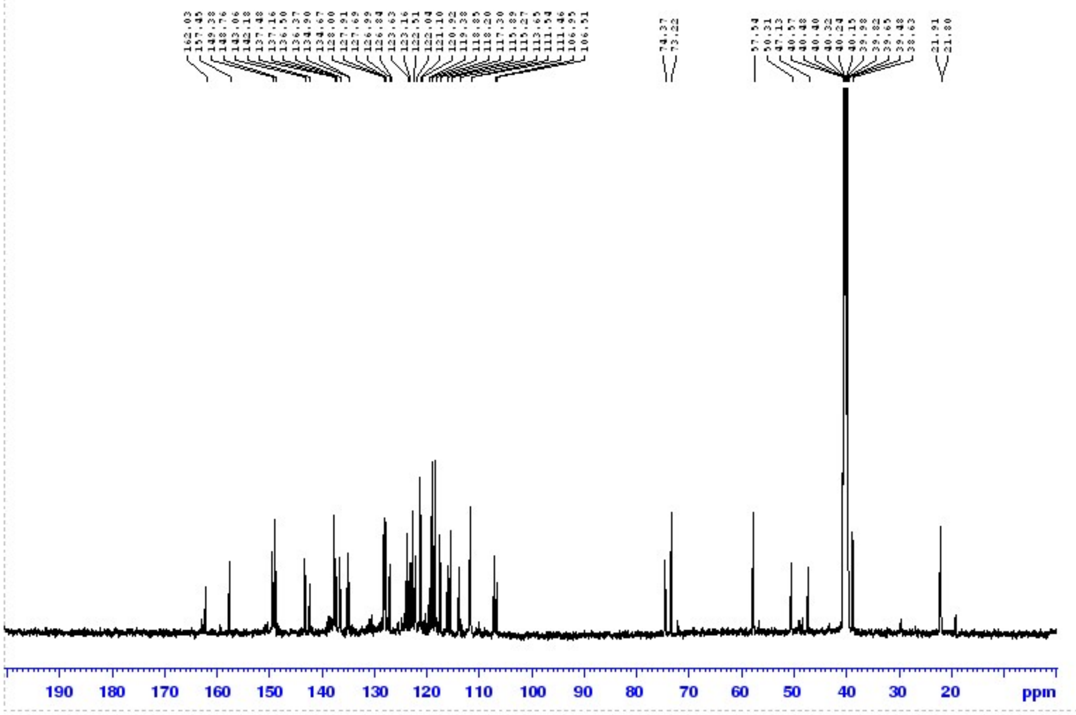

HSQC of 43%-57% mixture of 8h/T1 and 8h/C1

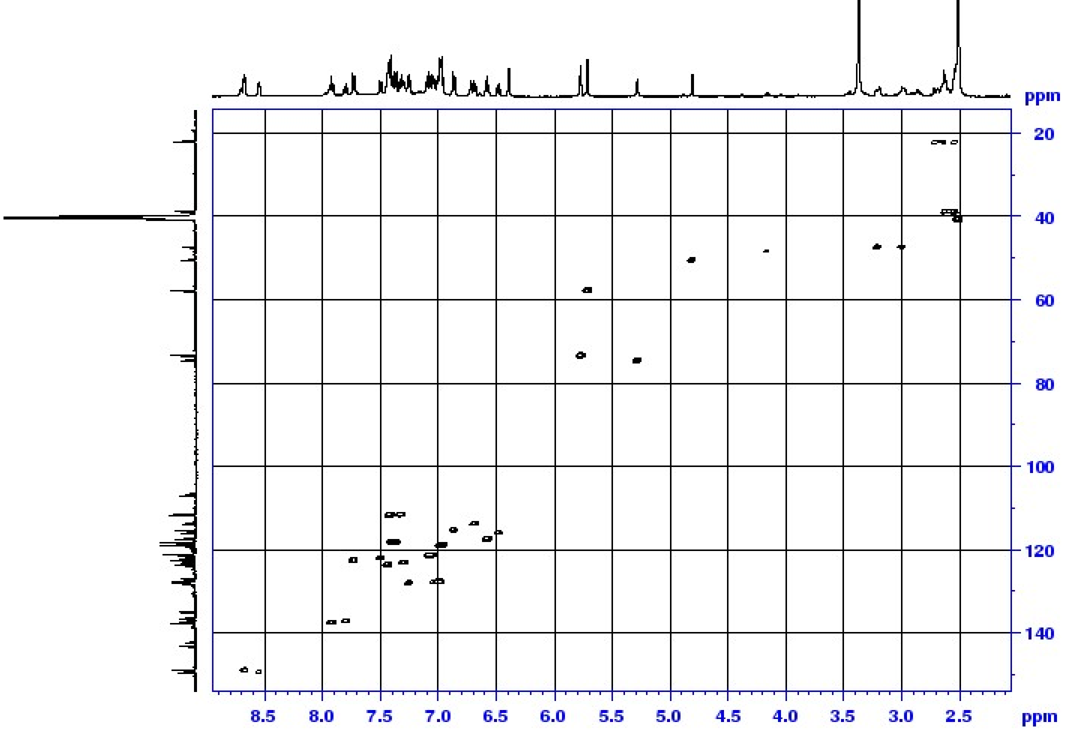

1H NMR of 50%-50% mixture of 8i/T1 and 8i/C1

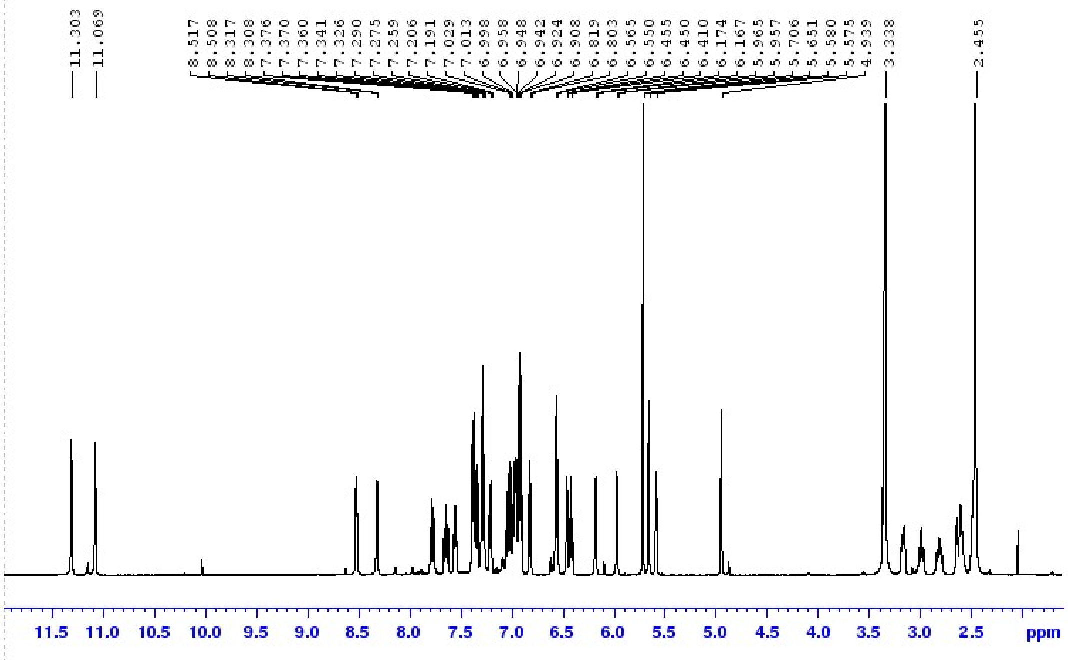

13CNMR of 50%-50% mixture of 8i/T1 and 8i/C1

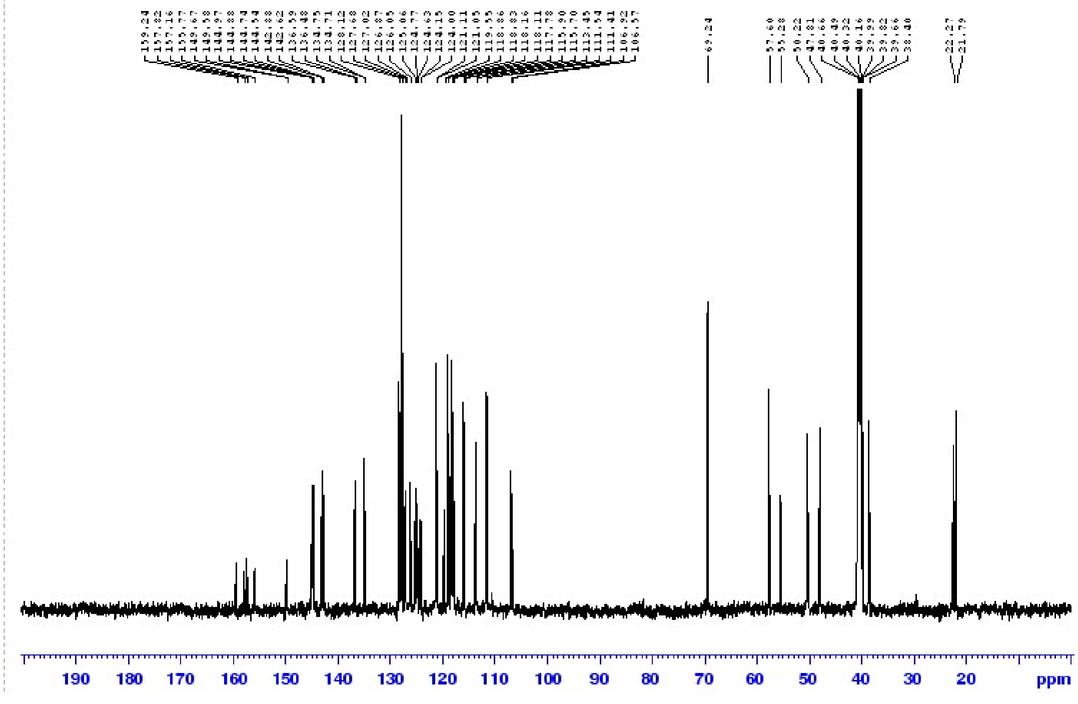

HMBC of 50%-50% mixture of 8i/T1 and 8i/C1

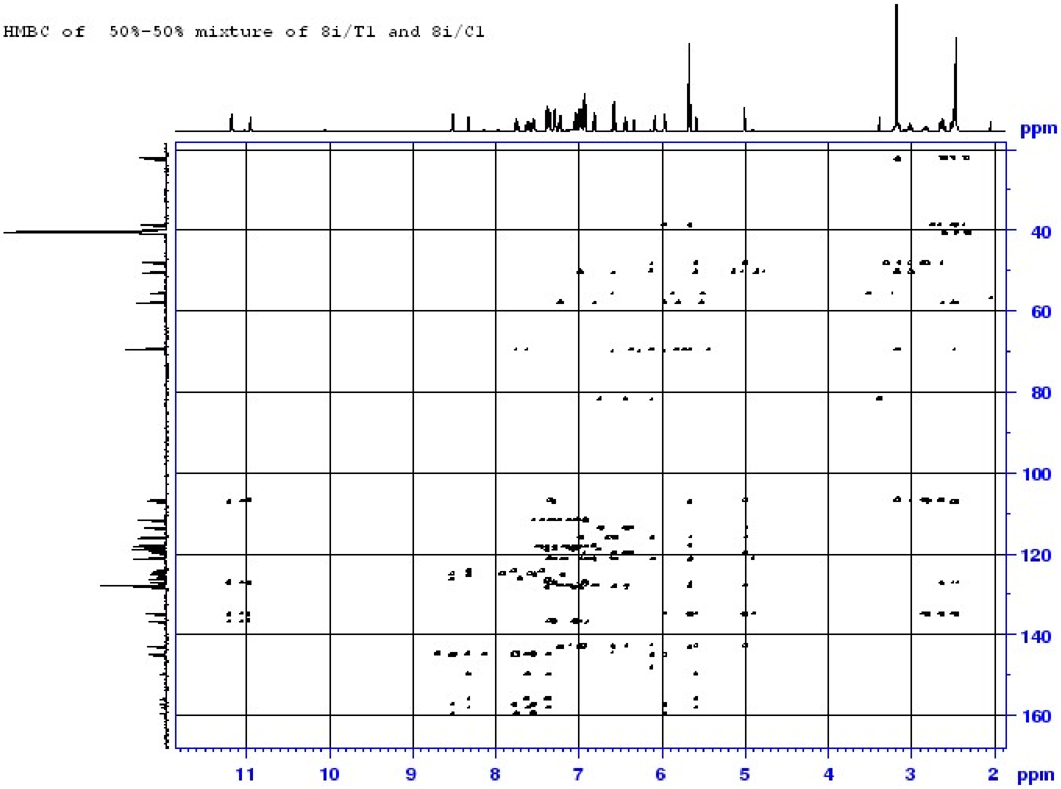

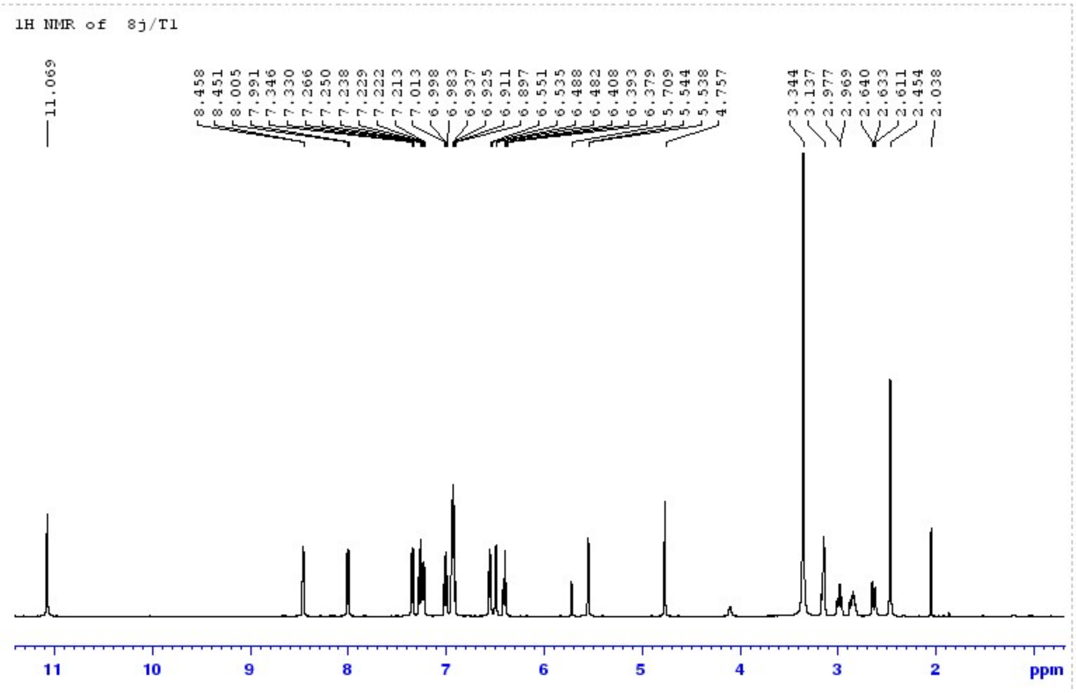
1H NMR of 8j/T1
11.069
8.458
8.451
8.005
7.991
7.346
7.330
7.266
7.250
7.238
7.229
7.222
7.213
7.013
6.998
6.983
6.937
6.925
6.911
6.897
6.551
6.535
6.488
6.482
6.408
6.393
6.379
5.709
5.544
5.538
4.757
3.344
3.137
2.977
2.969
2.640
2.633
2.611
2.454
2.038
11
10
9
8
7
6
5
4
3
2
ppm

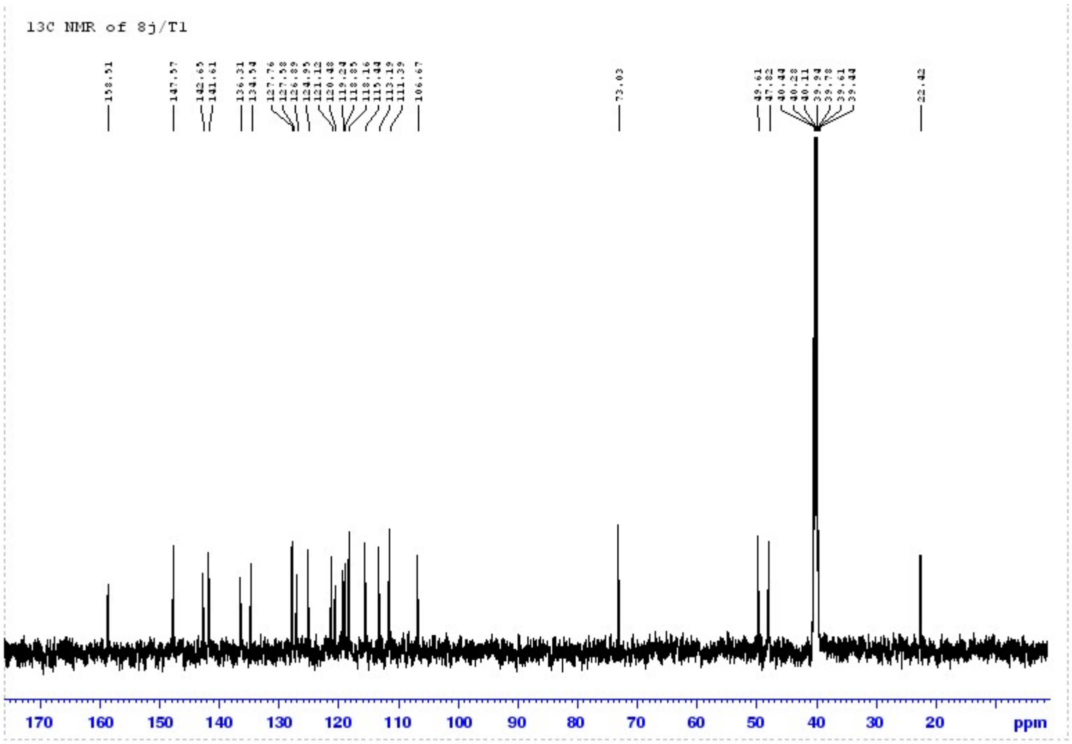
13C NMR of 8j/T1
158.51
147.57
142.65
141.61
136.31
134.54
127.76
127.58
126.89
124.95
121.12
120.48
119.24
118.85
118.16
115.44
113.19
111.39
106.67
73.03
49.61
47.82
40.44
40.28
40.11
39.94
39.78
39.61
39.44
22.42
170
160
150
140
130
120
110
100
90
80
70
60
50
40
30
20
ppm

HSQC of 8j/T1

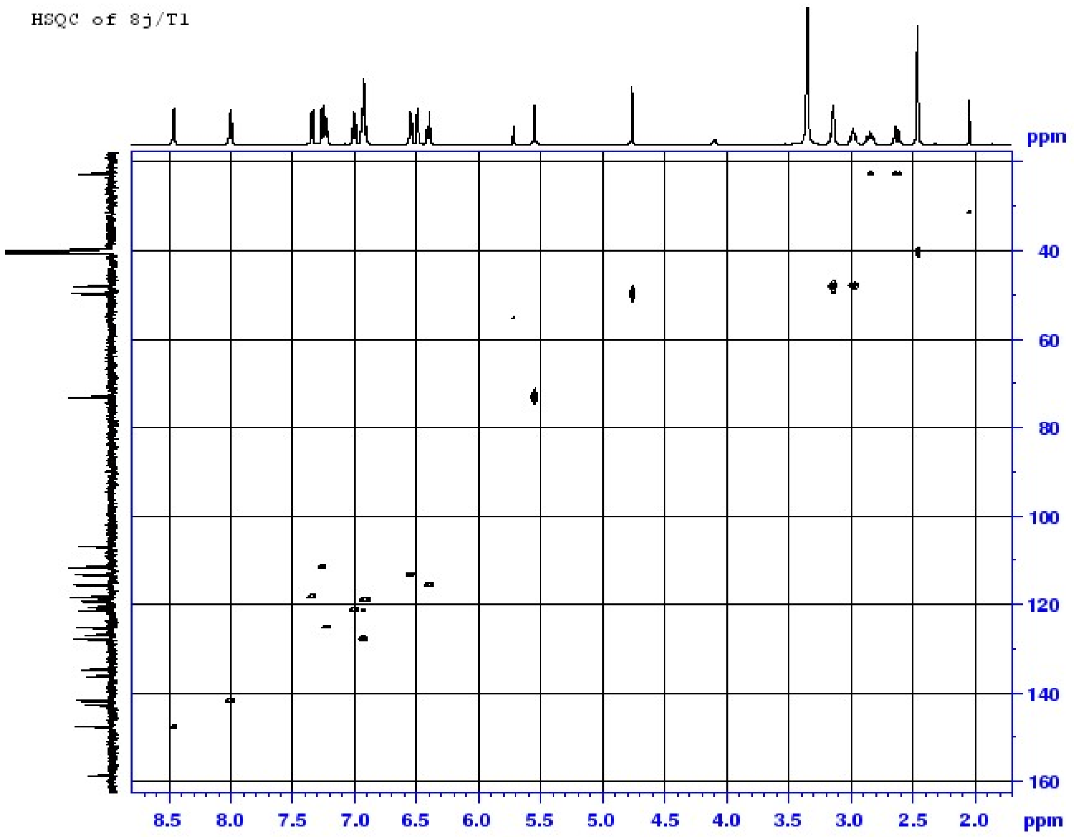

1H NMR of 8k/T1

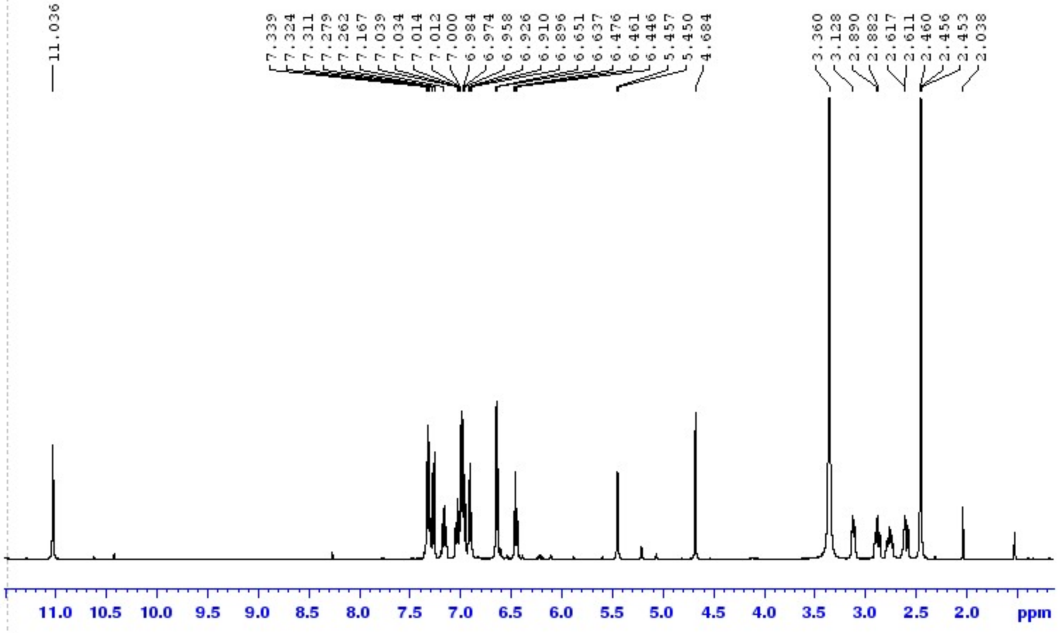

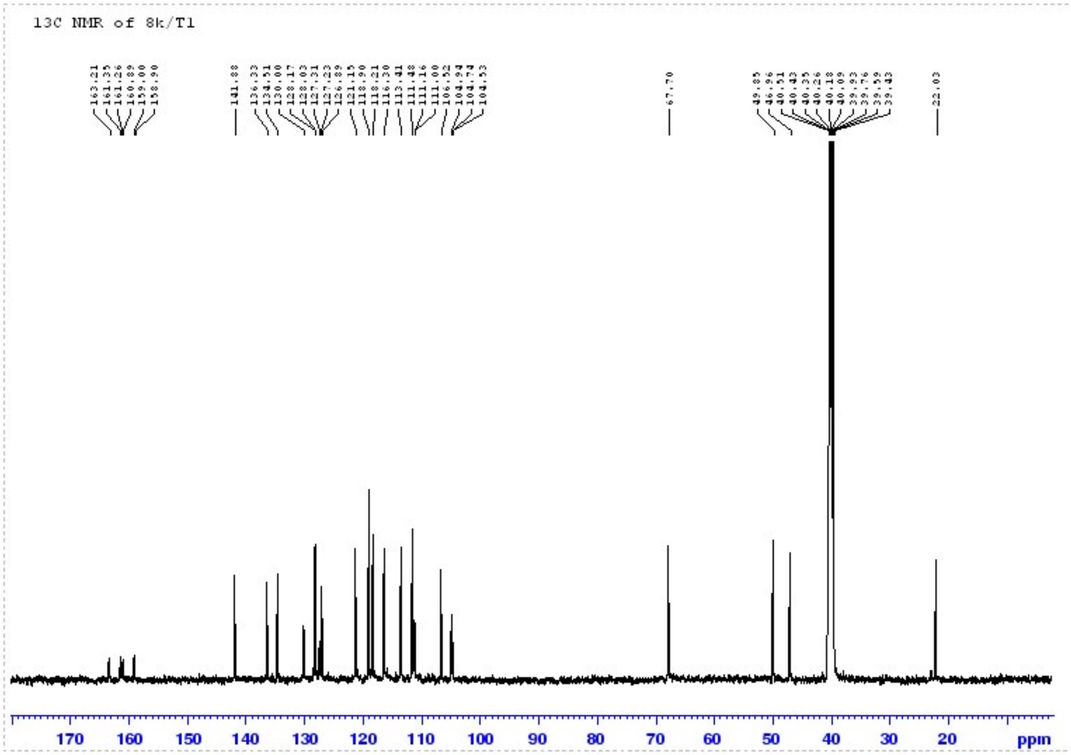
13C NMR of 8k/T1
ppm

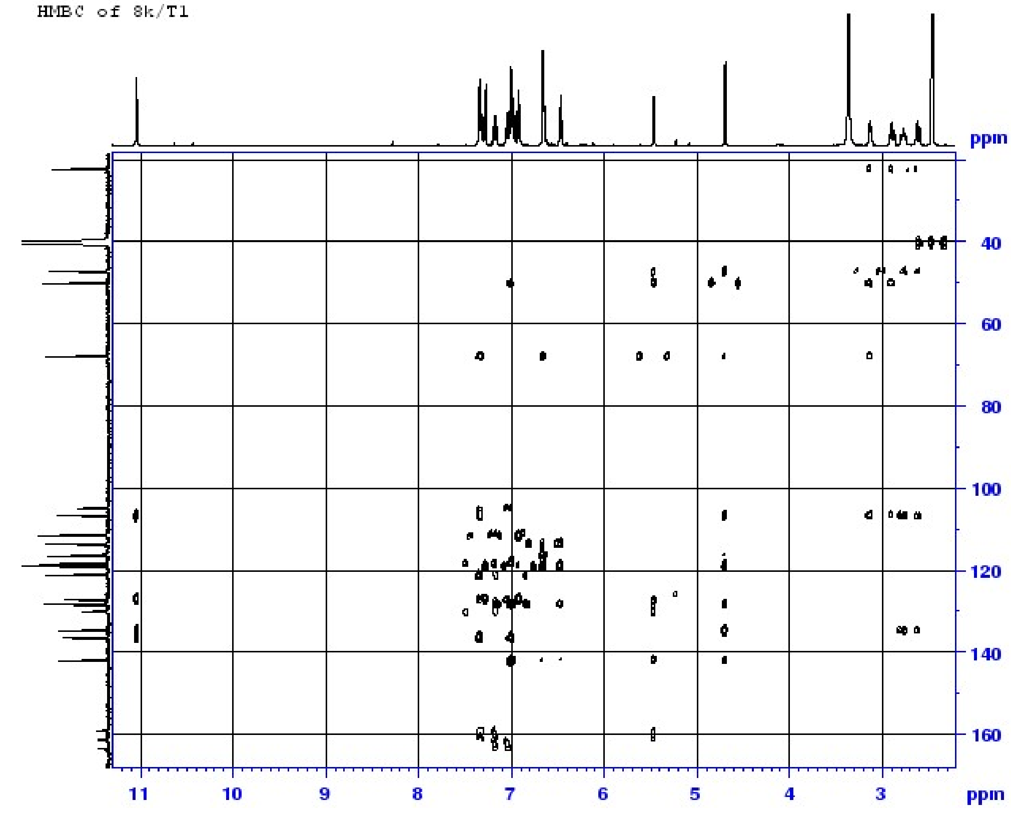
HMBC of 8k/T1
ppm

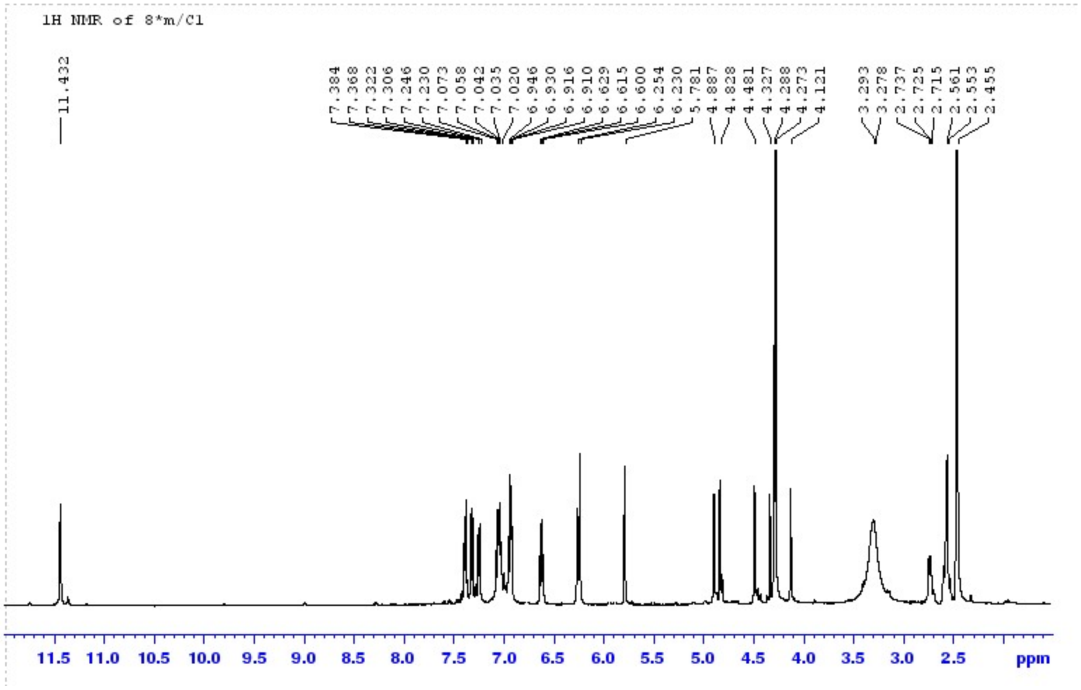
1H NMR of 8*m/Cl
ppm

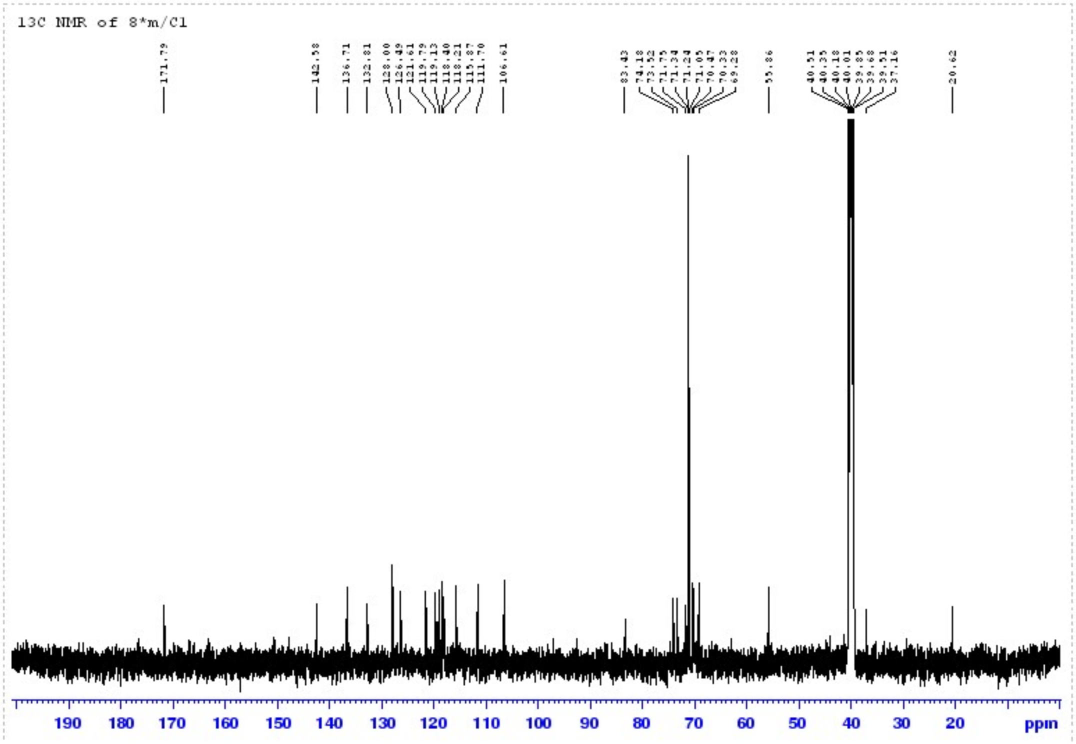
13C NMR of 8*m/Cl
ppm

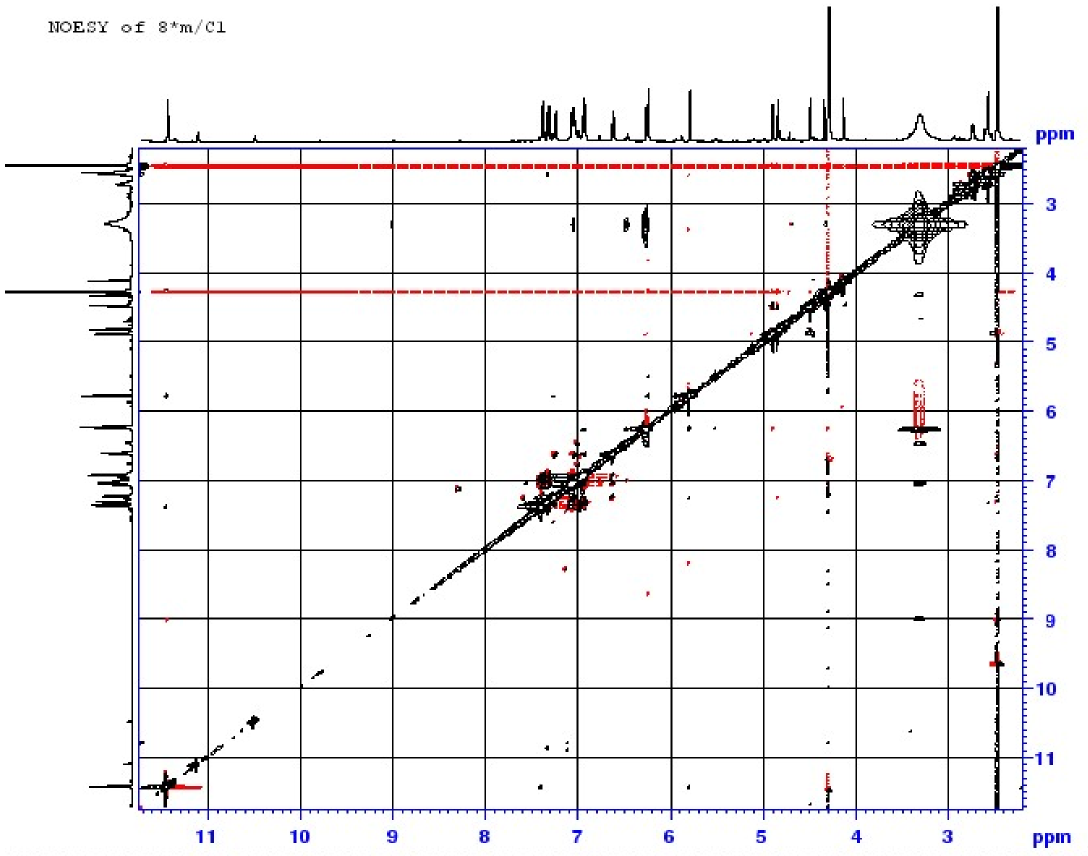
NOESY of 8*m/Cl
ppm
3
4
5
6
7
8
9
10
11
11
10
9
8
7
6
5
4
3
ppm

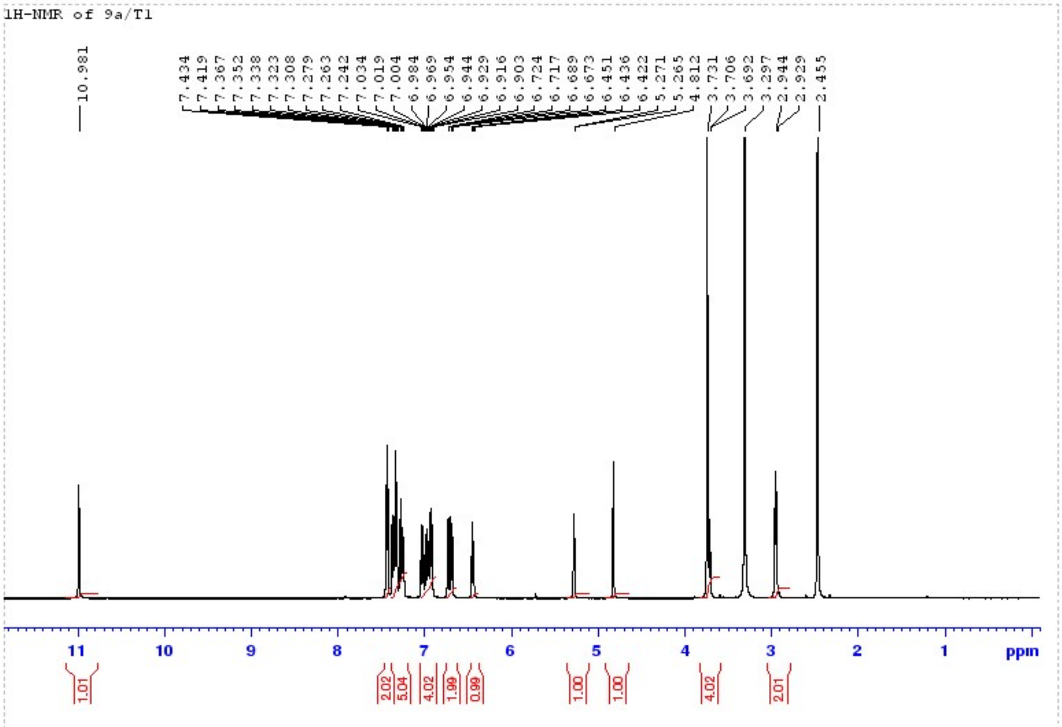
1H-NMR of 9a/T1
10.981
7.434
7.419
7.367
7.352
7.338
7.323
7.308
7.279
7.263
7.242
7.034
7.019
7.004
6.984
6.969
6.954
6.944
6.929
6.916
6.903
6.724
6.717
6.689
6.673
6.451
6.436
6.422
5.271
5.265
4.812
3.731
3.706
3.692
3.297
2.944
2.929
2.455
11
10
9
8
7
6
5
4
3
2
1
ppm
1.01
2.02
5.04
4.02
1.99
0.99
1.00
1.00
4.02
2.01

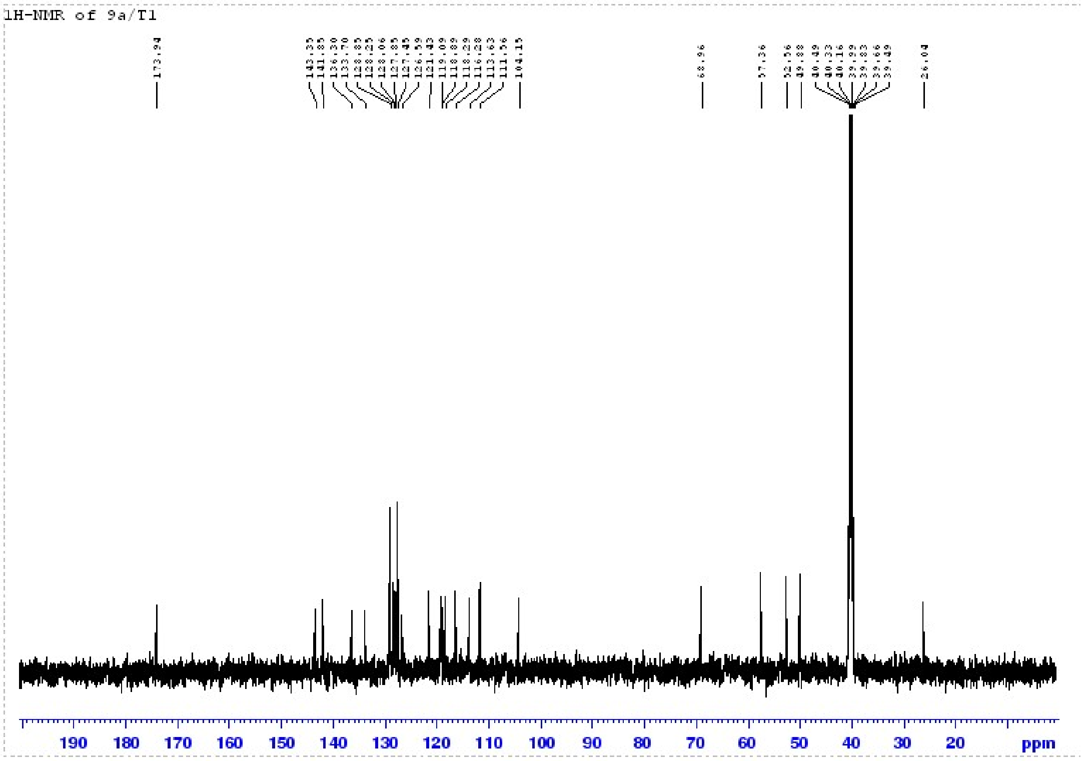
1H-NMR of 9a/T1
173.94
143.35
141.85
136.30
133.70
128.85
128.25
128.06
127.85
127.45
126.59
121.43
119.09
118.89
118.29
116.28
113.63
111.56
104.15
68.96
57.36
52.56
49.88
40.49
40.33
40.16
39.99
39.83
39.66
39.49
26.04
190
180
170
160
150
140
130
120
110
100
90
80
70
60
50
40
30
20
ppm

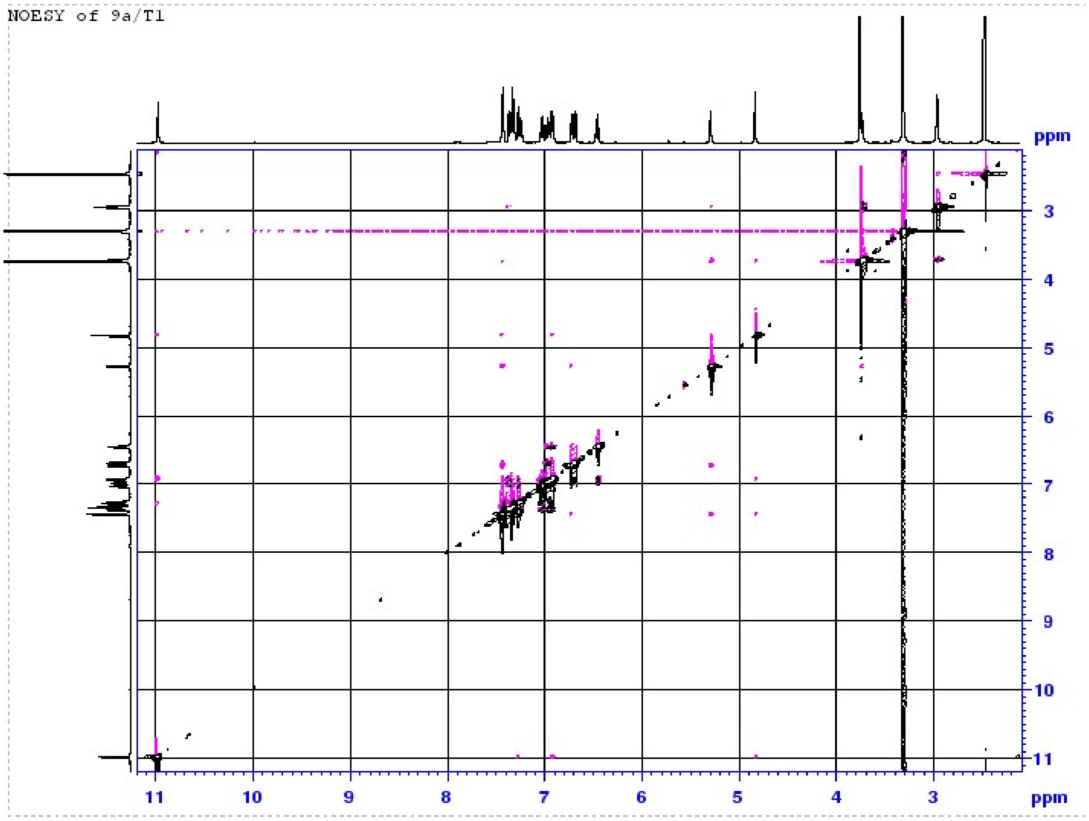
NOESY of 9a/T1
ppm
3
4
5
6
7
8
9
10
11
11
10
9
8
7
6
5
4
3
ppm

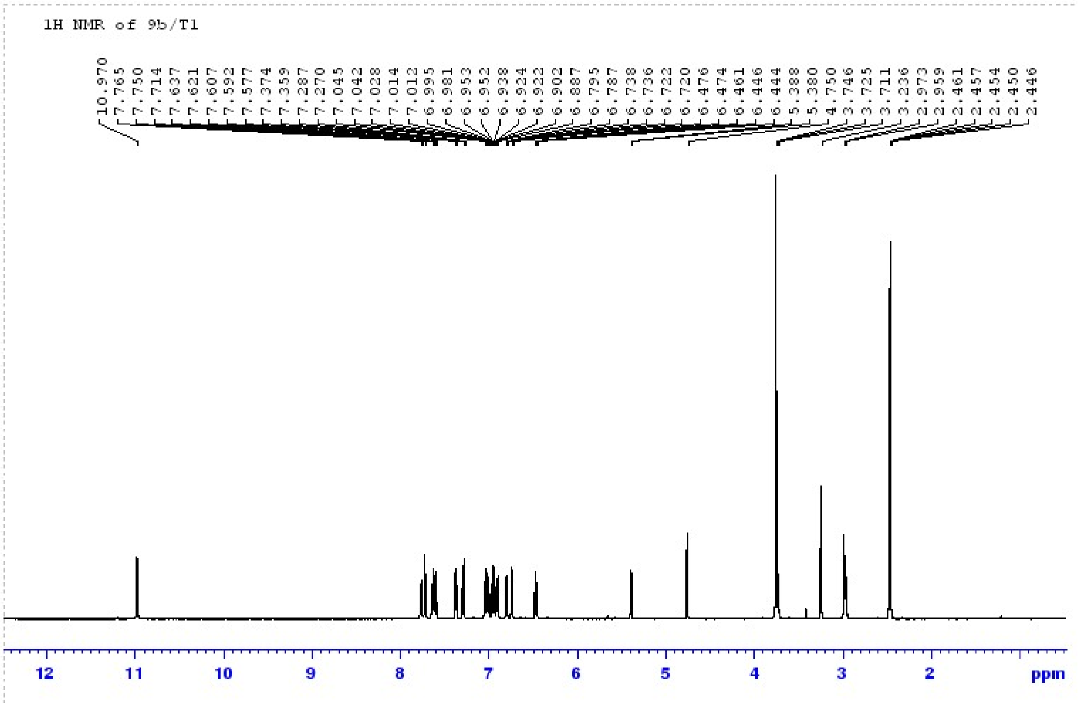
1H NMR of 9b/T1
10.970
7.765
7.750
7.714
7.637
7.621
7.607
7.592
7.577
7.374
7.359
7.287
7.270
7.045
7.042
7.028
7.014
7.012
6.995
6.981
6.953
6.952
6.938
6.924
6.922
6.902
6.887
6.795
6.787
6.738
6.736
6.722
6.720
6.476
6.474
6.461
6.446
6.444
5.388
5.380
4.750
3.746
3.725
3.711
3.236
2.973
2.959
2.461
2.457
2.454
2.450
2.446
12
11
10
9
8
7
6
5
4
3
2
ppm

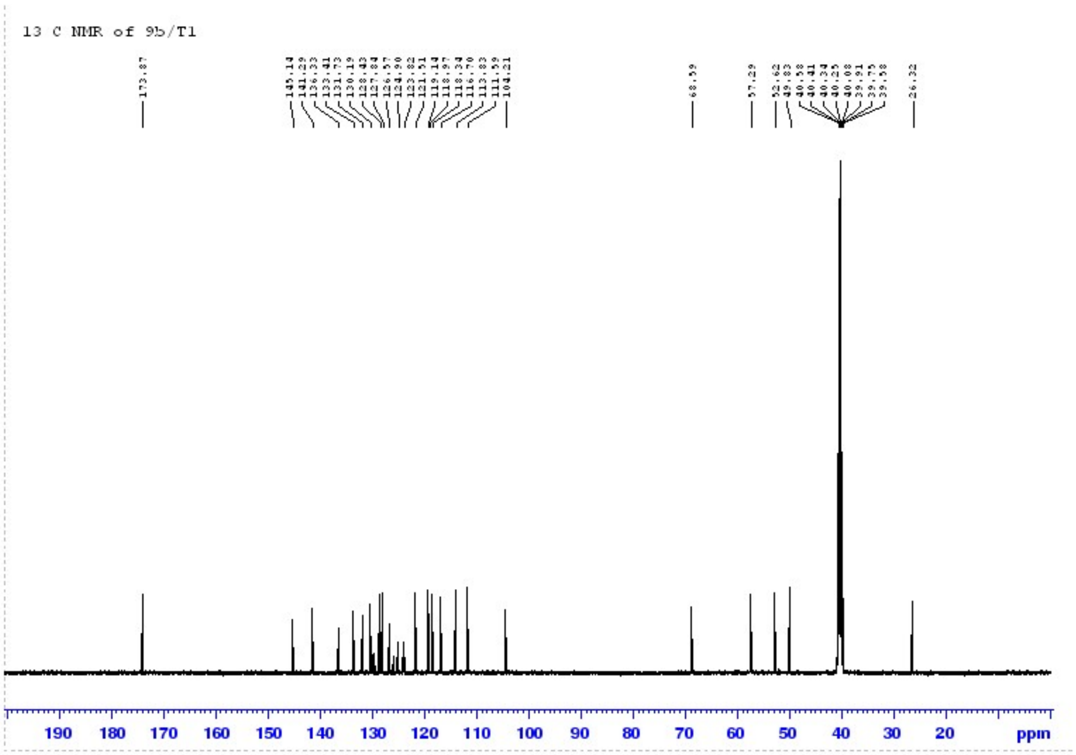
13 C NMR of 9b/T1
173.87
145.14
141.29
136.33
133.41
131.73
130.19
128.43
127.84
126.57
124.90
123.82
121.51
119.14
118.97
118.34
116.70
113.83
111.59
104.21
68.59
57.29
52.62
49.83
40.58
40.41
40.34
40.25
40.08
39.91
39.75
39.58
26.32
190
180
170
160
150
140
130
120
110
100
90
80
70
60
50
40
30
20
ppm

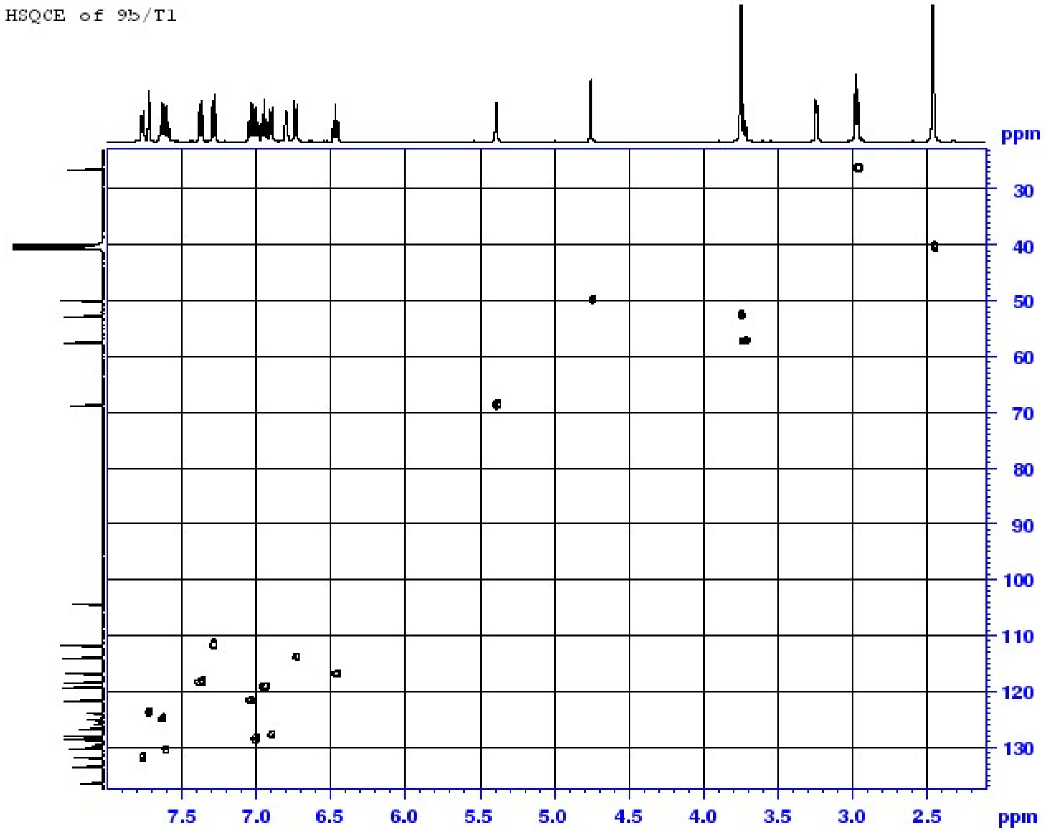
HSQCE of 9b/T1
ppm
30
40
50
60
70
80
90
100
110
120
130
7.5
7.0
6.5
6.0
5.5
5.0
4.5
4.0
3.5
3.0
2.5
ppm

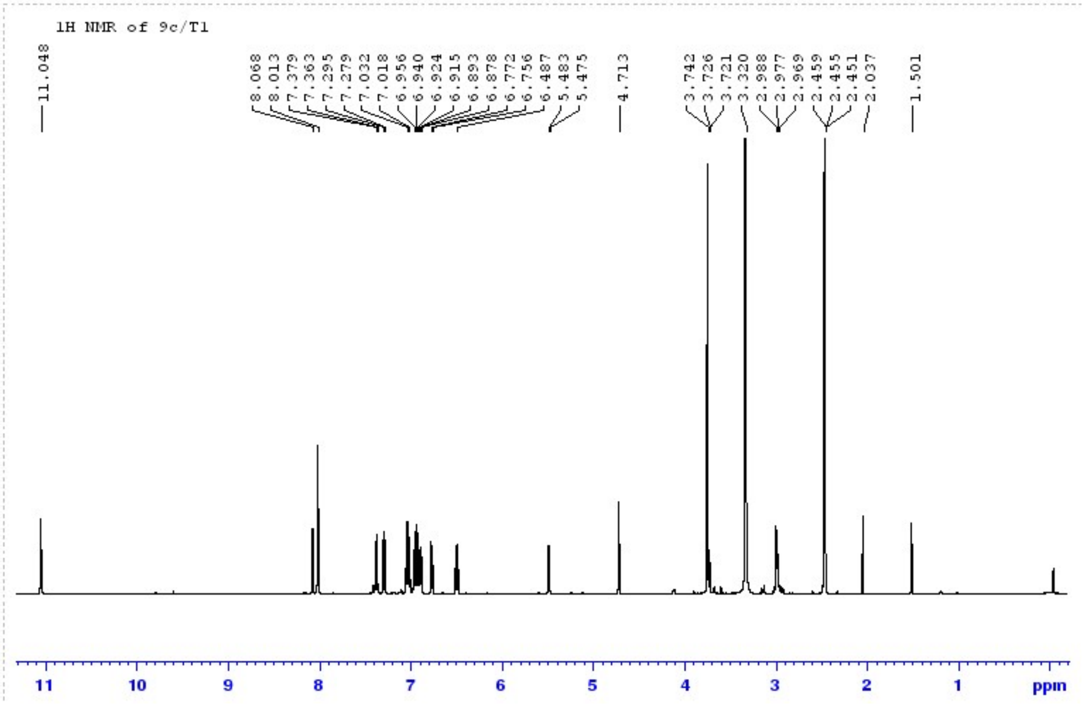
1H NMR of 9c/T1
11.048
8.068
8.013
7.379
7.363
7.295
7.279
7.032
7.018
6.956
6.940
6.924
6.915
6.893
6.878
6.772
6.756
6.487
5.483
5.475
4.713
3.742
3.726
3.721
3.320
2.988
2.977
2.969
2.459
2.455
2.451
2.037
1.501
11
10
9
8
7
6
5
4
3
2
1
ppm

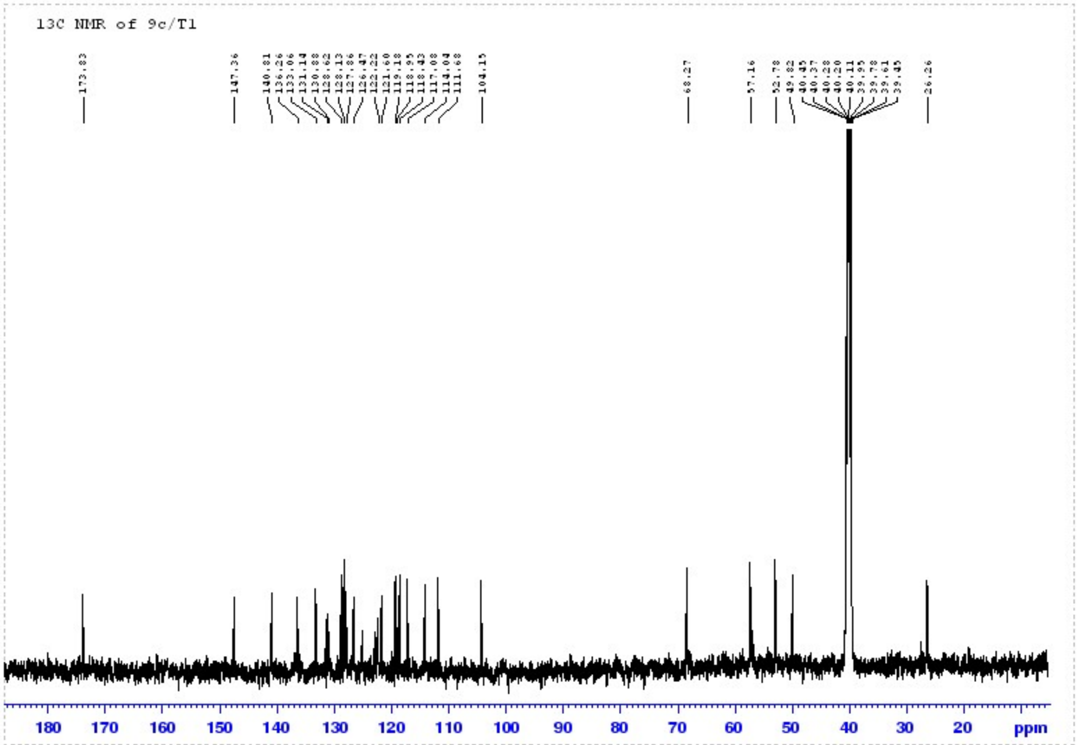
13C NMR of 9c/T1
173.83
147.36
140.81
136.26
133.06
131.14
130.88
128.62
128.13
127.86
126.47
122.22
121.60
119.18
118.95
118.43
117.08
114.04
111.68
104.15
68.27
57.16
52.78
49.82
40.45
40.37
40.28
40.20
40.11
39.95
39.78
39.61
39.45
26.26
180
170
160
150
140
130
120
110
100
90
80
70
60
50
40
30
20
ppm

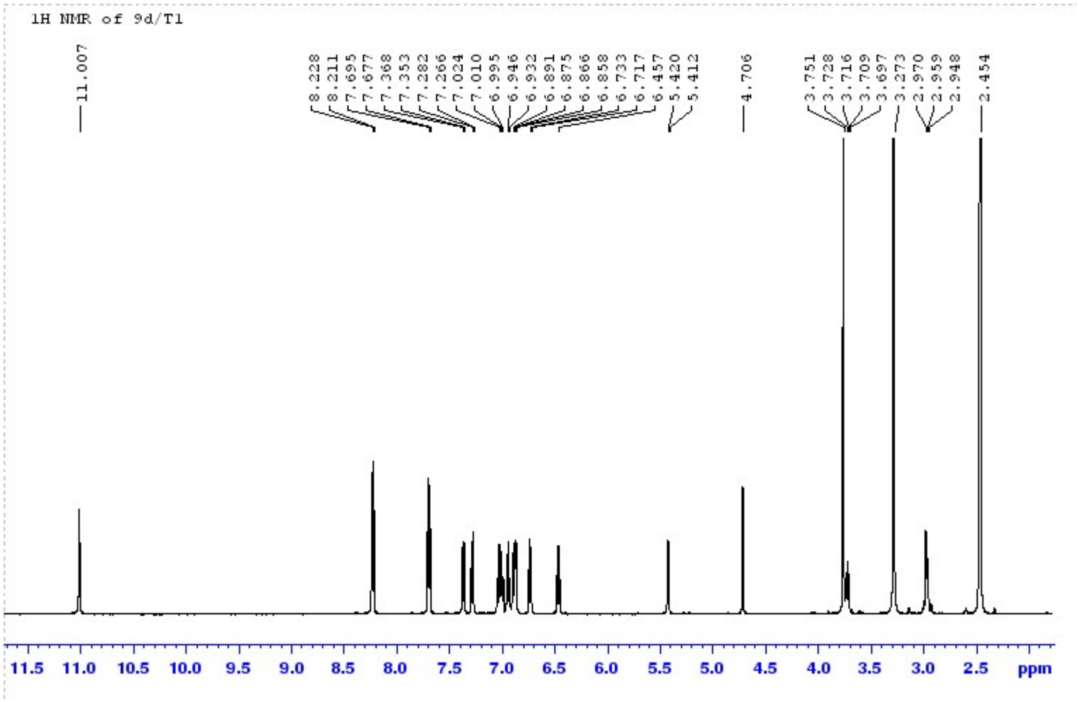
1H NMR of 9d/T1
ppm

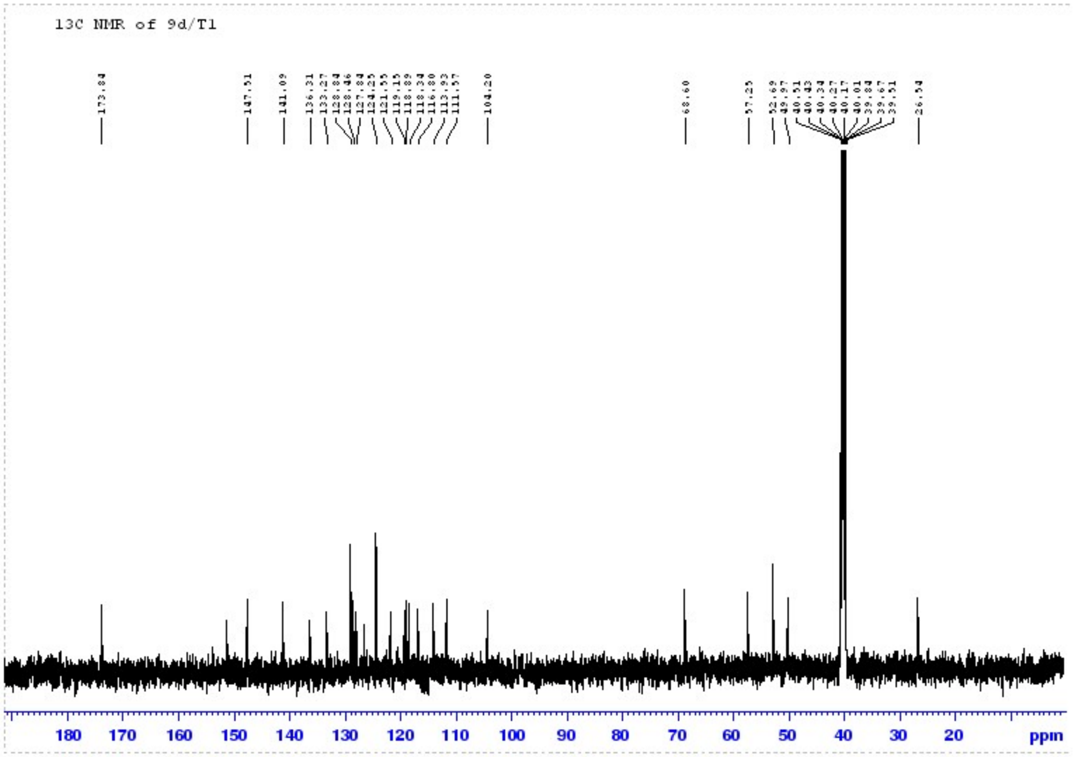
13C NMR of 9d/T1
ppm

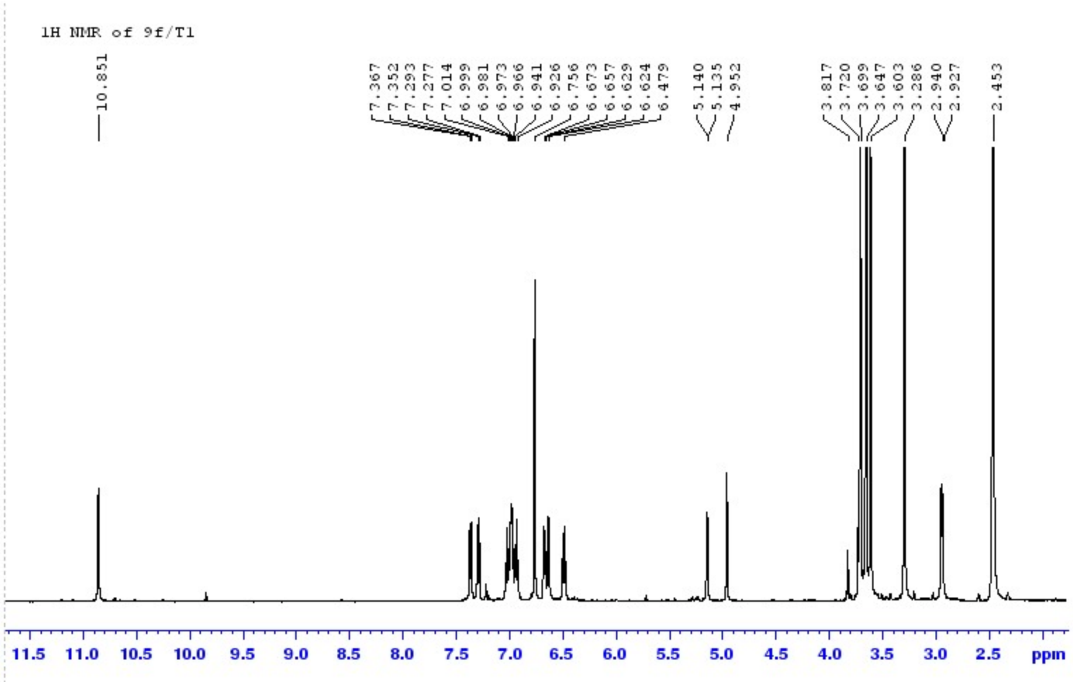
1H NMR of 9f/T1
ppm

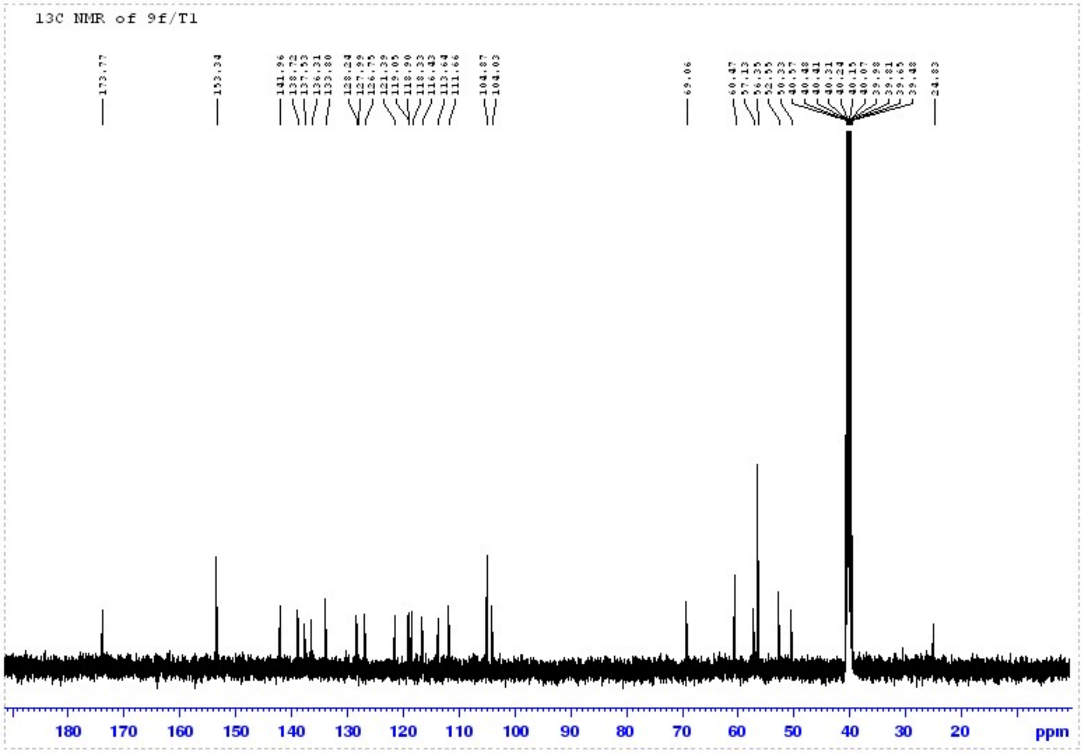
13C NMR of 9f/T1
ppm

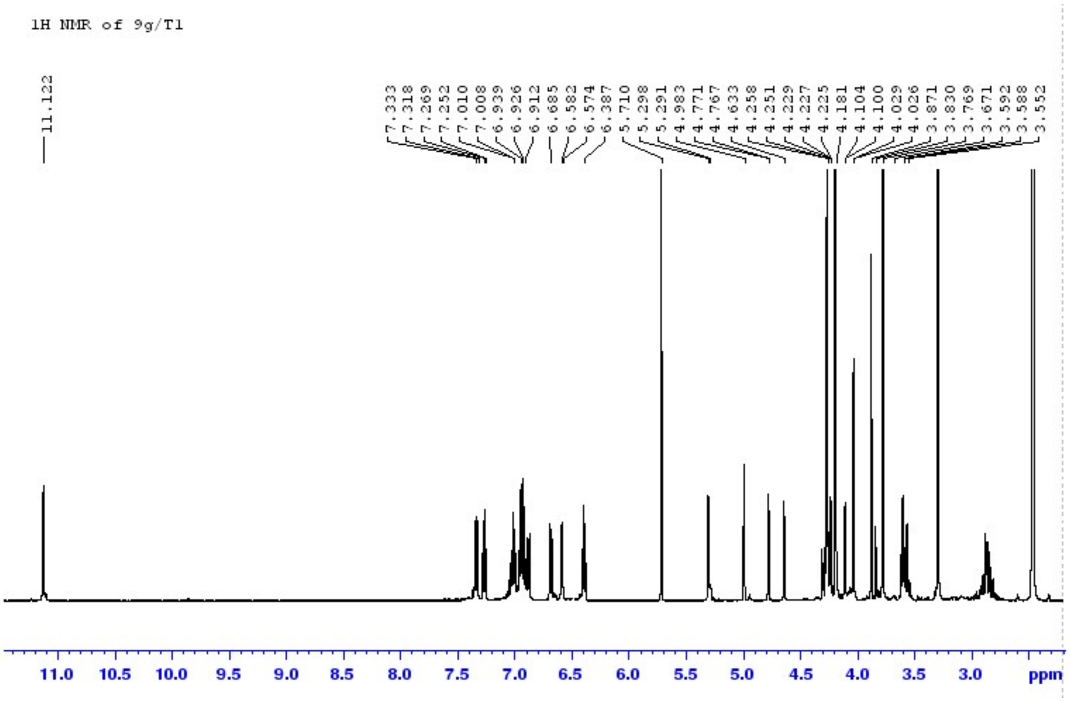
1H NMR of 9g/T1
11.122
7.333 7.318 7.269 7.252 7.010 7.008 6.939 6.926 6.912 6.685 6.582 6.574 6.387 5.710 5.298 5.291 4.983 4.771 4.767 4.633 4.258 4.251 4.229 4.227 4.225 4.181 4.104 4.100 4.029 4.026 3.871 3.830 3.769 3.671 3.592 3.588 3.552
11.0 10.5 10.0 9.5 9.0 8.5 8.0 7.5 7.0 6.5 6.0 5.5 5.0 4.5 4.0 3.5 3.0 ppm

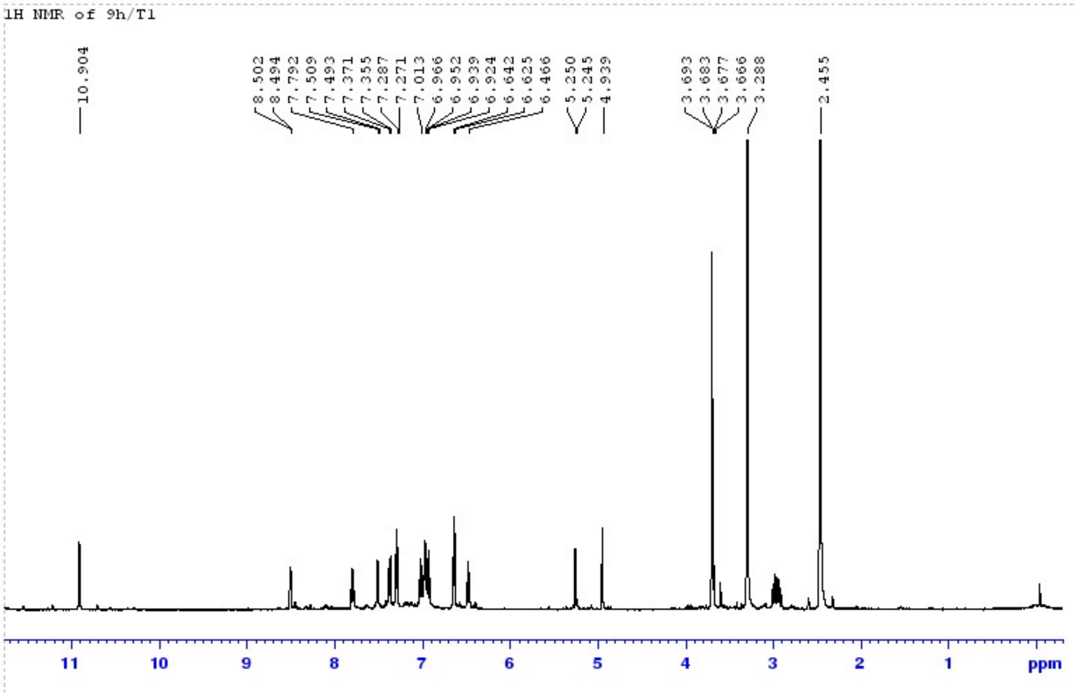
1H NMR of 9h/T1
10.904
8.502 8.494 7.792 7.509 7.493 7.371 7.355 7.287 7.271 7.013 6.966 6.952 6.939 6.924 6.642 6.625 6.466
5.250 5.245 4.939
3.693 3.683 3.677 3.666 3.288
2.455
11 10 9 8 7 6 5 4 3 2 1 ppm

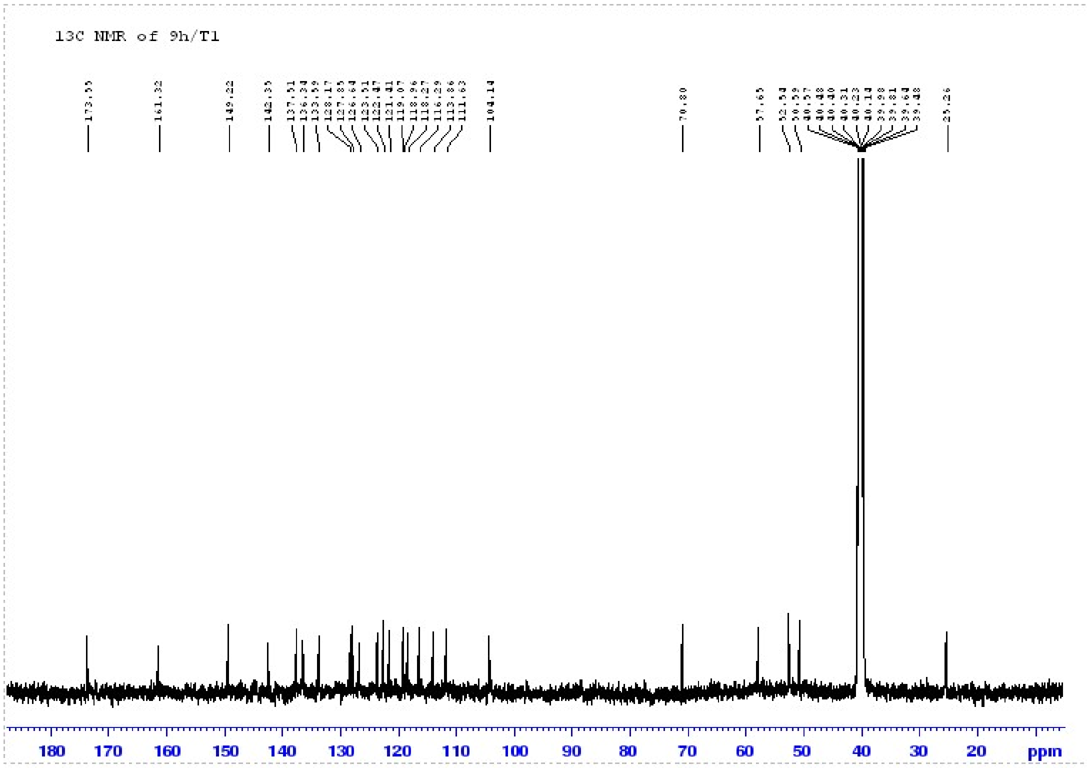
13C NMR of 9h/T1
ppm

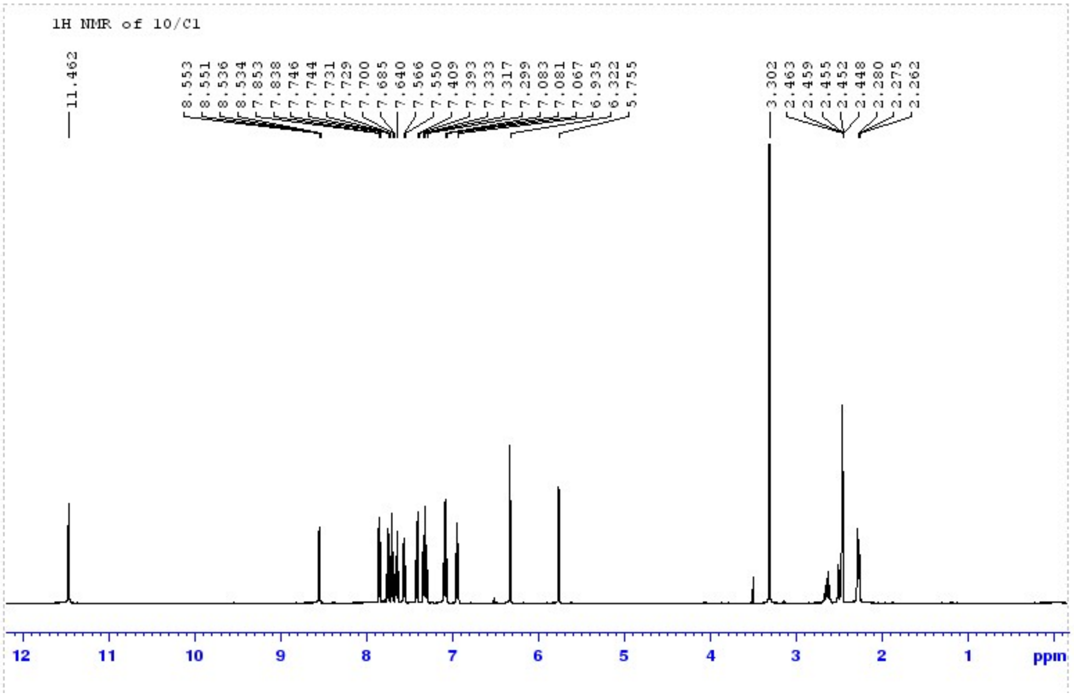
1H NMR of 10/C1
ppm

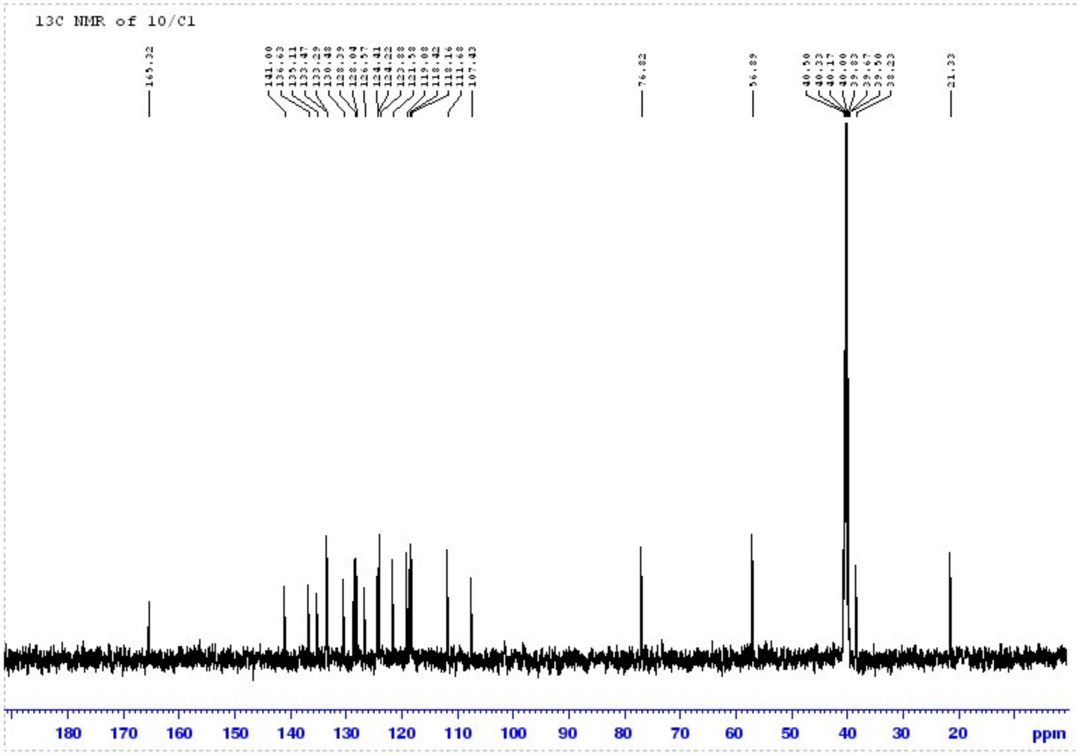
13C NMR of 10/C1
165.32
141.00
136.63
135.11
133.47
133.29
130.48
128.39
128.04
126.57
124.41
124.22
123.88
121.58
119.08
118.42
118.16
111.68
107.43
76.82
56.89
40.50
40.33
40.17
40.00
39.83
39.67
39.50
38.23
21.33
180 170 160 150 140 130 120 110 100 90 80 70 60 50 40 30 20 ppm

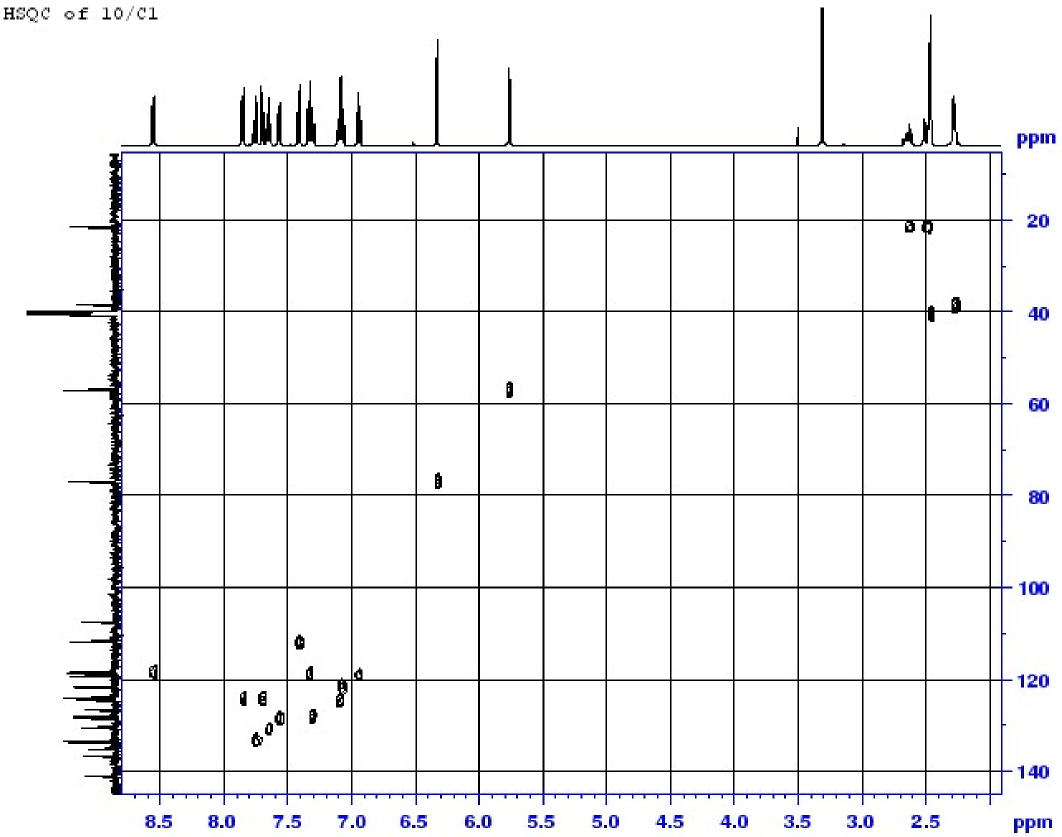
HSQC of 10/C1
ppm
20
40
60
80
100
120
140
8.5 8.0 7.5 7.0 6.5 6.0 5.5 5.0 4.5 4.0 3.5 3.0 2.5
ppm

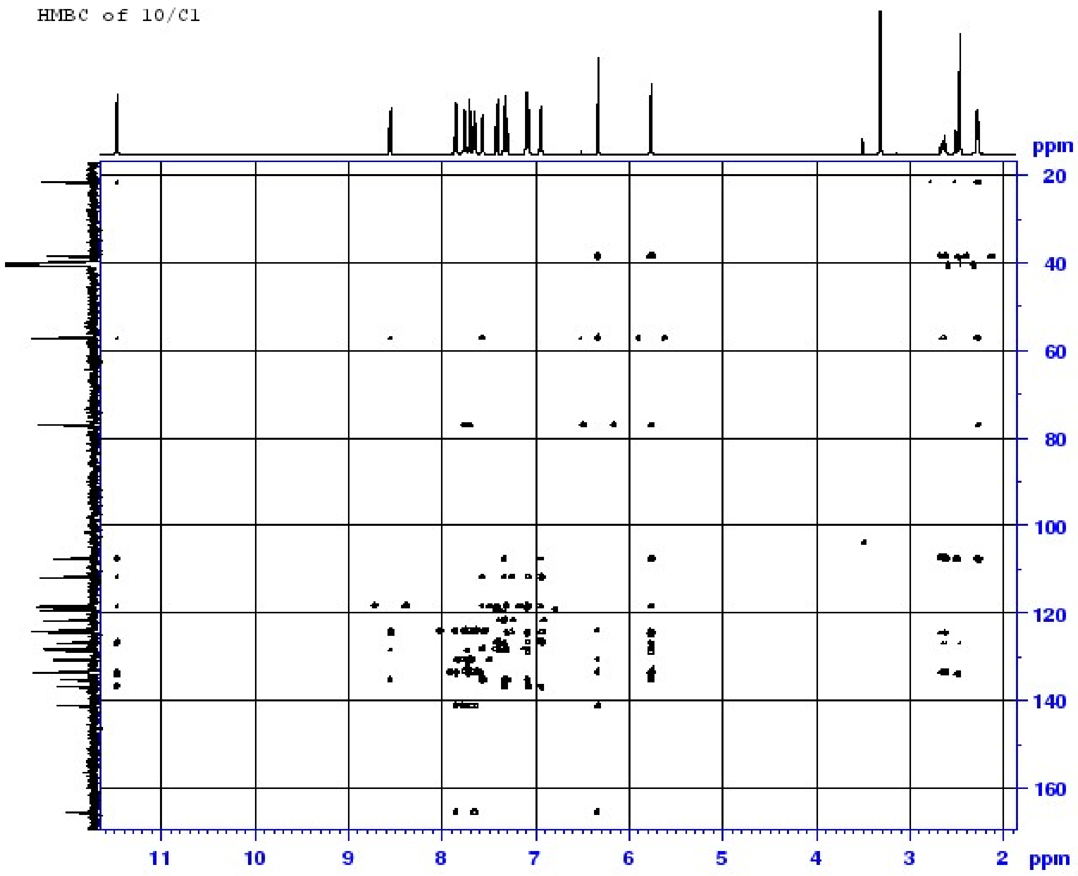
HMBC of 10/Cl
ppm
20
40
60
80
100
120
140
160
11
10
9
8
7
6
5
4
3
2
ppm

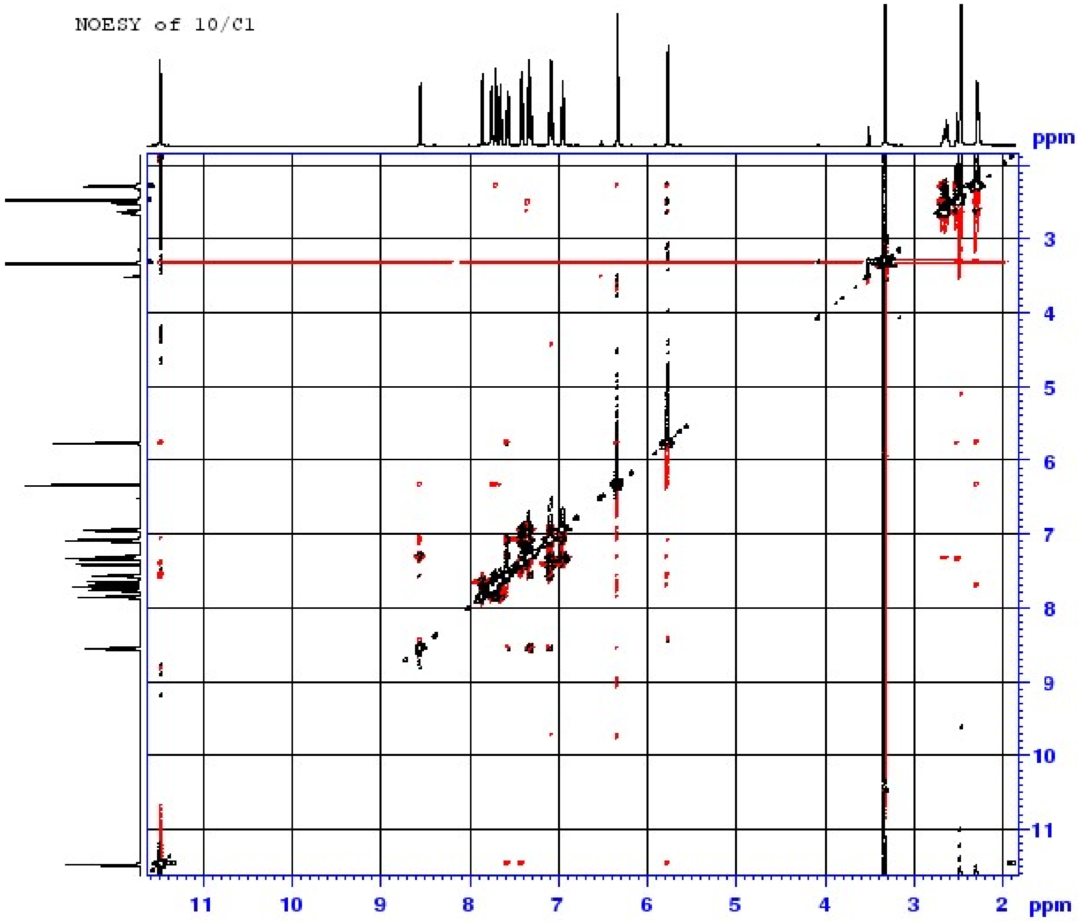
NOESY of 10/C1
ppm
ppm

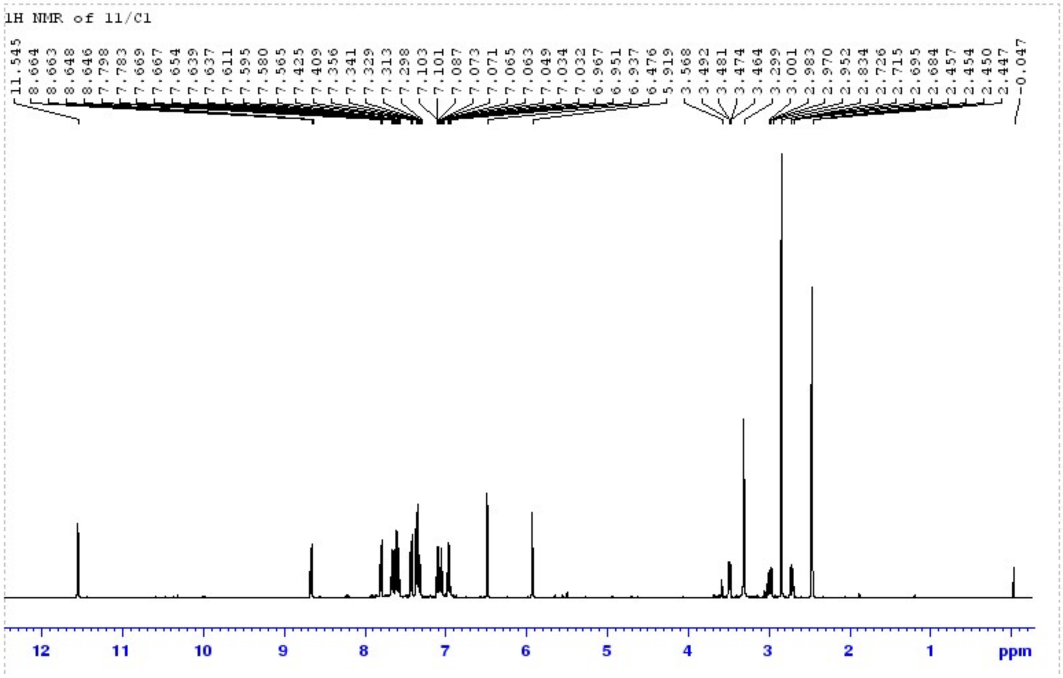
1H NMR of 11/Cl
ppm

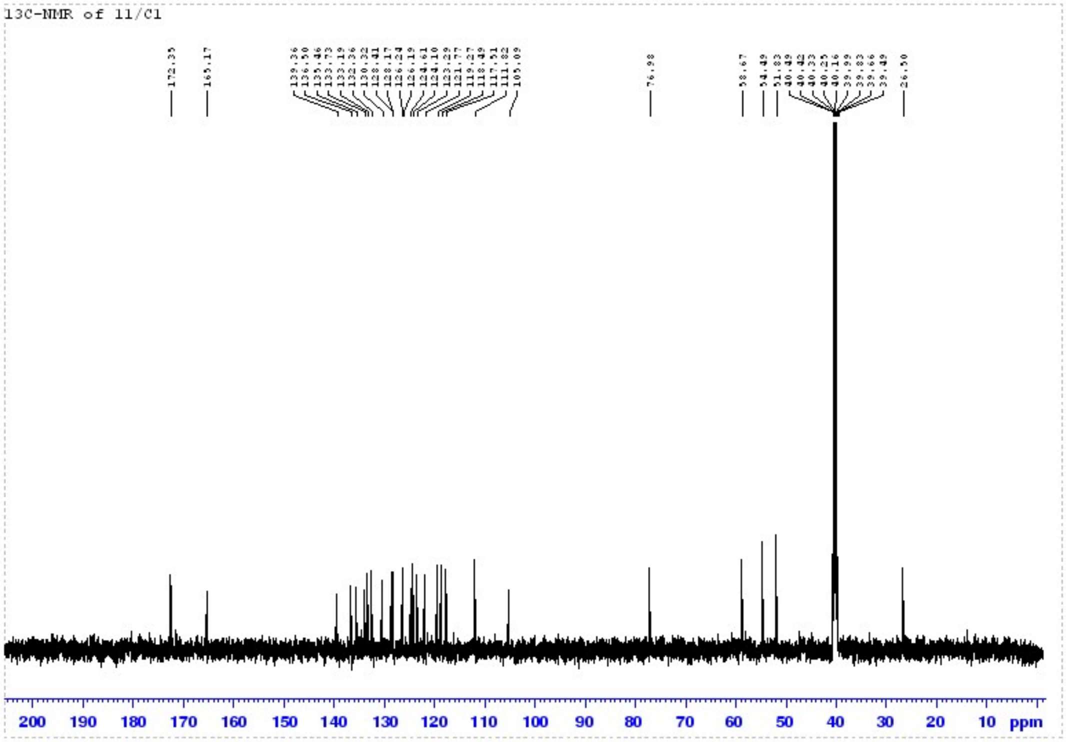
13C-NMR of 11/Cl
ppm

HSQC of 11/C1

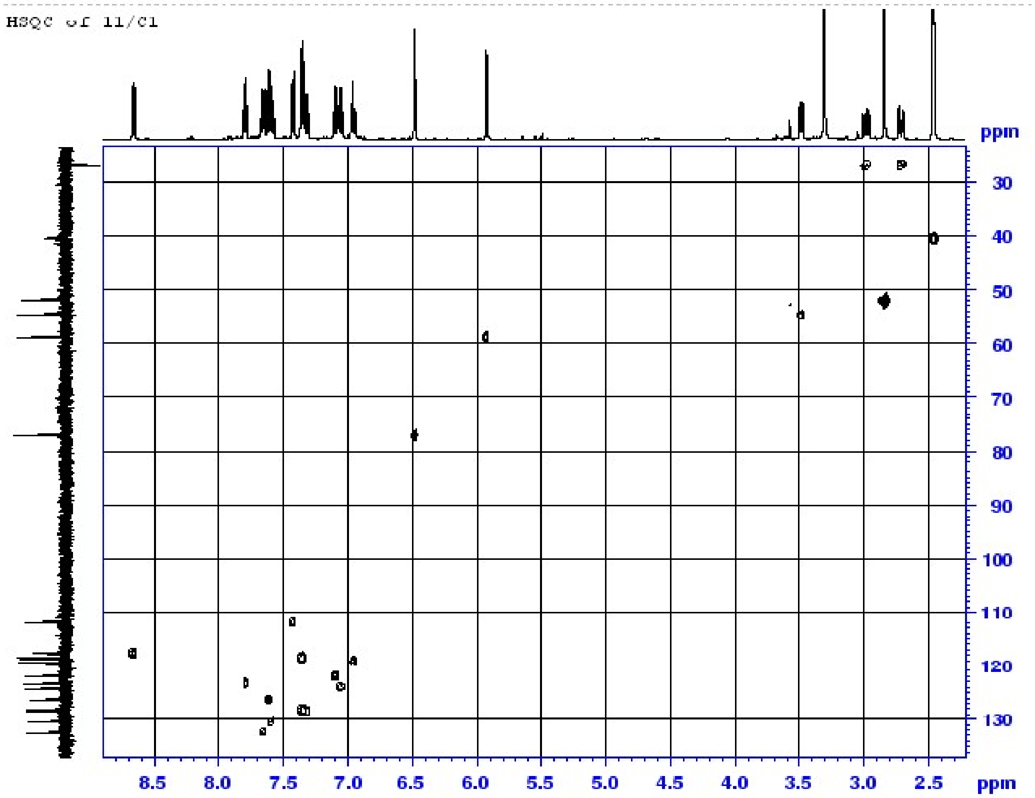

HMBC of 11/C1

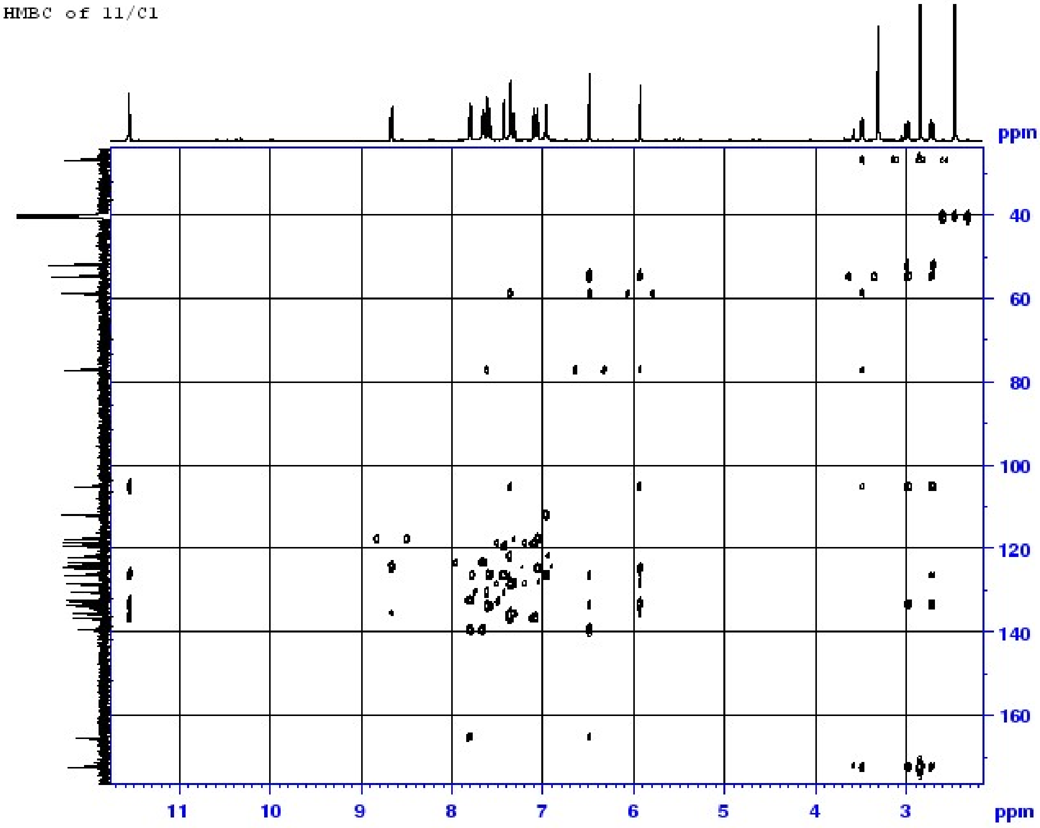

COSY (aromatic region) of 11/C1

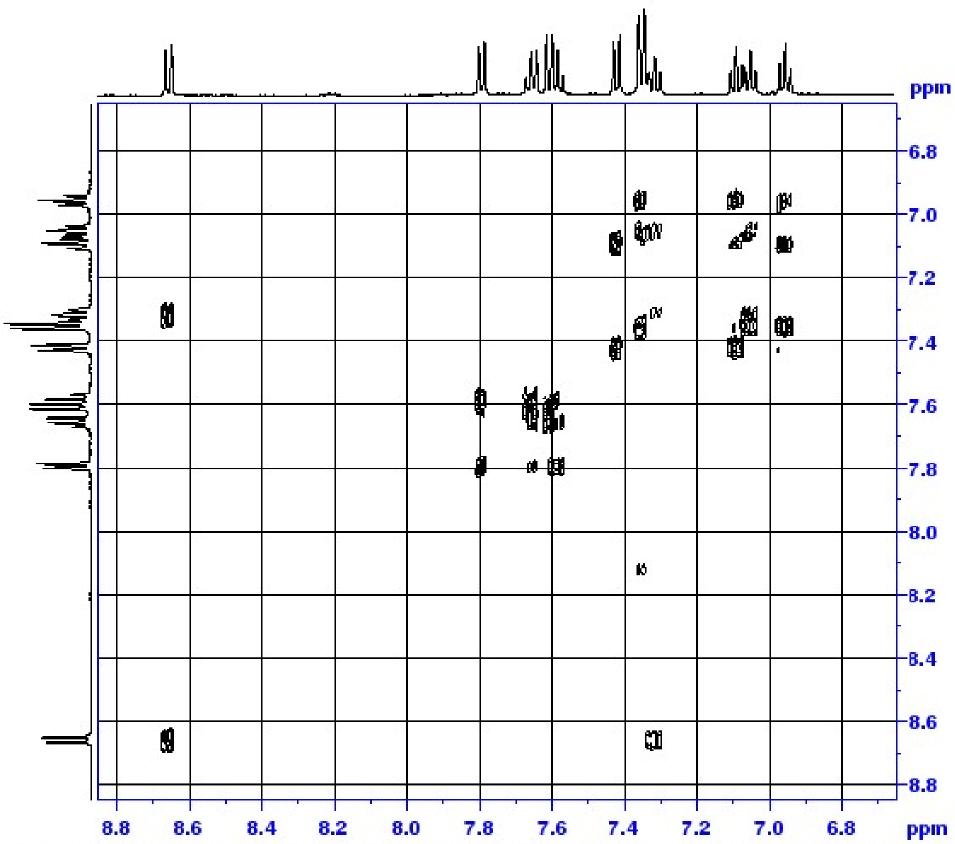

NOESY of 11/C1

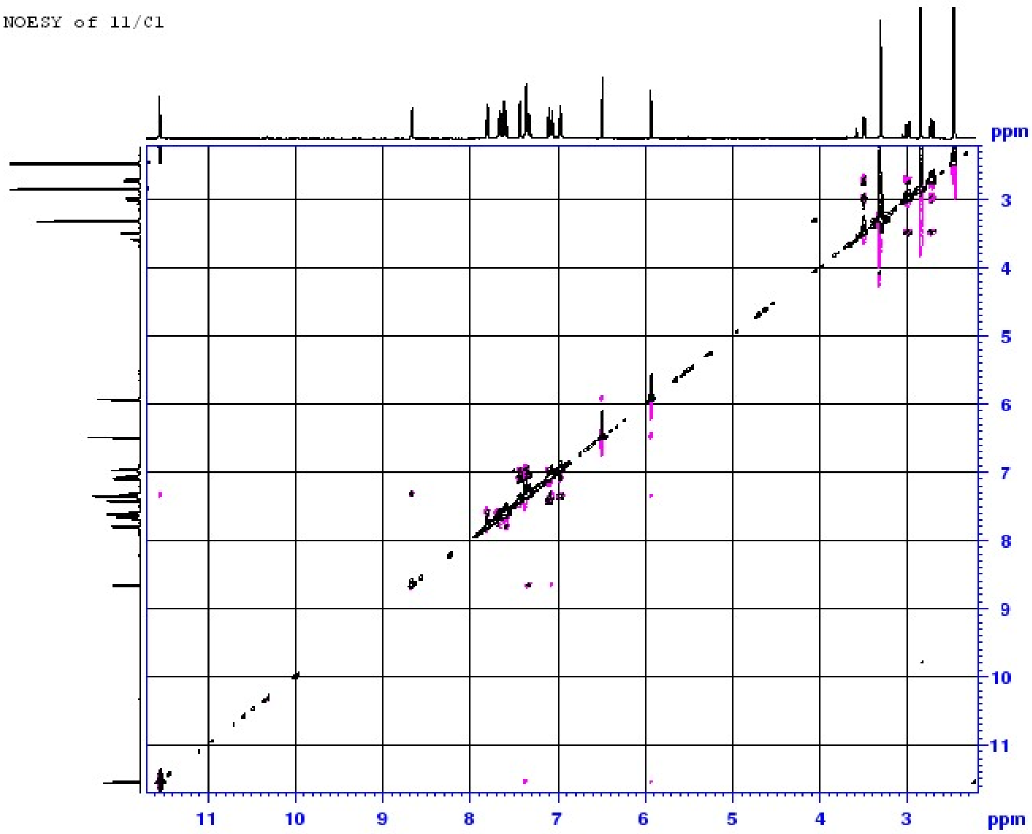

S.4. Crystallographic study on Methyl (6S,8SS,14bR)6-(3,4,5-trimethoxyphenyl)-5,6,8,9,14b-hexahydroindolo[2′,3′:3,4]pyrido[1,2- c]quinazoline-8-carboxylate (9f/T1)

***S.4.1.* Experimental**

Compound **9f/T1** was crystallized from dimethyl sulfoxide. X-ray diffraction data were collected on a Rigaku XtaLab Synergy-R diffractometer using Cu -Kα radiation (λ= 1.54184 Å). Data reduction was carried out using the software provided with the diffractometer (CrysAlisPro 1.171.40.14e (Rigaku OD, 2018)). The structure was solved by direct methods using Olex2 v1.2 [1] and refined by full-matrix least-squares techniques (SHELXL-2014/7) on F^2 [2]. The position of the hydrogen bound to N5 was determined based on difference electron density, and it was refined unrestrained. All the other hydrogen atoms were refined in the riding positions. The crystallographic parameters, data collection, and structure refinement details are summarized in Table 1. Compound **9f/T1** crystallized in space group $P2_12_12_1$. Flack-x parameter refined to a value of 0.00(2). The asymmetric unit contains one molecule of **1** and two dimethyl sulfoxide molecules, both in disordered orientations. Sulphur atoms of the solvent molecules were refined in alternate positions, however corresponding alternate positions of the methyl groups could not be resolved. The occupancies of the two disordered solvent molecule pairs were refined independently, resulting significant deviation from 0.5. Anisotropic displacement parameters' similarity restrains were applied to the solvent molecules. Four reflections were omitted during refinement due to systematic error. The structures were analyzed and the figures created using Mercury program [3]. Validation was carried out using CheckCIF/PLATON [4]. The Structure of **9f/T1** was deposited with the Cambridge Crystallographic Data Centre and can be obtained free of charge with CCDC deposition numbers (CCDC number: 1990174).

Table 1 X-ray crystallograqphic data and structural refinements details for **9f/T1**

**Compound**	**9f/T1**
Asymmetric unit contents	$C_{33}H_{41}N_3O_7S_2$
Empirical formula	$C_{29}H_{29}N_3O_5$ 2(C_2H_6OS)
Formula weight	655.81
Temperature (K)	299.0 (2)
Wavelength (Å)	1.54184
Crystal system	orthorhombic
Space group	$P2_12_12_1$
Unit cell dimensions *a*, *b*, *c* (Å)	8.6673(2), 21.3225(5), 17.9732(4)
α, β, γ (°)	90.0, 90.0, 90.0
V (Å^3)	3321.6(1)
Z	4
D_{calc} (g /cm^3)	1.311
θ_{min}, θ_{max} (°)	3.216, 75.525
Reflections collected/unique	29556 / 6600
R_{int}	0.0576
Completeness to θ_{max} (%)	0.972
Data/restraints/parameters	6600 / 84 / 433
Goodness-of-fit (GOF) on F^2	1.099
R1 / *wR2* [$I > 2\sigma(I)$]	0.0623 / 0.1608
R1 / *wR2* (all data)	0.0720 / 0.1673
Largest difference peak/hole (e/ Å^3)	0.388 / -0.310

S.4.2. **Results**

Configuration of chirality centers was confirmed by the crystal structure of **9f/T1** (shown in Figure 1 with atom numbering) as follows: C6: *S*, C8: *S* and C14b: *R*, respectively.

The conformations of the ***tetrahydro-pyrimidine ring*** is half-chair (with N7 as puckering atom) allowing C14a to be in quasi-equatorial position (that is the junction to the most rigid part of the ring system), resulting its other two substituents taking axial positions (C8 within the beta-carboline-ring system, and C1′ of the bulky trimetoxphenyl group). The hydrogen atom position of the amin N5 was refined unrestrained and is equatorial forming a hydrogen bond. The ***tetrahydropyrimidine ring*** of the β-carboline moiety is also in a half chair conformation (with N7 as puckering atom) with two of its three substituents being in quasi-equatorial positions: C6 atom and the methyl-carboxylate group (C15). Selected geometrical parameters of the structure are listed in Table 2).

The overall shape of the molecule is distorted tetrahedral: the condensed ring system is bent with the trimethoxyphenyl moiety sticking out of its convex side carboxymethyl group protrudes from the concave side (The angles of the average plane of the ring system with planes of the trimethoxyphenyl and carboxymethyl groups are 86.7° and 70.1°, respectively.). This overall molecule shape might be the reason for the jigsaw puzzle - like crystal packing: no parallel alignment of the neighbor ring planes or formation of polar and apolar layers can be found. Instead, DMSO molecules fill the zigzag series of holes between molecules along unit cell axis *a* (Figure 2a): Both N-H hydrogen bond donors of the structure form hydrogen bond with a DMSO molecule.

Both DMSO solvate molecules are in disordered state, they are rotated by 180° degrees. This type of disorder is common in crystals with DMSO solvate [5], about half of these structures deposited in the Cambridge Structural database [6] are disordered. Interestingly the occupancies of the alternate orientations of the DMSO molecules significantly deviate from each other. Database analysis in the CSD showed that N...O=S angle of about 130° is characteristic to the most favorable orientation. In the present structure the occupancies of DMSO are governed by the strength of the hydrogen bond (Table 2): One of the DMSO orientations is close to the optimal geometry (N14-H14..O22=S21A), shifting the occupancy towards the better geometry (occupancies of S21A / S21B are 0.82 / 0.18, respectively). For the other hydrogen-bonding site, the occupancy difference of the alternate DMSO orientations is small, as for this weaker hydrogen bond their geometries are more similar to each other.

Weak C-H..O hydrogen bonds are also present in the crystal lattice. Though the molecule contains three aromatic rings, no π..π stacking interactions exist in the crystal, C-H.. π interactions are formed with the methyl groups and an edge-to-plane type weak interaction with C3-H group (Table 2, Figure 2b).

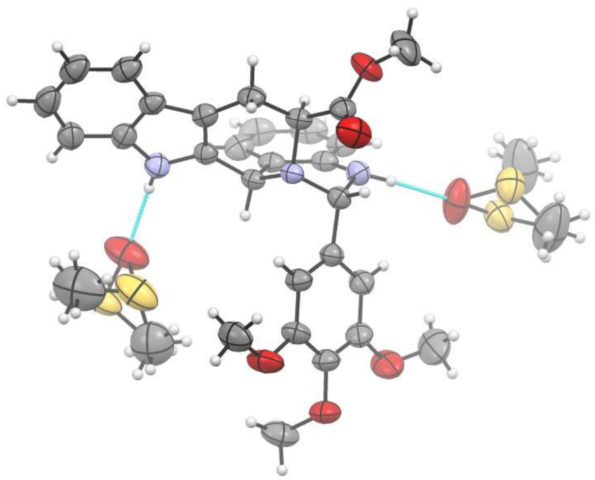

a) b)

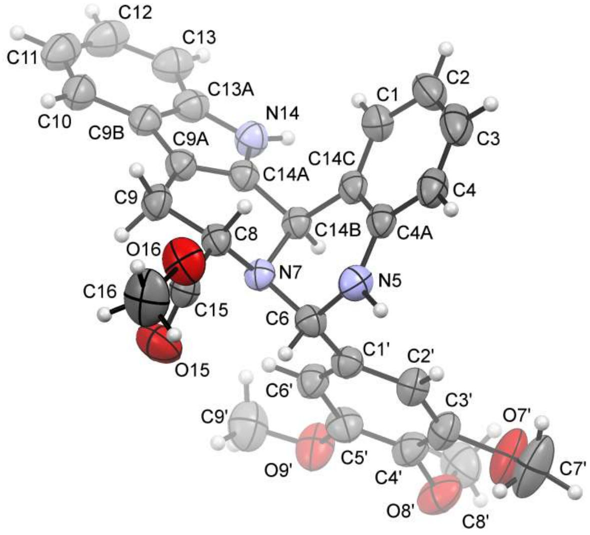

Figure 1. Crystal structure of compound **9f/T1**. a) The asymmetric unit contains one molecule **9f/T1** forming hydrogen bonds (shown in cyan) with two DMSO molecules, both with disordered sulphur positions. b) Close-up of molecule **9f/T1** with atomic labels and ring conformations shown. Atomic displacement ellipsoids are contoured at the 0.5 probability level.

a) 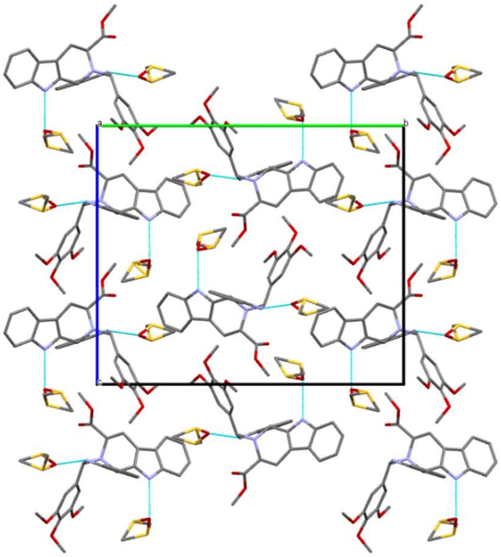

b)

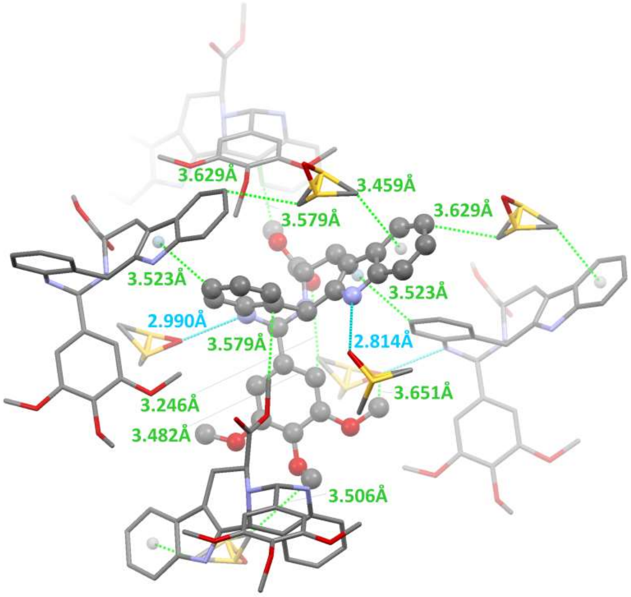

Figure 2. Crystal packing of **9f/T1**. a) View along unit cell axis *a*. Hydrogen atoms are not shown. b) One molecule of **9f/T1** forms direct hydrogen bonds (cyan) or C-H..O, C-H..π type weak interactions (green) with four neighboring molecules of **9f/T1** and six solvent molecules. (Hydrogen atoms are not shown.)

Table 2 Selected geometric parameters of **1**. (Variances in the last digits are shown in parentheses.)

**Compound**	**9f/T1**		
Bond lengths (Å)		*Bond angles(°) cont.*	
N5-C4A	1.377(6)	N7-C14B-C14A	107.4(3)
N5-C6	1.456(5)	N7-C14B-C14C	112.5(3)
N7-C6	1.471(5)	N5-C6-C1'	115.0(4)
N7-C8	1.487(5)	N7-C6-C1'	110.6(3)
C8-C9	1.533(6)	N7-C8-C15	112.8(3)
C9-C9A	1.483(7)	C15-C8-C9	106.2(3)
C9A-C14A	1.353(6)	*Torsion angles(°)*	
C14A-C14B	1.498(6)	C6-N5-C4A-C14C	14.3(6)
C14B-C14C	1.518(6)	C4A-N5-C6-N7	-44.6(5)
C4A-C14C	1.412(6)	C8-N7-C6-N5	-59.0(4)
N7-C14B	1.489(5)	C6-N7-C8-C9	-170.4(3)
Bond angles(°)		N7-C8-C9-C9A	-43.9(5)
N5-C4A-C14C	120.3(4)	C8-C9-C9A-C14A	12.1(6)
C4A-N5-C6	119.1(4)	C9-C9A-C14A-C14B	-2.8(7)
N5-C6-N7	110.2(4)	C9A-C14A-C14B-C14C	-101.5(5)
C6-N7-C8	112.8(3)	C14A-C14B-C14C-C4A	146.1(4)
N7-C8-C9	110.8(3)	N5-C4A-C14C-C14B	-3.5(6)
C9A-C9-C8	110.4(4)	C8-N7-C14B-C14A	-55.3(4)
C14A-C9A-C9	122.1(4)	C8-N7-C14B-C14C	71.4(4)
C14A-C14B-C14C	114.3(3)	C6-N7-C14B-C14A	-178.7(3)
C4A-C14C-C14B	118.5(4)	C6-N7-C14B-C14C	-52.1(4)
C6-N7-C14B	108.4(3)	C6-N7-C8-C15	-51.4(5)
C8-N7-C14B	109.5(3)	C2'-C1'-C6-N5	28.3(6)
**N-H…O and C-H…O type hydrogen bonds (for D-H…A hydrogen bond: D…A distance/ H…A distance / D-H-A angle)**			**CSD average(SD)**
N14-H14…O22<br>S21A=O22...H14 / S21B=O22...H14<br>S21A=O22...N14 / S21B=O22...N14<br>occupancy: S21A / S21B		1.987 / 2.814(6) / 161<br>136.2 / 96.9<br>130.6(3) / 91.4 (4)<br>0.823 / 0.177	2.03(14) / 2.85(9) / 160 (12)<br>129(13)<br>130(14)**
N5-H5…O26<br>S25A=O26...H5 / S25B=O26...H5<br>S25A=O26...N5 / S25B=O26...N5<br>occupancy: S25A / S25B		2.11(6) / 2.990(6) / 161(5)<br>142(2) / 173(1)<br>145.0(4) / 167.8(4)<br>0.569 / 0.431	2.03(14) / 2.85(9) / 160 (12)<br>129(13)<br>130(14) **
C27-H27C...O15 [a]		2.39 / 3.25(1) / 148.5	
C28-H28F… O8' [b]		2.55 / 3.28(1) / 133.5	
C6-H6…O15		2.544 / 3.204(6) / 124.6	
C8'-H8'C…O9'		2.385 / 2.955(7) / 117.58	

*For hydrogen bonds of the disordered DMSO molecules, geometry of the acceptor group and related occupancy is also shown.

**Data for 492 organic structures containingnon-disordered DMSO molecules.

[a] O15 atom of the symmetry equivalent molecule trans formed by [x-1, y, z]

[b] O8′ atom of the symmetry equivalent molecule trans formed by [x-1/2, -y+3/2, -z+1]

References

[1] Dolomanov, O.V., Bourhis, L.J., Gildea, R.J, Howard, J.A.K., Puschmann, H. (2009), J. Appl. Cryst. 42, 339-341.

[2] Sheldrick, G.M. (2015). Acta Cryst. C71, 3-8.

[3] Macrae, C. F., Edgington, P. R., McCabe, P., Pidcock, E., Shields, G P., Taylor, R., Towler M., van de Streek, J., J. Appl. Cryst., 39, 453-457, 2006

[4] Spek, A.L. (2009) Acta Cryst, D65, 148-155.

[5] Aurora J. Cruz-Cabeza, Graeme M. Day and William Jones (2011), Phys. Chem. 13,12808–12816

[6] C. R. Groom, I. J. Bruno, M. P. Lightfoot and S. C. Ward, Acta Cryst. (2016). B72, 171-179
